# Detection of Human Papillomavirus Infection in Patients with Vaginal Intraepithelial Neoplasia

**DOI:** 10.1371/journal.pone.0167386

**Published:** 2016-12-01

**Authors:** Cristina Lamos, Charlotte Mihaljevic, Sebastian Aulmann, Thomas Bruckner, Christoph Domschke, Markus Wallwiener, Carmen Paringer, Herbert Fluhr, Sarah Schott, Christine Dinkic, Janina Brucker, Michael Golatta, Lisa Gensthaler, Michael Eichbaum, Christof Sohn, Joachim Rom

**Affiliations:** 1 Department of Dermatology, Ludwigshafen Hospital, Ludwigshafen, Germany; 2 Department of Gynecology and Obstetrics, University of Heidelberg, Heidelberg, Germany; 3 Practice for Pathology, Frankfurt, Germany; 4 Institute of Medical Biometry and Informatics, University of Heidelberg, Heidelberg, Germany; 5 University of Vienna, Vienna, Austria; 6 Department of Gynecology and Obstetrics, Helios Dr. Horst Schmidt Kliniken, Wiesbaden, Germany; Queen Mary Hospital, HONG KONG

## Abstract

**Introduction:**

Vaginal intraepithelial neoplasia (VAIN) is a pre-malignant lesion, potentially leading to vaginal cancer. It is a rare disease, representing less than 1% of all intraepithelial neoplasia of the female genital tract. Similar to cervical intraepithelial neoplasia (CIN), there are three different grades of VAIN. VAIN 1 is also known as a low-grade squamous intraepithelial lesion (LSIL), whereas VAIN 2 and VAIN 3 both represent high-grade squamous intraepithelial lesions (HSIL). Risk factors for the development of VAIN are similar to those for cervical neoplasia, i.e. promiscuity, starting sexual activity at an early age, tobacco consumption and infection with human papillomavirus (HPV). However, compared to other intraepithelial neoplasia such as CIN or VIN (vulvar intraepithelial neoplasia), there still is little understanding about the natural course of VAIN and its capacity for pro- or regression. Furthermore, there is controversial data about the HPV detection rate in VAIN lesions.

**Patients and Methods:**

67 patients with histologically confirmed VAIN, who were diagnosed between 2003 and 2011 at the University Women´s Hospital of Heidelberg Germany, were included in this study. The biopsies of all participating patients were subjected to HPV genotyping. GP-E6/E7 Nested Multiplex PCR (NMPCR) was used to identify and genotype HPV. Eighteen pairs of type-specific nested PCR primers were assessed to detect the following "high-risk" HPV genotypes: 16, 18, 31, 33, 35, 39, 45, 51, 52, 56, 58, 59, 66 and 68, as well as the "low-risk" genotypes 6/11, 42, 43 and 44. The data was analyzed with the software SAS (Statistical Analysis System).

**Results:**

All 67 cases were eligible for DNA analysis. The median age was 53 years. The largest group with 53% (n = 36) was formed by women, who were first diagnosed with VAIN between the age of 41 to 60 years. 50% (n = 37) of the patients presented a VAIN in the upper 1/3 of the vagina. 58 (87%) were diagnosed with HSIL (VAIN). The median age in patients with LSIL (VAIN) was 53 years and in patients with HSIL (VAIN) 53.5 years. 12 women (18%) had an immunosuppression. HPV positivity was confirmed in 37 patients (55%). Except for a single patient, who had a triple infection with HPV types 6/11, 16 and 68, only infections with one single HPV genotype were detected. An infection with the HPV genotypes 31, 39, 45, 51, 58, 59, 66, 42, 43 and 44 couldn’t be found in any of the patients. In 28 patients with diagnosed VAIN, an infection with HPV 16 could be shown, 24 (86%) of them were diagnosed with a HSIL (VAIN). 16 (24%) women presented condylomata and 13 of them (81%) had a positive HPV status. However, only 47% of the women without condylomata presented a positive HPV status, resulting in a significant correlation (p = 0.0164) between condylomata and HPV infection. In 28 of all 67 patients (42%), recurrence of the neoplasia occurred.

**Conclusion:**

HPV 16 is the main virus-type to be associated with the development of a VAIN. Also, HPV 16 infection, VIN or condylomata acuminata in the past medical history seemed to be significant factors for early relapse.

## Introduction

Vaginal intraepithelial neoplasia (VAIN) is a pre-malignant lesion, potentially leading to vaginal cancer. VAIN is rare, representing less than 1% of all intraepithelial neoplasia of the female genital tract [1]. However, diagnosis of VAIN and other intraepithelial neoplasia such as vulvar (VIN), cervical (CIN) or anal intraepithelial neoplasia (AIN) have increased steadily over the past decades due to increased awareness and improved screening methods [2].

Similar to CIN, there are three grades of VAIN according to the depth of epithelial involvement. Whilst VAIN 1 only affects the lower one-third of epithelium, VAIN 2 involves the lower two-thirds and VAIN 3 more than two-thirds or the complete epithelium. VAIN 1 is also known as a low-grade squamous intraepithelial lesion (LSIL), whereas VAIN 2 and VAIN 3 both represent a high-grade squamous intraepithelial lesion (HSIL) [3].

VAIN can affect women of any age, but it is more common in women above 50. Interestingly, age and grade of disease correlate in the sense that women of a higher age suffer from higher grades of VAIN [2]. Risk factors for the development of VAIN are similar to those for cervical neoplasia, i.e. promiscuity, starting sexual activity at an early age, tobacco consumption and human papillomavirus (HPV) infection. Furthermore, one major risk factor is a history of cervical neoplasia. In addition, women suffering from condylomata acuminata exhibit a higher incidence of VAIN compared to controls [4]. However, compared to other intraepithelial neoplasia such as CIN or VIN there is still little knowledge about the natural course of VAIN and its capacity for pro- or regression.

Furthermore, there is controversial data about HPV detection rate in VAIN lesions. Chao et al reported a 69.3% detection rate of HPV in VAIN lesions [5], whereas other studies revealed a higher rate up to 90–100% [6] [7].

## Patients and Methods

67 patients with histologically confirmed vaginal intraepithelial neoplasia (VAIN) and clinical examination between 2003 and 2011 at the University Women´s Hospital of Heidelberg, Germany, were included in this study. All patients were referred by the gynecologist to University Hospital due to a suspicious vaginal finding. VAIN was diagnosed according to guidelines, using colposcopy, followed by biopsy of the suspicious areas, which were revealed through application of acetic acid and Schiller's Iodine test. All parts of the vagina are thoroughly examined according to an examination scheme viewing the vagina clockwise starting at 3 o'clock. Hematoxylin-eosin sections were made from the biopsies and reviewed by an experienced pathologist. In uncertain cases, immunohistochemical analysis of p16 (INK4a) was additionally performed. We included only patients who were free of any suspicious area 8–12 weeks after initial diagnose or treatment. According to the WHO classification preinvasive lesions of the vaginal squamous epithelium are differentiated into LSIL (VAIN 1) and HSIL (VAIN 2 and 3) [3]. Patients with LSIL (VAIN) were closely surveyed due to the often spontaneous regression. All patients with HSIL (VAIN) received CO_2_ laser vaporization as a primary therapy. The treatment was done consistently between providers (same spot size, power, depth of destruction, margins of destruction). We defined recurrence as detection of new lesions 8–12 weeks after initial therapy at earliest. In all cases the recurrence was histological confirmed by vaginal biopsy. All patients gave their written consent for analysis of their tissue. The study was approved by the ethical committee of the university of Heidelberg (S-277/2015).

### HPV genotyping

Before HPV-testing all biopsies were reviewed by an independent pathologist. Data regarding the patient’s history and neoplasia status were collected retrospectively from the clinical documentation system. The biopsies of all patients, who were included in this study were subjected to HPV genotyping. GP-E6/E7 Nested Multiplex PCR (NMPCR) was used to identify and genotype HPV [8]. The primers (forward: GP E6-3F, reverse: GP-E7-5B and GP-E7-6B) and the PCR enabled the initial amplification of the DNA of 38 of the most common mucosal HPV types [9]. Eighteen pairs of type-specific, nested PCR primers were assessed to detect the following "high-risk" HPV genotypes: 16, 18, 31, 33, 35, 39, 45, 51, 52, 56, 58, 59, 66 and 68, as well as "low-risk" genotypes 6/11, 42, 43 and 44. For the HPV genotypes 6 and 11, only a single specific primer was used due to their high sequence homology and biological similarity. We did perform PCR with E6/E7 consensus primers as a primary PCR followed by a multiplexed secound round of PCR with type-specific primer pools (i.e. nested multiplex-PCR, NMPCR). Primary E6/E7 PCR-products were not analysed separately by gel-electrophoresis, as PCR product yield is typically low when using these primers on paraffin-embedded tissue [9]. The nested multiplex PCR assay used in this study has a similar specificity and even slightly higher sensitivity compared with commercial probe-based assays as documented in a panel-based laboratory validation study [10]. Its validation has further been documented in direct comparison with MY11/09 and GP5/6 consensus primer PCR followed by direct DNA sequencing [9, 11].

### DNA extraction

The samples, embedded in paraffin, were cut with a layer thickness of 7μm. Xylol (AnalaR^®^, NORMAPUR^®^, VWR, Vienna) was added and heated to 55°C. After 10 minutes, the liquid was removed and the previous step repeated. Next, xylol was replaced by 100% ethanol and the samples were left to rest for 10 minutes twice. Between each step, the tissues were centrifuged at 13300G. After removal of alcohol, the tissues were incubated at 37°C. 0,788g Tris-HCl (50 mM, Serva, Heidelberg), 0,0372g EDTA (1 mM, Carl Roth, Karlsruhe) and 0.5 ml Tween 20 (0.5%, Carl Roth, Karlsruhe) were added to a vessel. 50 - 75ml distilled water was added to the mixture and adjusted to a pH of 8.5. 5μl proteinase K (Fermentas, St. Leon-Rot) was added. The samples were then incubated overnight at 56°C. The next day the product was heated to 95°C for 5 minutes. As a next step 5μl were removed from this solution and transferred to a glass cuvette. Eventually, the DNA concentration was determined photometrically (NanoDrop ND-1000 Spectro Photometer). Subsequently, the solution was diluted with nuclease-free water (Fermentas, St. Leon-Rot) to 50ng/μl, after which 2.5 μl DNA were extracted for PCR.

### PCR with E6/E7 consensus primer

2,5μl DNA were assessed with 9,25μl water (nuclease free H_2_O, Fermentas, St. Leon-Rot) and 0,75μl 6P primer (10μM) (primer mix: 6P E6-3F, GP E7-5B, GP E7-6B) and 12,5μl "MasterMix" consisting of deoxynucleotide, Mg^2+^, 10-fold concentrated polymerase buffer solution, water and Taq polymerase. The Taq activation and denaturation of the DNA took place when heated to 94°C, in the first step for 4, then repeated for 1 minute. The primer hybridization was carried out at 40°C for 1 minute, the elongation phase at 72°C for 2 minutes initially and then again for 5 minutes. A total of 40 cycles were performed.

### Second PCR with type-specific nested multiplex primer

Amplification of the GP-E6/E7 PCR products with type-specific primers lead to virus genotype identification. 11,25μl water, 0,75μl primer pools I-IV (per PCR approach) and 12,5μl "MasterMix" were added to 0,5μl E6/E7 product. The Taq activation and denaturation of the DNA were carried out again at 94°C for an initial 4 minutes, then again for 30 seconds. The primer hybridization took place at 56°C for 30 seconds. Finally, the elongation phase was carried out at 72°C, initially for 45 seconds, and in a second step for 4 minutes. A total of 35 cycles were performed.

### Agarose gel electrophoresis

Separation of nucleic acid strands allows determination of the amplicon length, which leads to specific identification of HPV types. The agarose powder (3g to 100ml buffer) was dissolved in 1 × Tris-borate-EDTA (TBA) buffer and heated to near-boiling point. After brief cooling, 10μl "Gel Red" per 100ml buffer was added. The solution was poured into a cast, after which a comb was placed to create wells for loading. Next, the gel was layered with 1x TBE buffer in an electrophoresis chamber and loaded. Furthermore, 5μl PCR product with 2μl 40% glycerol were added on a microtiter plate and applied to the gel. As a molecular weight marker served 50 bp. The term was 1 hour at a voltage of 100 volts. Finally, the gel was removed from the chamber and photographed under UV light.

### Statistics

Data were analysed with the software SAS (Statistical Analysis System). Categorical variables were compared by chi-square test or Fisher's exact test. p-values < 0.05 were considered statistically significant. Due to the small number of cases and partially missing values a multivariate analysis was not performed [12].

## Results

All 67 cases were eligible for DNA analysis. The median age was 53 years (range 26–84 years). Subgroup analysis revealed a median age in patients with LSIL (VAIN) of 53 (range 28–66 years) and HSIL (VAIN) of 53.5 years (range 26–84 years). The largest group with 53% (n = 36) was formed by women, who were first diagnosed with VAIN between the age of 41 to 60 years. 50% (n = 37) of the patients presented with VAIN in the upper 1/3 of the vagina. 58 (87%) were diagnosed with HSIL (VAIN). According to conventional histology, only 6 cases were unclear, which meant that additional p16 (INK4a) staining was necessary. In all 6 cases p16 (INK4a) was detectable. In two cases no HPV infection could be shown, both patients had a HSIL. Three patients were HPV16 and one patient HPV 33 positive. Overall positive HPV status was confirmed in 37 patients (55%). 6 (66,7%) patients with LSIL (VAIN) were HPV positive and 31 (53,4%) of the patients with HSIL (VAIN). The median age of the group with detected HPV infection was 53 years (range 26–79). It differs only slightly from that of the HPV negative group (54 years, range 26–84). Detailed patient characteristics are shown in Table 1.

**Table 1:** Patient characteristics.

Characteristics		n	%
Age (years)	20–30	6	9.0
	31–40	4	6.0
	41–50	18	26.9
	51–60	18	26.9
	61–70	13	19.4
	71–80	7	10.4
	81–90	1	1.5
	Median HPV (+)	53 (range 26–79)	
	Median HPV (-)	54 (range 26–84)	
HPV	positive	37	55.2
	negative	30	44.8
HPV-positive	LSIL (VAIN)	6	16,2
	HSIL (VAIN)	31	83.8
Risk factors	Nicotine	16	23.9
	*Immunosuppression*	*12*	*18*.*0*
Localization	Multifocal	15	22.4
	Lower 1/3	2	3.0
	Middle 1/3	13	19.4
	Upper 1/3	37	55.2
Grade	LSIL (VAIN)	9	13.0
	HSIL (VAIN)	58	87.0

12 women (18%) had an immunosuppression—4 of those received chemotherapy, 2 had an HIV infection, 3 suffered from diabetes mellitus type 1 or 2, and 3 patients had an autoimmune disease or history of transplantation with immunosuppressant medication.

Except for a single patient who had a triple infection with HPV types 16, 6/11 and 68, only infections with one single HPV genotype were diagnosed. The genotype distribution is shown in Table 2. In none of the patients, an infection with the HPV genotypes 31, 39, 45, 51, 58, 59, 66, 42, 43 and 44 could be found. In 28 patients with diagnosed VAIN, a HPV 16 infection could be shown; 24 (86%) of them were diagnosed with HSIL (VAIN).

**Table 2:** HPV type distribution.

HPV type	LSIL (VAIN)	HSIL (VAIN)	Total
	n (%)	n (%)	
**6/11**	0 (0)	2 (100)	2
**16**	4 (14)	24 (86)	28
**18**	0 (0)	2 (100)	2
**33**	0 (0)	2 (100)	2
**35**	0 (0)	1 (100)	1
**52**	1 (100)	0 (0)	1
**56**	1 (50)	1 (50)	2
**68**	1 (100)	0 (0)	1

17 patients of this study presented a simultaneous or previous VIN. Twelve (71%) of those developed VAIN recurrence, whilst only 16 of the 50 patients (32%) without a VIN in the medical history did (p = 0.0053). 32 patients (48%) presented a simultaneous or previous cervical intraepithelial neoplasia (CIN). 10 patients of those patients developed a recurrence. In 18 of 35 patients without CIN the VAIN recurred (p = 0,094). In 10 out of 16 women with a VAIN recurrence a history of condylomata was known (p = 0,054). In one patient a vaginal carcinoma could be found with first diagnose of VAIN.

Patients underwent follow-up examinations with a median duration of 12.5 months (range 1–45,2 months). A follow-up examination was scheduled every 2–3 months. In 28 of 67 patients (42%) recurrence of the VAIN occurred (Table 3).

**Table 3:** Recurrences and HPV infection.

VAIN grade	Primary diagnosis	Recurrence		HPV infection		Median Disease-free survival
	n	n	%	n	%	months (range)
**LSIL (VAIN)**	9	*3*	33%	*3*	*100%*	9 (8.5–31.2)
**HSIL (VAIN)**	58	25	43%	*17*	*68%*	13.05 (3.3–48.1)
**VAIN**	67	28	42%	20	71%	12 (3.3–48.1)

Of the 28 patients, who suffered a recurrence, 20 (71%) had an HPV infection. In comparison, only 17 of the 39 (44%) recurrence free women had a HPV infection (p = 0.0238), indicating a significant correlation between HPV infection and recurrence of disease.

The median time from initial diagnosis to recurrence was 12 months (range 3.3 months to 48.1 months). Of 58 patients with HSIL (VAIN), 25 (43%) developed a recurrence. Recurrent vaginal intraepithelial neoplasia was highly related to HPV infection (p = 0,0238). Only one patient progressed to vaginal carcinoma after one year. None of the other patients progressed to a higher grade of VAIN.

The only patient with a low-risk HPV type 6/11 infection remained recurrence free, as well as two patients with HPV type 56. A significant correlation between HPV 16 infection an VAIN recurrence could be found. 16 (57%) out of 28 patients with a HPV 16 infection VAIN was recurrent (p = 0.0351). Also patients with HPV types 18, 33, 35 and 52 respectively, developed a recurrence. Furthermore, the women with a triple HPV infection with types 16, 6/11 and 68 also developed a recurrence.

## Discussion

In accordance with other studies we were able to demonstrate that the median age of women suffering from VAIN was 53 years [13, 14]. Furthermore, we were able to confirm that patients with LSIL (VAIN) were slightly younger compared to patients having HSIL (VAIN) [2]. In addition, we could demonstrate that there was no significant difference of age in patients with HPV associated VAIN compared to non-HPV associated VAIN.

Other studies have revealed that VAIN is most frequently localised in the upper third of the vagina [13, 15]. This was confirmed in our study with 55% of VAIN lesions primarily located in the upper third of the vagina. Only 20% of VAIN lesion were localised in the middle third and 3% in the lower third. Compared to previous studies by Dodge et al., who described multifocal lesions in 61% of VAIN, [16] the percentage in our cohort was significantly lower (22%).

According to the study of Dodge et al., a strong association between VAIN and coexisting CIN or VIN was present in 65% and 10% of cases, respectively [16].

In addition, 25% of patients were either simultaneously diagnosed with VIN or had a VIN in the past and 48% of patients were diagnosed with CIN respectively. In summary, VAIN frequently presents not as a single pathological phenomenon in 60% of patients it is accompanied with other intraepithelial neoplasia of the lower genital tract. Patients with immune deficiency show a higher risk for developing multiple lesions of the lower genital tract [17–19], which could be supported in our study. 58% of immunosuppressed women had another neoplasia of the lower genital tract. In immunosuppressed patients, 50% of the lesions showed an association with HPV. In contrast to other studies where immunosuppressed patients tended to have a rather atypical HPV genotype, an infection with HPV 16 was found most commonly [20].

There are heterogeneous results concerning the association of VAIN and HPV infection rate. Regarding to this diversity, non-standardised and differing approaches in HPV identification seem to be the leading issue. Smith et al reported a HPV identification rate of 98,5% in 66 LSIL (VAIN) cases and 92,6% in 166 HSIL (VAIN) cases [6]. Similarly, De Vuyst et al demonstrated an infection rate of LSIL (VAIN) in 107 VAIN cases and in 90,1% of 191 HSIL (VAIN) cases [7]. In contrast to these two studies, Chao et al reported a lower HPV detection rate of merely 69,3% (273 of 394 VAIN cases) in an monocentric study, using Ki-67 immunostaining [14].

The cellular tumor suppressor gene p16 (INK4a) is an important biomarker for HPV-associated intraepithelial neoplasia. Klaes et al. found an overexpression of p16(INK4a) in all examined LSIL (CIN) lesions—except for those being caused by "low-risk" HPV types—as well as in all HSIL (CIN) lesions. There was no expression in healthy cervical epithelium or inflammatory altered epithelium [21]. In this study, HPV PCR testing and in unclear situations additional p16INK4a immunostaining was performed to identify HPV-associated intraepithelial neoplasia. An association with HPV was seen in 55% of all cases (37 of 67). Chao et al observed a significantly higher HPV infection rate in cases of HSIL (VAIN) compared to cases of LSIL (VAIN) (58,2% versus 41,8%). This observation could not be verified in our study. 66,7% of LSIL (VAIN) cases and 53,4% of HSIL (VAIN) cases were HPV associated.

Similar to the HPV detection rate, our HPV type analysis revealed different data compared to previous reports. The most common type of HPV in VAIN lesions is generally believed to be HPV 16. De Vuyst et al described HPV 16 in 57,6% of HSIL (VAIN) cases, Smith et al even showed a rate of 65,8% [6] [7]. In our current study, however, we discovered HPV 16 in 86% of HPV associated HSIL (VAIN). Further HPV types discovered in this study were HPV 18 (6,5%), HPV 33 (6,5%), HPV 35 (3,2%), HPV 56 (6,5%), compared to HPV 58, 52, 39 and 53 in other studies [6] [7].

Condylomata acuminata represent the most common benign genital tract tumours, originating from infection with low risk HPV types such as HPV type 6 and 11 [22]. Histological distinction of flat condylomas from low-grade intraepithelial neoplasia may be difficult in cases showing overlapping morphological features. We did however not identify any HPV6/11-infections in any of the included cases of VaIN1 indicating that these lesions indeed are low-grade intraepithelial neoplasia and not condylomas as the latter typically are positive for HPV6/11-DNA. In this study, 24% of VAIN patients showed a medical history of condylomata acuminata. In other studies, the number varies from 16%-30% [16] [4]. In this study, an HPV infection rate of 81% could be detected in women with condylomata acuminata in their past medical history, whilst HPV infection rate of only 47% was seen in women without condylomata in their past. This leads to a significant correlation (p = 0,0164) between occurrence of condylomata in the past and persistent HPV infection. This fits perfectly to the observation of several authors, that condylomata in the past represent a risk factor for developing VAIN 3 [4, 23]. In this study 81% of patients with a history of condylomata suffered from HSIL (VAIN).

The prevalence of vaginal carcinoma varies in different areas. Whereas in Eastern and Western Asia the prevalence rate is 0,2/100000, in Southern Asia and the Caribbean seems to be a higher prevalence rate (0,7/100000) due to high HPV infection rate [24, 25]. Irrespectively of the grade of VAIN, VAIN can progress into vaginal carcinoma in 2–12%, although exact pathomechanism remains unclear [16]. Time period between occurrence of VAIN and progression to vaginal carcinoma differs, depending on different follow-up periods. Aho et al reported 2 of 23 VAIN patients (9%) developing vaginal carcinoma within 3 years without any treatment [26]. In this study, 2 of 67 patients (3%) with VAIN suffered from vaginal carcinoma: one patient developed vaginal carcinoma one year after diagnosis of VAIN, the other patient showed simultaneous vaginal carcinoma at the time VAIN was diagnosed and it remains unclear, whether this carcinoma derived from VAIN or developed independently.

For some authors a premalignant or malignant cervical lesion is an stronger indication for hysterectomy [27–29] than bleeding problems [1, 4, 30] due to the risk for developing VAIN. One study showed no significant difference between the incidence of VAIN in women with previous hysterectomy due to benign, premalignant or malignant reasons, irrespectively [31]. In this study, 42 of 67 patients (63%) had a hysterectomy in their past, 69% of those were performed because of CIN or cervical carcinoma, in 31%, hysterectomy was performed due to bleeding problems or uterine fibroids. This data is concordant with previous reports [1, 32] [27]. In our study, the mean time between hysterectomy and diagnosis of VAIN was 89 months. Depending on indication for hysterectomy, differences could be detected. Those patients with hysterectomy due to CIN or an invasive tumour developed VAIN earlier (mean 58,3 months) compared to those whose hysterectomy was performed due to benign or bleeding problems (mean 185,1 months). This differentiation was also seen by Robinson et al, whose study showed a mean development time for VAIN of 75 months after hysterectomy in those patients with CIN or VIN in their past medical history, whereas patients without neoplasia developed VAIN 157 months after hysterectomy [1]. This fact also underlines the risk of patients with intraepithelial neoplasia for developing premalignant lesions in other parts of the lower genital tract.

However, the closer follow-up of these patients after hysterectomy due to intraepithelial neoplasia may partially cause this difference in the mean development time of VAIN as those patients might be monitored more closely.

In the past there have been several attempts to identify risk factors for relapse, such as nicotine abuse, grading of VAIN, localisation, age and many others [29]. Neither of these studies could show a significant correlation. In this study, we examined several factors and were able to identify HPV association, especially with HPV 16, VIN or condylomata acuminata in the past medical history to be significant factors for relapse.

## Conclusion

HPV 16 is the main virus-type to be associated with the development of VAIN. Also, repeated HPV 16 infection, VIN or condylomata acuminata in the past medical history seem to be significant risk factors for relapse.

## Supporting Information

S1 Table Raw patient data minimum. (XLSX)

S2 Table Statistical analysis I. (RTF)

S3 Table Statistical analysis II. (RTF)

S4 Table Statistical analysis III. (RTF)

S5 Table Statistical analysis IV. (RTF)

S6 Table Tables and Figures Results typecast. (DOCX)
