# Supplementary material for: Detection of Human Papillomavirus Infection in Patients with Vaginal Intraepithelial Neoplasia
Source: PLoS One. 2016 Dec 1;11(12):e0167386. doi: 10.1371/journal.pone.0167386 (PMC5132291; doi:10.1371/journal.pone.0167386)
Supplement: S2 Table — (RTF) [file pone.0167386.s002.rtf]

VIN	Frequency	Percent	Cumulative
Frequency	Cumulative
Percent	
1	2	11.76	2	11.76	
2	2	11.76	4	23.53	
3	13	76.47	17	100.00	


Frequency Missing = 50	


vain	Frequency	Percent	Cumulative
Frequency	Cumulative
Percent	
1	9	13.43	9	13.43	
2	10	14.93	19	28.36	
3	48	71.64	67	100.00	


HPV Assoziation	
HPV_Assoziation	Frequency	Percent	Cumulative
Frequency	Cumulative
Percent	
ja	37	55.22	37	55.22	
nein	30	44.78	67	100.00	


HPV Typ	
HPV_Typ	Frequency	Percent	Cumulative
Frequency	Cumulative
Percent	
nein	30	44.78	30	44.78	
6/11	1	1.49	31	46.27	
16	28	41.79	59	88.06	
18	2	2.99	61	91.04	
33	2	2.99	63	94.03	
35	1	1.49	64	95.52	
52	1	1.49	65	97.01	
56	2	2.99	67	100.00	


HPV Typ1	
HPV_Typ1	Frequency	Percent	Cumulative
Frequency	Cumulative
Percent	
nein	66	98.51	66	98.51	
68	1	1.49	67	100.00	


HPV Typ2	
HPV_Typ2	Frequency	Percent	Cumulative
Frequency	Cumulative
Percent	
nein	30	44.78	30	44.78	
6/11	1	1.49	31	46.27	
16	28	41.79	59	88.06	
sonstige	8	11.94	67	100.00	


Condylome	
Condylome	Frequency	Percent	Cumulative
Frequency	Cumulative
Percent	
ja	16	23.88	16	23.88	
nein	51	76.12	67	100.00	


Nikotin	
Nikton	Frequency	Percent	Cumulative
Frequency	Cumulative
Percent	
ja	16	23.88	16	23.88	
nein	50	74.63	66	98.51	
2	1	1.49	67	100.00	


Immunsupp	
Immunsupp	Frequency	Percent	Cumulative
Frequency	Cumulative
Percent	
nein	52	77.61	52	77.61	
unbekannt	3	4.48	55	82.09	
ja	12	17.91	67	100.00	


Lokalisation VaIN	
Lokalisation_VaIN	Frequency	Percent	Cumulative
Frequency	Cumulative
Percent	
1	15	22.39	15	22.39	
3	2	2.99	17	25.37	
4	13	19.40	30	44.78	
5	37	55.22	67	100.00	


Lokalisation VIN	
Lokalisation_VIN	Frequency	Percent	Cumulative
Frequency	Cumulative
Percent	
1	62	92.54	62	92.54	
6	5	7.46	67	100.00	


Multifokal	Frequency	Percent	Cumulative
Frequency	Cumulative
Percent	
nein	67	100.00	67	100.00	


ZnVaginalCA	Frequency	Percent	Cumulative
Frequency	Cumulative
Percent	
nein	67	100.00	67	100.00	


ZnVulvaCA	Frequency	Percent	Cumulative
Frequency	Cumulative
Percent	
nein	67	100.00	67	100.00	


ZnCervixCA	Frequency	Percent	Cumulative
Frequency	Cumulative
Percent	
nein	67	100.00	67	100.00	


ZnMammaCA	Frequency	Percent	Cumulative
Frequency	Cumulative
Percent	
nein	67	100.00	67	100.00	


Therapie VaIN	
Therapie_VaIN	Frequency	Percent	Cumulative
Frequency	Cumulative
Percent	
Laser	65	97.01	65	97.01	
totale Kolp.	2	2.99	67	100.00	


Therapie nach Rezidiv	
Therapie_nach_Rezidiv	Frequency	Percent	Cumulative
Frequency	Cumulative
Percent	
keine	39	58.21	39	58.21	
Laser	23	34.33	62	92.54	
partielle Kolp.	5	7.46	67	100.00	


rezidivvain	Frequency	Percent	Cumulative
Frequency	Cumulative
Percent	
nein	39	58.21	39	58.21	
ja	28	41.79	67	100.00	

Variable	Label	Mean	Std Dev	Minimum	Median	Maximum	
Alter_bei_Erk
reztimeVAIN	Alter bei Erk
	53.4
43.0	14.1
33.4	26.0
3.3	53.0
30.0	84.0
125.3	

Table of HPV_Assoziation by VIN	
HPV_Assoziation(HPV Assoziation)	VIN	
Frequency
Percent
Row Pct
Col Pct	1	2	3	Total	
ja	1
5.88
9.09
50.00	1
5.88
9.09
50.00	9
52.94
81.82
69.23	11
64.71

	
nein	1
5.88
16.67
50.00	1
5.88
16.67
50.00	4
23.53
66.67
30.77	6
35.29

	
Total	2
11.76	2
11.76	13
76.47	17
100.00	
Frequency Missing = 50	


Statistics for Table of HPV_Assoziation by VIN	


Statistic	DF	Value	Prob	
Chi-Square	2	0.4953	0.7806	
Likelihood Ratio Chi-Square	2	0.4810	0.7862	
Mantel-Haenszel Chi-Square	1	0.4071	0.5235	
Phi Coefficient		0.1707		
Contingency Coefficient		0.1683		
Cramer's V		0.1707		
WARNING: 83% of the cells have expected counts less
than 5. Chi-Square may not be a valid test.	

Effective Sample Size = 17
Frequency Missing = 50	

WARNING: 75% of the data are missing.	


Table of HPV_Assoziation by vain	
HPV_Assoziation(HPV Assoziation)	vain	
Frequency
Percent
Row Pct
Col Pct	1	2	3	Total	
ja	6
8.96
16.22
66.67	4
5.97
10.81
40.00	27
40.30
72.97
56.25	37
55.22

	
nein	3
4.48
10.00
33.33	6
8.96
20.00
60.00	21
31.34
70.00
43.75	30
44.78

	
Total	9
13.43	10
14.93	48
71.64	67
100.00	


Statistics for Table of HPV_Assoziation by vain	


Statistic	DF	Value	Prob	
Chi-Square	2	1.4343	0.4881	
Likelihood Ratio Chi-Square	2	1.4414	0.4864	
Mantel-Haenszel Chi-Square	1	0.0335	0.8547	
Phi Coefficient		0.1463		
Contingency Coefficient		0.1448		
Cramer's V		0.1463		
WARNING: 50% of the cells have expected counts less
than 5. Chi-Square may not be a valid test.	

Sample Size = 67	


Table of HPV_Assoziation by Condylome	
HPV_Assoziation(HPV Assoziation)	Condylome(Condylome)	
Frequency
Percent
Row Pct
Col Pct	ja	nein	Total	
ja	13
19.40
35.14
81.25	24
35.82
64.86
47.06	37
55.22

	
nein	3
4.48
10.00
18.75	27
40.30
90.00
52.94	30
44.78

	
Total	16
23.88	51
76.12	67
100.00	


Statistics for Table of HPV_Assoziation by Condylome	


Statistic	DF	Value	Prob	
Chi-Square	1	5.7580	0.0164	
Likelihood Ratio Chi-Square	1	6.1821	0.0129	
Continuity Adj. Chi-Square	1	4.4583	0.0347	
Mantel-Haenszel Chi-Square	1	5.6720	0.0172	
Phi Coefficient		0.2932		
Contingency Coefficient		0.2813		
Cramer's V		0.2932		


Fisher's Exact Test	
Cell (1,1) Frequency (F)	13	
Left-sided Pr <= F	0.9974	
Right-sided Pr >= F	0.0155	
		
Table Probability (P)	0.0129	
Two-sided Pr <= P	0.0215	

Sample Size = 67	


Table of HPV_Assoziation by Nikton	
HPV_Assoziation(HPV Assoziation)	Nikton(Nikotin)	
Frequency
Percent
Row Pct
Col Pct	ja	nein	2	Total	
ja	9
13.43
24.32
56.25	27
40.30
72.97
54.00	1
1.49
2.70
100.00	37
55.22

	
nein	7
10.45
23.33
43.75	23
34.33
76.67
46.00	0
0.00
0.00
0.00	30
44.78

	
Total	16
23.88	50
74.63	1
1.49	67
100.00	


Statistics for Table of HPV_Assoziation by Nikton	


Statistic	DF	Value	Prob	
Chi-Square	2	0.8479	0.6545	
Likelihood Ratio Chi-Square	2	1.2246	0.5421	
Mantel-Haenszel Chi-Square	1	0.0235	0.8782	
Phi Coefficient		0.1125		
Contingency Coefficient		0.1118		
Cramer's V		0.1125		
WARNING: 33% of the cells have expected counts less
than 5. Chi-Square may not be a valid test.	

Sample Size = 67	


Table of HPV_Assoziation by Immunsupp	
HPV_Assoziation(HPV Assoziation)	Immunsupp(Immunsupp)	
Frequency
Percent
Row Pct
Col Pct	nein	unbekannt	ja	Total	
ja	26
38.81
70.27
50.00	3
4.48
8.11
100.00	8
11.94
21.62
66.67	37
55.22

	
nein	26
38.81
86.67
50.00	0
0.00
0.00
0.00	4
5.97
13.33
33.33	30
44.78

	
Total	52
77.61	3
4.48	12
17.91	67
100.00	


Statistics for Table of HPV_Assoziation by Immunsupp	


Statistic	DF	Value	Prob	
Chi-Square	2	3.6417	0.1619	
Likelihood Ratio Chi-Square	2	4.7854	0.0914	
Mantel-Haenszel Chi-Square	1	0.8472	0.3573	
Phi Coefficient		0.2331		
Contingency Coefficient		0.2271		
Cramer's V		0.2331		
WARNING: 33% of the cells have expected counts less
than 5. Chi-Square may not be a valid test.	

Sample Size = 67	


Table of HPV_Assoziation by Lokalisation_VaIN	
HPV_Assoziation(HPV Assoziation)	Lokalisation_VaIN(Lokalisation VaIN)	
Frequency
Percent
Row Pct
Col Pct	1	3	4	5	Total	
ja	8
11.94
21.62
53.33	1
1.49
2.70
50.00	8
11.94
21.62
61.54	20
29.85
54.05
54.05	37
55.22

	
nein	7
10.45
23.33
46.67	1
1.49
3.33
50.00	5
7.46
16.67
38.46	17
25.37
56.67
45.95	30
44.78

	
Total	15
22.39	2
2.99	13
19.40	37
55.22	67
100.00	


Statistics for Table of HPV_Assoziation by Lokalisation_VaIN	


Statistic	DF	Value	Prob	
Chi-Square	3	0.2739	0.9649	
Likelihood Ratio Chi-Square	3	0.2761	0.9645	
Mantel-Haenszel Chi-Square	1	0.0063	0.9367	
Phi Coefficient		0.0639		
Contingency Coefficient		0.0638		
Cramer's V		0.0639		
WARNING: 25% of the cells have expected counts less
than 5. Chi-Square may not be a valid test.	

Sample Size = 67	


Table of HPV_Assoziation by Lokalisation_VIN	
HPV_Assoziation(HPV Assoziation)	Lokalisation_VIN(Lokalisation VIN)	
Frequency
Percent
Row Pct
Col Pct	1	6	Total	
ja	34
50.75
91.89
54.84	3
4.48
8.11
60.00	37
55.22

	
nein	28
41.79
93.33
45.16	2
2.99
6.67
40.00	30
44.78

	
Total	62
92.54	5
7.46	67
100.00	


Statistics for Table of HPV_Assoziation by Lokalisation_VIN	


Statistic	DF	Value	Prob	
Chi-Square	1	0.0498	0.8233	
Likelihood Ratio Chi-Square	1	0.0502	0.8227	
Continuity Adj. Chi-Square	1	0.0000	1.0000	
Mantel-Haenszel Chi-Square	1	0.0491	0.8246	
Phi Coefficient		-0.0273		
Contingency Coefficient		0.0273		
Cramer's V		-0.0273		
WARNING: 50% of the cells have expected counts less
than 5. Chi-Square may not be a valid test.	


Fisher's Exact Test	
Cell (1,1) Frequency (F)	34	
Left-sided Pr <= F	0.6003	
Right-sided Pr >= F	0.7497	
		
Table Probability (P)	0.3500	
Two-sided Pr <= P	1.0000	

Sample Size = 67	


Table of HPV_Assoziation by Multifokal	
HPV_Assoziation(HPV Assoziation)	Multifokal	
Frequency
Percent
Row Pct
Col Pct	nein	Total	
ja	37
55.22
100.00
55.22	37
55.22

	
nein	30
44.78
100.00
44.78	30
44.78

	
Total	67
100.00	67
100.00	


Table of HPV_Assoziation by ZnVaginalCA	
HPV_Assoziation(HPV Assoziation)	ZnVaginalCA	
Frequency
Percent
Row Pct
Col Pct	nein	Total	
ja	37
55.22
100.00
55.22	37
55.22

	
nein	30
44.78
100.00
44.78	30
44.78

	
Total	67
100.00	67
100.00	


Table of HPV_Assoziation by ZnVulvaCA	
HPV_Assoziation(HPV Assoziation)	ZnVulvaCA	
Frequency
Percent
Row Pct
Col Pct	nein	Total	
ja	37
55.22
100.00
55.22	37
55.22

	
nein	30
44.78
100.00
44.78	30
44.78

	
Total	67
100.00	67
100.00	


Table of HPV_Assoziation by ZnCervixCA	
HPV_Assoziation(HPV Assoziation)	ZnCervixCA	
Frequency
Percent
Row Pct
Col Pct	nein	Total	
ja	37
55.22
100.00
55.22	37
55.22

	
nein	30
44.78
100.00
44.78	30
44.78

	
Total	67
100.00	67
100.00	


Table of HPV_Assoziation by ZnMammaCA	
HPV_Assoziation(HPV Assoziation)	ZnMammaCA	
Frequency
Percent
Row Pct
Col Pct	nein	Total	
ja	37
55.22
100.00
55.22	37
55.22

	
nein	30
44.78
100.00
44.78	30
44.78

	
Total	67
100.00	67
100.00	


Table of HPV_Assoziation by Therapie_VaIN	
HPV_Assoziation(HPV Assoziation)	Therapie_VaIN(Therapie VaIN)	
Frequency
Percent
Row Pct
Col Pct	Laser	totale Kolp.	Total	
ja	37
55.22
100.00
56.92	0
0.00
0.00
0.00	37
55.22

	
nein	28
41.79
93.33
43.08	2
2.99
6.67
100.00	30
44.78

	
Total	65
97.01	2
2.99	67
100.00	


Statistics for Table of HPV_Assoziation by Therapie_VaIN	


Statistic	DF	Value	Prob	
Chi-Square	1	2.5426	0.1108	
Likelihood Ratio Chi-Square	1	3.2901	0.0697	
Continuity Adj. Chi-Square	1	0.7616	0.3828	
Mantel-Haenszel Chi-Square	1	2.5046	0.1135	
Phi Coefficient		0.1948		
Contingency Coefficient		0.1912		
Cramer's V		0.1948		
WARNING: 50% of the cells have expected counts less
than 5. Chi-Square may not be a valid test.	


Fisher's Exact Test	
Cell (1,1) Frequency (F)	37	
Left-sided Pr <= F	1.0000	
Right-sided Pr >= F	0.1967	
		
Table Probability (P)	0.1967	
Two-sided Pr <= P	0.1967	

Sample Size = 67	


Table of HPV_Assoziation by Therapie_nach_Rezidiv	
HPV_Assoziation(HPV Assoziation)	Therapie_nach_Rezidiv(Therapie nach Rezidiv)	
Frequency
Percent
Row Pct
Col Pct	keine	Laser	partielle Kolp.	Total	
ja	17
25.37
45.95
43.59	17
25.37
45.95
73.91	3
4.48
8.11
60.00	37
55.22

	
nein	22
32.84
73.33
56.41	6
8.96
20.00
26.09	2
2.99
6.67
40.00	30
44.78

	
Total	39
58.21	23
34.33	5
7.46	67
100.00	


Statistics for Table of HPV_Assoziation by Therapie_nach_Rezidiv	


Statistic	DF	Value	Prob	
Chi-Square	2	5.4298	0.0662	
Likelihood Ratio Chi-Square	2	5.5939	0.0610	
Mantel-Haenszel Chi-Square	1	4.9435	0.0262	
Phi Coefficient		0.2847		
Contingency Coefficient		0.2738		
Cramer's V		0.2847		
WARNING: 33% of the cells have expected counts less
than 5. Chi-Square may not be a valid test.	

Sample Size = 67	


Table of HPV_Assoziation by rezidivvain	
HPV_Assoziation(HPV Assoziation)	rezidivvain	
Frequency
Percent
Row Pct
Col Pct	nein	ja	Total	
ja	17
25.37
45.95
43.59	20
29.85
54.05
71.43	37
55.22

	
nein	22
32.84
73.33
56.41	8
11.94
26.67
28.57	30
44.78

	
Total	39
58.21	28
41.79	67
100.00	


Statistics for Table of HPV_Assoziation by rezidivvain	


Statistic	DF	Value	Prob	
Chi-Square	1	5.1083	0.0238	
Likelihood Ratio Chi-Square	1	5.2233	0.0223	
Continuity Adj. Chi-Square	1	4.0445	0.0443	
Mantel-Haenszel Chi-Square	1	5.0321	0.0249	
Phi Coefficient		-0.2761		
Contingency Coefficient		0.2662		
Cramer's V		-0.2761		


Fisher's Exact Test	
Cell (1,1) Frequency (F)	17	
Left-sided Pr <= F	0.0215	
Right-sided Pr >= F	0.9944	
		
Table Probability (P)	0.0159	
Two-sided Pr <= P	0.0280	

Sample Size = 67	

Table of HPV_Assoziation by rezidivvain	
HPV_Assoziation(HPV Assoziation)	rezidivvain	
Frequency
Percent
Row Pct
Col Pct	nein	ja	Total	
ja	17
25.37
45.95
43.59	20
29.85
54.05
71.43	37
55.22

	
nein	22
32.84
73.33
56.41	8
11.94
26.67
28.57	30
44.78

	
Total	39
58.21	28
41.79	67
100.00	


Statistics for Table of HPV_Assoziation by rezidivvain	


Statistic	DF	Value	Prob	
Chi-Square	1	5.1083	0.0238	
Likelihood Ratio Chi-Square	1	5.2233	0.0223	
Continuity Adj. Chi-Square	1	4.0445	0.0443	
Mantel-Haenszel Chi-Square	1	5.0321	0.0249	
Phi Coefficient		-0.2761		
Contingency Coefficient		0.2662		
Cramer's V		-0.2761		


Fisher's Exact Test	
Cell (1,1) Frequency (F)	17	
Left-sided Pr <= F	0.0215	
Right-sided Pr >= F	0.9944	
		
Table Probability (P)	0.0159	
Two-sided Pr <= P	0.0280	

Sample Size = 67	


Table of HPV_Assoziation by vinjn	
HPV_Assoziation(HPV Assoziation)	vinjn	
Frequency
Percent
Row Pct
Col Pct	j	n	Total	
ja	11
16.42
29.73
64.71	26
38.81
70.27
52.00	37
55.22

	
nein	6
8.96
20.00
35.29	24
35.82
80.00
48.00	30
44.78

	
Total	17
25.37	50
74.63	67
100.00	


Statistics for Table of HPV_Assoziation by vinjn	


Statistic	DF	Value	Prob	
Chi-Square	1	0.8283	0.3628	
Likelihood Ratio Chi-Square	1	0.8399	0.3594	
Continuity Adj. Chi-Square	1	0.3941	0.5301	
Mantel-Haenszel Chi-Square	1	0.8159	0.3664	
Phi Coefficient		0.1112		
Contingency Coefficient		0.1105		
Cramer's V		0.1112		


Fisher's Exact Test	
Cell (1,1) Frequency (F)	11	
Left-sided Pr <= F	0.8841	
Right-sided Pr >= F	0.2665	
		
Table Probability (P)	0.1506	
Two-sided Pr <= P	0.4098	

Sample Size = 67	

Table of altersgruppe by HPV_Assoziation	
altersgruppe	HPV_Assoziation(HPV Assoziation)	
Frequency
Percent
Row Pct
Col Pct	ja	nein	Total	
< 50	14
20.90
58.33
37.84	10
14.93
41.67
33.33	24
35.82

	
>= 50	23
34.33
53.49
62.16	20
29.85
46.51
66.67	43
64.18

	
Total	37
55.22	30
44.78	67
100.00	


Statistics for Table of altersgruppe by HPV_Assoziation	


Statistic	DF	Value	Prob	
Chi-Square	1	0.1462	0.7022	
Likelihood Ratio Chi-Square	1	0.1466	0.7018	
Continuity Adj. Chi-Square	1	0.0159	0.8996	
Mantel-Haenszel Chi-Square	1	0.1440	0.7043	
Phi Coefficient		0.0467		
Contingency Coefficient		0.0467		
Cramer's V		0.0467		


Fisher's Exact Test	
Cell (1,1) Frequency (F)	14	
Left-sided Pr <= F	0.7377	
Right-sided Pr >= F	0.4509	
		
Table Probability (P)	0.1886	
Two-sided Pr <= P	0.8000	

Sample Size = 67	


Table of altersgruppe by rezidivvain	
altersgruppe	rezidivvain	
Frequency
Percent
Row Pct
Col Pct	nein	ja	Total	
< 50	16
23.88
66.67
41.03	8
11.94
33.33
28.57	24
35.82

	
>= 50	23
34.33
53.49
58.97	20
29.85
46.51
71.43	43
64.18

	
Total	39
58.21	28
41.79	67
100.00	


Statistics for Table of altersgruppe by rezidivvain	


Statistic	DF	Value	Prob	
Chi-Square	1	1.0996	0.2943	
Likelihood Ratio Chi-Square	1	1.1137	0.2913	
Continuity Adj. Chi-Square	1	0.6246	0.4293	
Mantel-Haenszel Chi-Square	1	1.0832	0.2980	
Phi Coefficient		0.1281		
Contingency Coefficient		0.1271		
Cramer's V		0.1281		


Fisher's Exact Test	
Cell (1,1) Frequency (F)	16	
Left-sided Pr <= F	0.9051	
Right-sided Pr >= F	0.2153	
		
Table Probability (P)	0.1205	
Two-sided Pr <= P	0.3165	

Sample Size = 67	


Table of altersgruppe by vinjn	
altersgruppe	vinjn	
Frequency
Percent
Row Pct
Col Pct	j	n	Total	
< 50	8
11.94
33.33
47.06	16
23.88
66.67
32.00	24
35.82

	
>= 50	9
13.43
20.93
52.94	34
50.75
79.07
68.00	43
64.18

	
Total	17
25.37	50
74.63	67
100.00	


Statistics for Table of altersgruppe by vinjn	


Statistic	DF	Value	Prob	
Chi-Square	1	1.2514	0.2633	
Likelihood Ratio Chi-Square	1	1.2239	0.2686	
Continuity Adj. Chi-Square	1	0.6821	0.4089	
Mantel-Haenszel Chi-Square	1	1.2327	0.2669	
Phi Coefficient		0.1367		
Contingency Coefficient		0.1354		
Cramer's V		0.1367		


Fisher's Exact Test	
Cell (1,1) Frequency (F)	8	
Left-sided Pr <= F	0.9196	
Right-sided Pr >= F	0.2034	
		
Table Probability (P)	0.1230	
Two-sided Pr <= P	0.3801	

Sample Size = 67	

HPV Assoziation	N Obs	Variable	Label	Mean	Std Dev	Minimum	Median	Maximum	
ja	37	Alter_bei_Erk
reztimeVAIN	Alter bei Erk
	52.4
36.4	13.1
32.9	26.0
3.3	53.0
25.3	79.0
125.3	
nein	30	Alter_bei_Erk
reztimeVAIN	Alter bei Erk
	54.8
51.2	15.3
32.7	26.0
7.9	54.0
36.9	84.0
117.0	

Analysis Variable	P-value, Wilcoxon Test (Two-sided)	
Alter_bei_Erk	0.52407	
reztimeVAIN	0.01966	

Table of rezidivvain by VIN	
rezidivvain	VIN	
Frequency
Percent
Row Pct
Col Pct	1	2	3	Total	
nein	0
0.00
0.00
0.00	1
5.88
20.00
50.00	4
23.53
80.00
30.77	5
29.41

	
ja	2
11.76
16.67
100.00	1
5.88
8.33
50.00	9
52.94
75.00
69.23	12
70.59

	
Total	2
11.76	2
11.76	13
76.47	17
100.00	
Frequency Missing = 50	


Statistics for Table of rezidivvain by VIN	


Statistic	DF	Value	Prob	
Chi-Square	2	1.2532	0.5344	
Likelihood Ratio Chi-Square	2	1.7762	0.4114	
Mantel-Haenszel Chi-Square	1	0.3363	0.5620	
Phi Coefficient		0.2715		
Contingency Coefficient		0.2620		
Cramer's V		0.2715		
WARNING: 83% of the cells have expected counts less
than 5. Chi-Square may not be a valid test.	

Effective Sample Size = 17
Frequency Missing = 50	

WARNING: 75% of the data are missing.	


Table of rezidivvain by vain	
rezidivvain	vain	
Frequency
Percent
Row Pct
Col Pct	1	2	3	Total	
nein	6
8.96
15.38
66.67	8
11.94
20.51
80.00	25
37.31
64.10
52.08	39
58.21

	
ja	3
4.48
10.71
33.33	2
2.99
7.14
20.00	23
34.33
82.14
47.92	28
41.79

	
Total	9
13.43	10
14.93	48
71.64	67
100.00	


Statistics for Table of rezidivvain by vain	


Statistic	DF	Value	Prob	
Chi-Square	2	2.9571	0.2280	
Likelihood Ratio Chi-Square	2	3.1435	0.2077	
Mantel-Haenszel Chi-Square	1	1.6176	0.2034	
Phi Coefficient		0.2101		
Contingency Coefficient		0.2056		
Cramer's V		0.2101		
WARNING: 33% of the cells have expected counts less
than 5. Chi-Square may not be a valid test.	

Sample Size = 67	


Table of rezidivvain by Condylome	
rezidivvain	Condylome(Condylome)	
Frequency
Percent
Row Pct
Col Pct	ja	nein	Total	
nein	6
8.96
15.38
37.50	33
49.25
84.62
64.71	39
58.21

	
ja	10
14.93
35.71
62.50	18
26.87
64.29
35.29	28
41.79

	
Total	16
23.88	51
76.12	67
100.00	


Statistics for Table of rezidivvain by Condylome	


Statistic	DF	Value	Prob	
Chi-Square	1	3.7057	0.0542	
Likelihood Ratio Chi-Square	1	3.6742	0.0553	
Continuity Adj. Chi-Square	1	2.6717	0.1021	
Mantel-Haenszel Chi-Square	1	3.6504	0.0561	
Phi Coefficient		-0.2352		
Contingency Coefficient		0.2289		
Cramer's V		-0.2352		


Fisher's Exact Test	
Cell (1,1) Frequency (F)	6	
Left-sided Pr <= F	0.0517	
Right-sided Pr >= F	0.9864	
		
Table Probability (P)	0.0381	
Two-sided Pr <= P	0.0811	

Sample Size = 67	


Table of rezidivvain by Nikton	
rezidivvain	Nikton(Nikotin)	
Frequency
Percent
Row Pct
Col Pct	ja	nein	2	Total	
nein	8
11.94
20.51
50.00	30
44.78
76.92
60.00	1
1.49
2.56
100.00	39
58.21

	
ja	8
11.94
28.57
50.00	20
29.85
71.43
40.00	0
0.00
0.00
0.00	28
41.79

	
Total	16
23.88	50
74.63	1
1.49	67
100.00	


Statistics for Table of rezidivvain by Nikton	


Statistic	DF	Value	Prob	
Chi-Square	2	1.2271	0.5414	
Likelihood Ratio Chi-Square	2	1.5857	0.4526	
Mantel-Haenszel Chi-Square	1	0.8898	0.3455	
Phi Coefficient		0.1353		
Contingency Coefficient		0.1341		
Cramer's V		0.1353		
WARNING: 33% of the cells have expected counts less
than 5. Chi-Square may not be a valid test.	

Sample Size = 67	


Table of rezidivvain by Immunsupp	
rezidivvain	Immunsupp(Immunsupp)	
Frequency
Percent
Row Pct
Col Pct	nein	unbekannt	ja	Total	
nein	31
46.27
79.49
59.62	2
2.99
5.13
66.67	6
8.96
15.38
50.00	39
58.21

	
ja	21
31.34
75.00
40.38	1
1.49
3.57
33.33	6
8.96
21.43
50.00	28
41.79

	
Total	52
77.61	3
4.48	12
17.91	67
100.00	


Statistics for Table of rezidivvain by Immunsupp	


Statistic	DF	Value	Prob	
Chi-Square	2	0.4629	0.7934	
Likelihood Ratio Chi-Square	2	0.4607	0.7942	
Mantel-Haenszel Chi-Square	1	0.3901	0.5323	
Phi Coefficient		0.0831		
Contingency Coefficient		0.0828		
Cramer's V		0.0831		
WARNING: 33% of the cells have expected counts less
than 5. Chi-Square may not be a valid test.	

Sample Size = 67	


Table of rezidivvain by Lokalisation_VaIN	
rezidivvain	Lokalisation_VaIN(Lokalisation VaIN)	
Frequency
Percent
Row Pct
Col Pct	1	3	4	5	Total	
nein	7
10.45
17.95
46.67	0
0.00
0.00
0.00	10
14.93
25.64
76.92	22
32.84
56.41
59.46	39
58.21

	
ja	8
11.94
28.57
53.33	2
2.99
7.14
100.00	3
4.48
10.71
23.08	15
22.39
53.57
40.54	28
41.79

	
Total	15
22.39	2
2.99	13
19.40	37
55.22	67
100.00	


Statistics for Table of rezidivvain by Lokalisation_VaIN	


Statistic	DF	Value	Prob	
Chi-Square	3	5.5026	0.1385	
Likelihood Ratio Chi-Square	3	6.3340	0.0964	
Mantel-Haenszel Chi-Square	1	1.0921	0.2960	
Phi Coefficient		0.2866		
Contingency Coefficient		0.2755		
Cramer's V		0.2866		
WARNING: 25% of the cells have expected counts less
than 5. Chi-Square may not be a valid test.	

Sample Size = 67	


Table of rezidivvain by Lokalisation_VIN	
rezidivvain	Lokalisation_VIN(Lokalisation VIN)	
Frequency
Percent
Row Pct
Col Pct	1	6	Total	
nein	38
56.72
97.44
61.29	1
1.49
2.56
20.00	39
58.21

	
ja	24
35.82
85.71
38.71	4
5.97
14.29
80.00	28
41.79

	
Total	62
92.54	5
7.46	67
100.00	


Statistics for Table of rezidivvain by Lokalisation_VIN	


Statistic	DF	Value	Prob	
Chi-Square	1	3.2427	0.0717	
Likelihood Ratio Chi-Square	1	3.3020	0.0692	
Continuity Adj. Chi-Square	1	1.7675	0.1837	
Mantel-Haenszel Chi-Square	1	3.1943	0.0739	
Phi Coefficient		0.2200		
Contingency Coefficient		0.2149		
Cramer's V		0.2200		
WARNING: 50% of the cells have expected counts less
than 5. Chi-Square may not be a valid test.	


Fisher's Exact Test	
Cell (1,1) Frequency (F)	38	
Left-sided Pr <= F	0.9898	
Right-sided Pr >= F	0.0929	
		
Table Probability (P)	0.0827	
Two-sided Pr <= P	0.1525	

Sample Size = 67	


Table of rezidivvain by Multifokal	
rezidivvain	Multifokal	
Frequency
Percent
Row Pct
Col Pct	nein	Total	
nein	39
58.21
100.00
58.21	39
58.21

	
ja	28
41.79
100.00
41.79	28
41.79

	
Total	67
100.00	67
100.00	


Table of rezidivvain by ZnVaginalCA	
rezidivvain	ZnVaginalCA	
Frequency
Percent
Row Pct
Col Pct	nein	Total	
nein	39
58.21
100.00
58.21	39
58.21

	
ja	28
41.79
100.00
41.79	28
41.79

	
Total	67
100.00	67
100.00	


Table of rezidivvain by ZnVulvaCA	
rezidivvain	ZnVulvaCA	
Frequency
Percent
Row Pct
Col Pct	nein	Total	
nein	39
58.21
100.00
58.21	39
58.21

	
ja	28
41.79
100.00
41.79	28
41.79

	
Total	67
100.00	67
100.00	


Table of rezidivvain by ZnCervixCA	
rezidivvain	ZnCervixCA	
Frequency
Percent
Row Pct
Col Pct	nein	Total	
nein	39
58.21
100.00
58.21	39
58.21

	
ja	28
41.79
100.00
41.79	28
41.79

	
Total	67
100.00	67
100.00	


Table of rezidivvain by ZnMammaCA	
rezidivvain	ZnMammaCA	
Frequency
Percent
Row Pct
Col Pct	nein	Total	
nein	39
58.21
100.00
58.21	39
58.21

	
ja	28
41.79
100.00
41.79	28
41.79

	
Total	67
100.00	67
100.00	


Table of rezidivvain by Therapie_VaIN	
rezidivvain	Therapie_VaIN(Therapie VaIN)	
Frequency
Percent
Row Pct
Col Pct	Laser	totale Kolp.	Total	
nein	37
55.22
94.87
56.92	2
2.99
5.13
100.00	39
58.21

	
ja	28
41.79
100.00
43.08	0
0.00
0.00
0.00	28
41.79

	
Total	65
97.01	2
2.99	67
100.00	


Statistics for Table of rezidivvain by Therapie_VaIN	


Statistic	DF	Value	Prob	
Chi-Square	1	1.4801	0.2238	
Likelihood Ratio Chi-Square	1	2.2086	0.1372	
Continuity Adj. Chi-Square	1	0.2389	0.6250	
Mantel-Haenszel Chi-Square	1	1.4580	0.2273	
Phi Coefficient		-0.1486		
Contingency Coefficient		0.1470		
Cramer's V		-0.1486		
WARNING: 50% of the cells have expected counts less
than 5. Chi-Square may not be a valid test.	


Fisher's Exact Test	
Cell (1,1) Frequency (F)	37	
Left-sided Pr <= F	0.3351	
Right-sided Pr >= F	1.0000	
		
Table Probability (P)	0.3351	
Two-sided Pr <= P	0.5061	

Sample Size = 67	


Table of rezidivvain by Therapie_nach_Rezidiv	
rezidivvain	Therapie_nach_Rezidiv(Therapie nach Rezidiv)	
Frequency
Percent
Row Pct
Col Pct	keine	Laser	partielle Kolp.	Total	
nein	39
58.21
100.00
100.00	0
0.00
0.00
0.00	0
0.00
0.00
0.00	39
58.21

	
ja	0
0.00
0.00
0.00	23
34.33
82.14
100.00	5
7.46
17.86
100.00	28
41.79

	
Total	39
58.21	23
34.33	5
7.46	67
100.00	


Statistics for Table of rezidivvain by Therapie_nach_Rezidiv	


Statistic	DF	Value	Prob	
Chi-Square	2	67.0000	<.0001	
Likelihood Ratio Chi-Square	2	91.0676	<.0001	
Mantel-Haenszel Chi-Square	1	65.9279	<.0001	
Phi Coefficient		1.0000		
Contingency Coefficient		0.7071		
Cramer's V		1.0000		
WARNING: 33% of the cells have expected counts less
than 5. Chi-Square may not be a valid test.	

Sample Size = 67	


Table of rezidivvain by HPV_Assoziation	
rezidivvain	HPV_Assoziation(HPV Assoziation)	
Frequency
Percent
Row Pct
Col Pct	ja	nein	Total	
nein	17
25.37
43.59
45.95	22
32.84
56.41
73.33	39
58.21

	
ja	20
29.85
71.43
54.05	8
11.94
28.57
26.67	28
41.79

	
Total	37
55.22	30
44.78	67
100.00	


Statistics for Table of rezidivvain by HPV_Assoziation	


Statistic	DF	Value	Prob	
Chi-Square	1	5.1083	0.0238	
Likelihood Ratio Chi-Square	1	5.2233	0.0223	
Continuity Adj. Chi-Square	1	4.0445	0.0443	
Mantel-Haenszel Chi-Square	1	5.0321	0.0249	
Phi Coefficient		-0.2761		
Contingency Coefficient		0.2662		
Cramer's V		-0.2761		


Fisher's Exact Test	
Cell (1,1) Frequency (F)	17	
Left-sided Pr <= F	0.0215	
Right-sided Pr >= F	0.9944	
		
Table Probability (P)	0.0159	
Two-sided Pr <= P	0.0280	

Sample Size = 67	

Table of rezidivvain by vinjn	
rezidivvain	vinjn	
Frequency
Percent
Row Pct
Col Pct	j	n	Total	
nein	5
7.46
12.82
29.41	34
50.75
87.18
68.00	39
58.21

	
ja	12
17.91
42.86
70.59	16
23.88
57.14
32.00	28
41.79

	
Total	17
25.37	50
74.63	67
100.00	


Statistics for Table of rezidivvain by vinjn	


Statistic	DF	Value	Prob	
Chi-Square	1	7.7657	0.0053	
Likelihood Ratio Chi-Square	1	7.7835	0.0053	
Continuity Adj. Chi-Square	1	6.2604	0.0123	
Mantel-Haenszel Chi-Square	1	7.6498	0.0057	
Phi Coefficient		-0.3404		
Contingency Coefficient		0.3223		
Cramer's V		-0.3404		


Fisher's Exact Test	
Cell (1,1) Frequency (F)	5	
Left-sided Pr <= F	0.0062	
Right-sided Pr >= F	0.9990	
		
Table Probability (P)	0.0052	
Two-sided Pr <= P	0.0094	

Sample Size = 67	

Table of Nikton by Condylome	
Nikton(Nikotin)	Condylome(Condylome)	
Frequency
Percent
Row Pct
Col Pct	ja	nein	Total	
ja	9
13.64
56.25
56.25	7
10.61
43.75
14.00	16
24.24

	
nein	7
10.61
14.00
43.75	43
65.15
86.00
86.00	50
75.76

	
Total	16
24.24	50
75.76	66
100.00	


Statistics for Table of Nikton by Condylome	


Statistic	DF	Value	Prob	
Chi-Square	1	11.7814	0.0006	
Likelihood Ratio Chi-Square	1	10.6829	0.0011	
Continuity Adj. Chi-Square	1	9.5932	0.0020	
Mantel-Haenszel Chi-Square	1	11.6029	0.0007	
Phi Coefficient		0.4225		
Contingency Coefficient		0.3892		
Cramer's V		0.4225		
WARNING: 25% of the cells have expected counts less
than 5. Chi-Square may not be a valid test.	


Fisher's Exact Test	
Cell (1,1) Frequency (F)	9	
Left-sided Pr <= F	0.9998	
Right-sided Pr >= F	0.0015	
		
Table Probability (P)	0.0013	
Two-sided Pr <= P	0.0015	

Sample Size = 66	

rezidivvain	N Obs	Variable	Label	Mean	Std Dev	Minimum	Median	Maximum	
nein	39	Alter_bei_Erk
reztimeVAIN	Alter bei Erk
	51.8
62.7	15.4
30.1	26.0
25.2	52.0
64.3	79.0
125.3	
ja	28	Alter_bei_Erk
reztimeVAIN	Alter bei Erk
	55.8
15.6	11.9
10.8	28.0
3.3	55.5
12.7	84.0
48.1	

Analysis Variable	P-value, Wilcoxon Test (Two-sided)	
Alter_bei_Erk	0.32740	
reztimeVAIN	0.00000	

Analysis Variable : Alter_bei_Erk Alter bei Erk	
Condylome	N Obs	Mean	Std Dev	Minimum	Median	Maximum	
ja	16	47.8	11.3	26.0	51.0	59.0	
nein	51	55.2	14.5	26.0	55.0	84.0	

Analysis Variable	P-value, Wilcoxon Test (Two-sided)	
Alter_bei_Erk	0.086503	

Model Information	
Data Set	WORK.A	
Dependent Variable	reztimeVAIN	
Censoring Variable	rezidivvain	
Censoring Value(s)	0	
Ties Handling	BRESLOW	


Number of Observations Read
Number of Observations Used	67
67	


Summary of the Number of Event and Censored Values	
Total	Event	Censored	Percent
Censored	
67	28	39	58.21	


Convergence Status	
Convergence criterion (GCONV=1E-8) satisfied.	


Model Fit Statistics	
Criterion	Without
Covariates	With
Covariates	
-2 LOG L	219.436	209.614	
AIC	219.436	215.614	
SBC	219.436	219.611	


Testing Global Null Hypothesis: BETA=0	
Test	Chi-Square	DF	Pr > ChiSq	
Likelihood Ratio	9.8220	3	0.0201	
Score	9.2730	3	0.0259	
Wald	8.8070	3	0.0320	


Analysis of Maximum Likelihood Estimates	
Parameter	DF	Parameter
Estimate	Standard
Error	Chi-Square	Pr > ChiSq	Hazard
Ratio	Label	
Alter_bei_Erk	1	0.02673	0.01529	3.0562	0.0804	1.027	Alter bei Erk	
Condylome	1	-0.61637	0.43882	1.9729	0.1601	0.540	Condylome	
HPV_Assoziation	1	-0.90391	0.43209	4.3763	0.0364	0.405	HPV Assoziation	

Product-Limit Survival Estimates	
reztimeVAIN		Survival	Failure	Survival Standard Error	Number
Failed	Number
Left	
0.000		1.0000	0	0	0	67	
3.267		0.9851	0.0149	0.0148	1	66	
4.067		0.9701	0.0299	0.0208	2	65	
6.067		.	.	.	3	64	
6.067		0.9403	0.0597	0.0289	4	63	
6.200		0.9254	0.0746	0.0321	5	62	
6.500		0.9104	0.0896	0.0349	6	61	
7.767		0.8955	0.1045	0.0374	7	60	
7.933		0.8806	0.1194	0.0396	8	59	
8.400		0.8657	0.1343	0.0417	9	58	
8.467		0.8507	0.1493	0.0435	10	57	
9.100		0.8358	0.1642	0.0453	11	56	
9.967		0.8209	0.1791	0.0468	12	55	
11.233		0.8060	0.1940	0.0483	13	54	
12.533		0.7910	0.2090	0.0497	14	53	
12.900		0.7761	0.2239	0.0509	15	52	
13.200		0.7612	0.2388	0.0521	16	51	
13.700		0.7463	0.2537	0.0532	17	50	
14.233		0.7313	0.2687	0.0542	18	49	
15.900		0.7164	0.2836	0.0551	19	48	
15.967		0.7015	0.2985	0.0559	20	47	
22.667		0.6866	0.3134	0.0567	21	46	
25.200	*	.	.	.	21	45	
25.200	*	.	.	.	21	44	
25.267		0.6710	0.3290	0.0575	22	43	
25.867	*	.	.	.	22	42	
26.667		0.6550	0.3450	0.0583	23	41	
26.900	*	.	.	.	23	40	
27.167		0.6386	0.3614	0.0591	24	39	
28.100	*	.	.	.	24	38	
28.767	*	.	.	.	24	37	
29.400	*	.	.	.	24	36	
29.600	*	.	.	.	24	35	
29.833	*	.	.	.	24	34	
30.033		0.6198	0.3802	0.0603	25	33	
31.200		0.6010	0.3990	0.0613	26	32	
31.867		0.5823	0.4177	0.0622	27	31	
33.367	*	.	.	.	27	30	
33.467	*	.	.	.	27	29	
36.933	*	.	.	.	27	28	
36.933	*	.	.	.	27	27	
42.800	*	.	.	.	27	26	
44.200	*	.	.	.	27	25	
48.133		0.5590	0.4410	0.0639	28	24	
50.967	*	.	.	.	28	23	
53.767	*	.	.	.	28	22	
54.933	*	.	.	.	28	21	
61.600	*	.	.	.	28	20	
64.267	*	.	.	.	28	19	
66.000	*	.	.	.	28	18	
67.500	*	.	.	.	28	17	
68.600	*	.	.	.	28	16	
69.033	*	.	.	.	28	15	
69.767	*	.	.	.	28	14	
78.233	*	.	.	.	28	13	
79.367	*	.	.	.	28	12	
79.900	*	.	.	.	28	11	
80.233	*	.	.	.	28	10	
84.967	*	.	.	.	28	9	
87.967	*	.	.	.	28	8	
89.100	*	.	.	.	28	7	
95.467	*	.	.	.	28	6	
97.067	*	.	.	.	28	5	
98.000	*	.	.	.	28	4	
110.133	*	.	.	.	28	3	
117.000	*	.	.	.	28	2	
121.367	*	.	.	.	28	1	
125.300	*	.	.	.	28	0	

	The marked survival times are censored observations.	

Summary Statistics for Time Variable reztimeVAIN	

Quartile Estimates	
Percent	Point
Estimate	95% Confidence Interval	
		Transform	[Lower	Upper)	
75	.	LOGLOG	.	.	
50	.	LOGLOG	30.033	.	
25	13.700	LOGLOG	8.467	27.167	


Mean	Standard Error	
34.266	2.182	

	The mean survival time and its standard error were underestimated because the largest observation was censored and the estimation was restricted to the largest event time.	


Summary of the Number of Censored and Uncensored Values	
Total	Failed	Censored	Percent
Censored	
67	28	39	58.21	


reztimeVAIN	_CENSOR_	surv	SDF_LCL	ucl	survival	sdf_ucl	se	
0.000	.	1.00000	1.00000	1.00000	100.000	1.00000	0.000000	
3.267	0	0.98507	0.89874	0.99788	98.507	0.99788	0.006535	
4.067	0	0.97015	0.88587	0.99245	97.015	0.99245	0.011378	
6.067	0	0.94030	0.84871	0.97716	94.030	0.97716	0.018809	
6.200	0	0.92537	0.82996	0.96824	92.537	0.96824	0.021871	
6.500	0	0.91045	0.81148	0.95874	91.045	0.95874	0.024638	
7.767	0	0.89552	0.79330	0.94877	89.552	0.94877	0.027168	
7.933	0	0.88060	0.77540	0.93842	88.060	0.93842	0.029500	
8.400	0	0.86567	0.75776	0.92773	86.567	0.92773	0.031663	
8.467	0	0.85075	0.74036	0.91676	85.075	0.91676	0.033679	
9.100	0	0.83582	0.72318	0.90552	83.582	0.90552	0.035563	
9.967	0	0.82090	0.70621	0.89406	82.090	0.89406	0.037330	
11.233	0	0.80597	0.68944	0.88239	80.597	0.88239	0.038990	
12.533	0	0.79104	0.67284	0.87053	79.104	0.87053	0.040551	
12.900	0	0.77612	0.65641	0.85848	77.612	0.85848	0.042022	
13.200	0	0.76119	0.64013	0.84627	76.119	0.84627	0.043408	
13.700	0	0.74627	0.62401	0.83391	74.627	0.83391	0.044714	
14.233	0	0.73134	0.60803	0.82139	73.134	0.82139	0.045945	
15.900	0	0.71642	0.59219	0.80874	71.642	0.80874	0.047104	
15.967	0	0.70149	0.57648	0.79596	70.149	0.79596	0.048195	
22.667	0	0.68657	0.56090	0.78304	68.657	0.78304	0.049221	
25.200	1	0.68657	.	.	68.657	0.78304	0.049221	
25.200	1	0.68657	.	.	68.657	0.78304	0.049221	
25.267	0	0.67096	0.54452	0.76954	67.096	0.76954	0.050295	
25.867	1	0.67096	.	.	67.096	0.76954	0.050295	
26.667	0	0.65499	0.52779	0.75565	65.499	0.75565	0.051360	
26.900	1	0.65499	.	.	65.499	0.75565	0.051360	
27.167	0	0.63861	0.51068	0.74136	63.861	0.74136	0.052420	
28.100	1	0.63861	.	.	63.861	0.74136	0.052420	
28.767	1	0.63861	.	.	63.861	0.74136	0.052420	
29.400	1	0.63861	.	.	63.861	0.74136	0.052420	
29.600	1	0.63861	.	.	63.861	0.74136	0.052420	
29.833	1	0.63861	.	.	63.861	0.74136	0.052420	
30.033	0	0.61983	0.49044	0.72534	61.983	0.72534	0.053831	
31.200	0	0.60105	0.47052	0.70910	60.105	0.70910	0.055129	
31.867	0	0.58227	0.45091	0.69265	58.227	0.69265	0.056318	
33.367	1	0.58227	.	.	58.227	0.69265	0.056318	
33.467	1	0.58227	.	.	58.227	0.69265	0.056318	
36.933	1	0.58227	.	.	58.227	0.69265	0.056318	
36.933	1	0.58227	.	.	58.227	0.69265	0.056318	
42.800	1	0.58227	.	.	58.227	0.69265	0.056318	
44.200	1	0.58227	.	.	58.227	0.69265	0.056318	
48.133	0	0.55897	0.42524	0.67324	55.897	0.67324	0.058299	
50.967	1	.	.	.	55.897	0.67324	0.058299	
53.767	1	.	.	.	55.897	0.67324	0.058299	
54.933	1	.	.	.	55.897	0.67324	0.058299	
61.600	1	.	.	.	55.897	0.67324	0.058299	
64.267	1	.	.	.	55.897	0.67324	0.058299	
66.000	1	.	.	.	55.897	0.67324	0.058299	
67.500	1	.	.	.	55.897	0.67324	0.058299	
68.600	1	.	.	.	55.897	0.67324	0.058299	
69.033	1	.	.	.	55.897	0.67324	0.058299	
69.767	1	.	.	.	55.897	0.67324	0.058299	
78.233	1	.	.	.	55.897	0.67324	0.058299	
79.367	1	.	.	.	55.897	0.67324	0.058299	
79.900	1	.	.	.	55.897	0.67324	0.058299	
80.233	1	.	.	.	55.897	0.67324	0.058299	
84.967	1	.	.	.	55.897	0.67324	0.058299	
87.967	1	.	.	.	55.897	0.67324	0.058299	
89.100	1	.	.	.	55.897	0.67324	0.058299	
95.467	1	.	.	.	55.897	0.67324	0.058299	
97.067	1	.	.	.	55.897	0.67324	0.058299	
98.000	1	.	.	.	55.897	0.67324	0.058299	
110.133	1	.	.	.	55.897	0.67324	0.058299	
117.000	1	.	.	.	55.897	0.67324	0.058299	
121.367	1	.	.	.	55.897	0.67324	0.058299	
125.300	1	.	.	.	55.897	0.67324	0.058299	

Product-Limit Survival Estimates	
reztimeVAIN		Survival	Failure	Survival Standard Error	Number
Failed	Number
Left	
0.000		1.0000	0	0	0	37	
3.267		0.9730	0.0270	0.0267	1	36	
4.067		0.9459	0.0541	0.0372	2	35	
6.067		.	.	.	3	34	
6.067		0.8919	0.1081	0.0510	4	33	
6.200		0.8649	0.1351	0.0562	5	32	
6.500		0.8378	0.1622	0.0606	6	31	
7.767		0.8108	0.1892	0.0644	7	30	
8.400		0.7838	0.2162	0.0677	8	29	
8.467		0.7568	0.2432	0.0705	9	28	
9.967		0.7297	0.2703	0.0730	10	27	
11.233		0.7027	0.2973	0.0751	11	26	
12.533		0.6757	0.3243	0.0770	12	25	
12.900		0.6486	0.3514	0.0785	13	24	
14.233		0.6216	0.3784	0.0797	14	23	
15.900		0.5946	0.4054	0.0807	15	22	
15.967		0.5676	0.4324	0.0814	16	21	
22.667		0.5405	0.4595	0.0819	17	20	
25.200	*	.	.	.	17	19	
25.267		0.5121	0.4879	0.0824	18	18	
28.100	*	.	.	.	18	17	
28.767	*	.	.	.	18	16	
31.200		0.4801	0.5199	0.0832	19	15	
31.867		0.4481	0.5519	0.0836	20	14	
33.367	*	.	.	.	20	13	
42.800	*	.	.	.	20	12	
50.967	*	.	.	.	20	11	
54.933	*	.	.	.	20	10	
61.600	*	.	.	.	20	9	
64.267	*	.	.	.	20	8	
66.000	*	.	.	.	20	7	
68.600	*	.	.	.	20	6	
69.767	*	.	.	.	20	5	
78.233	*	.	.	.	20	4	
79.367	*	.	.	.	20	3	
87.967	*	.	.	.	20	2	
121.367	*	.	.	.	20	1	
125.300	*	.	.	.	20	0	

	The marked survival times are censored observations.	

Summary Statistics for Time Variable reztimeVAIN	

Quartile Estimates	
Percent	Point
Estimate	95% Confidence Interval	
		Transform	[Lower	Upper)	
75	.	LOGLOG	.	.	
50	31.200	LOGLOG	12.900	.	
25	9.967	LOGLOG	6.067	15.900	


Mean	Standard Error	
21.670	1.890	

	The mean survival time and its standard error were underestimated because the largest observation was censored and the estimation was restricted to the largest event time.	

Product-Limit Survival Estimates	
reztimeVAIN		Survival	Failure	Survival Standard Error	Number
Failed	Number
Left	
0.000		1.0000	0	0	0	30	
7.933		0.9667	0.0333	0.0328	1	29	
9.100		0.9333	0.0667	0.0455	2	28	
13.200		0.9000	0.1000	0.0548	3	27	
13.700		0.8667	0.1333	0.0621	4	26	
25.200	*	.	.	.	4	25	
25.867	*	.	.	.	4	24	
26.667		0.8306	0.1694	0.0692	5	23	
26.900	*	.	.	.	5	22	
27.167		0.7928	0.2072	0.0756	6	21	
29.400	*	.	.	.	6	20	
29.600	*	.	.	.	6	19	
29.833	*	.	.	.	6	18	
30.033		0.7488	0.2512	0.0833	7	17	
33.467	*	.	.	.	7	16	
36.933	*	.	.	.	7	15	
36.933	*	.	.	.	7	14	
44.200	*	.	.	.	7	13	
48.133		0.6912	0.3088	0.0947	8	12	
53.767	*	.	.	.	8	11	
67.500	*	.	.	.	8	10	
69.033	*	.	.	.	8	9	
79.900	*	.	.	.	8	8	
80.233	*	.	.	.	8	7	
84.967	*	.	.	.	8	6	
89.100	*	.	.	.	8	5	
95.467	*	.	.	.	8	4	
97.067	*	.	.	.	8	3	
98.000	*	.	.	.	8	2	
110.133	*	.	.	.	8	1	
117.000	*	.	.	.	8	0	

	The marked survival times are censored observations.	

Summary Statistics for Time Variable reztimeVAIN	

Quartile Estimates	
Percent	Point
Estimate	95% Confidence Interval	
		Transform	[Lower	Upper)	
75	.	LOGLOG	.	.	
50	.	LOGLOG	48.133	.	
25	30.033	LOGLOG	13.200	.	


Mean	Standard Error	
40.816	2.665	

	The mean survival time and its standard error were underestimated because the largest observation was censored and the estimation was restricted to the largest event time.	


Summary of the Number of Censored and Uncensored Values	
Stratum	HPV_Assoziation	Total	Failed	Censored	Percent
Censored	
1	ja	37	20	17	45.95	
2	nein	30	8	22	73.33	
Total		67	28	39	58.21	

Testing Homogeneity of Survival Curves for reztimeVAIN over Strata	


Rank Statistics	
HPV_Assoziation	Log-Rank	Wilcoxon	
ja	6.3322	373.00	
nein	-6.3322	-373.00	


Covariance Matrix for the Log-Rank Statistics	
HPV_Assoziation	ja	nein	
ja	6.95924	-6.95924	
nein	-6.95924	6.95924	


Covariance Matrix for the Wilcoxon Statistics	
HPV_Assoziation	ja	nein	
ja	19462.2	-19462.2	
nein	-19462.2	19462.2	


Test of Equality over Strata	
Test	Chi-Square	DF	Pr >
Chi-Square	
Log-Rank	5.7617	1	0.0164	
Wilcoxon	7.1487	1	0.0075	
-2Log(LR)	7.0119	1	0.0081	


Product-Limit Survival Estimates	
reztimeVAIN		Survival	Failure	Survival Standard Error	Number
Failed	Number
Left	
0.000		1.0000	0	0	0	28	
4.067		0.9643	0.0357	0.0351	1	27	
6.067		.	.	.	2	26	
6.067		0.8929	0.1071	0.0585	3	25	
6.200		0.8571	0.1429	0.0661	4	24	
6.500		0.8214	0.1786	0.0724	5	23	
7.767		0.7857	0.2143	0.0775	6	22	
8.400		0.7500	0.2500	0.0818	7	21	
9.967		0.7143	0.2857	0.0854	8	20	
11.233		0.6786	0.3214	0.0883	9	19	
12.533		0.6429	0.3571	0.0906	10	18	
12.900		0.6071	0.3929	0.0923	11	17	
15.900		0.5714	0.4286	0.0935	12	16	
15.967		0.5357	0.4643	0.0942	13	15	
22.667		0.5000	0.5000	0.0945	14	14	
25.200	*	.	.	.	14	13	
28.100	*	.	.	.	14	12	
28.767	*	.	.	.	14	11	
31.200		0.4545	0.5455	0.0962	15	10	
31.867		0.4091	0.5909	0.0967	16	9	
42.800	*	.	.	.	16	8	
50.967	*	.	.	.	16	7	
54.933	*	.	.	.	16	6	
61.600	*	.	.	.	16	5	
64.267	*	.	.	.	16	4	
66.000	*	.	.	.	16	3	
87.967	*	.	.	.	16	2	
121.367	*	.	.	.	16	1	
125.300	*	.	.	.	16	0	

	The marked survival times are censored observations.	

Summary Statistics for Time Variable reztimeVAIN	

Quartile Estimates	
Percent	Point
Estimate	95% Confidence Interval	
		Transform	[Lower	Upper)	
75	.	LOGLOG	31.867	.	
50	26.933	LOGLOG	11.233	.	
25	9.183	LOGLOG	6.067	15.900	


Mean	Standard Error	
21.126	2.194	

	The mean survival time and its standard error were underestimated because the largest observation was censored and the estimation was restricted to the largest event time.	

Product-Limit Survival Estimates	
reztimeVAIN		Survival	Failure	Survival Standard Error	Number
Failed	Number
Left	
0.000		1.0000	0	0	0	2	
3.267		0.5000	0.5000	0.3536	1	1	
78.233	*	.	.	.	1	0	

	The marked survival times are censored observations.	

Summary Statistics for Time Variable reztimeVAIN	

Quartile Estimates	
Percent	Point
Estimate	95% Confidence Interval	
		Transform	[Lower	Upper)	
75	.	LOGLOG	3.267	.	
50	.	LOGLOG	3.267	.	
25	3.267	LOGLOG	3.267	.	


Mean	Standard Error	
3.267	.	

	The mean survival time and its standard error were underestimated because the largest observation was censored and the estimation was restricted to the largest event time.	

Product-Limit Survival Estimates	
reztimeVAIN		Survival	Failure	Survival Standard Error	Number
Failed	Number
Left	
0.000		1.0000	0	0	0	2	
25.267		0.5000	0.5000	0.3536	1	1	
69.767	*	.	.	.	1	0	

	The marked survival times are censored observations.	

Summary Statistics for Time Variable reztimeVAIN	

Quartile Estimates	
Percent	Point
Estimate	95% Confidence Interval	
		Transform	[Lower	Upper)	
75	.	LOGLOG	25.267	.	
50	.	LOGLOG	25.267	.	
25	25.267	LOGLOG	25.267	.	


Mean	Standard Error	
25.267	.	

	The mean survival time and its standard error were underestimated because the largest observation was censored and the estimation was restricted to the largest event time.	

Product-Limit Survival Estimates	
reztimeVAIN		Survival	Failure	Survival Standard Error	Number
Failed	Number
Left	
0.000		1.0000	0	0	0	1	
14.233		0	1.0000	.	1	0	

Summary Statistics for Time Variable reztimeVAIN	

Quartile Estimates	
Percent	Point
Estimate	95% Confidence Interval	
		Transform	[Lower	Upper)	
75	14.233	LOGLOG	.	.	
50	14.233	LOGLOG	.	.	
25	14.233	LOGLOG	.	.	


Mean	Standard Error	
14.233	.	

Product-Limit Survival Estimates	
reztimeVAIN		Survival	Failure	Survival Standard Error	Number
Failed	Number
Left	
0.000		1.0000	0	0	0	1	
8.467		0	1.0000	.	1	0	

Summary Statistics for Time Variable reztimeVAIN	

Quartile Estimates	
Percent	Point
Estimate	95% Confidence Interval	
		Transform	[Lower	Upper)	
75	8.467	LOGLOG	.	.	
50	8.467	LOGLOG	.	.	
25	8.467	LOGLOG	.	.	


Mean	Standard Error	
8.467	.	

Product-Limit Survival Estimates	
reztimeVAIN		Survival	Failure	Survival Standard Error	Number
Failed	Number
Left	
0.000		1.0000	0	0	0	2	
68.600	*	.	.	.	0	1	
79.367	*	.	.	.	0	0	

	The marked survival times are censored observations.	

Summary Statistics for Time Variable reztimeVAIN	

Quartile Estimates	
Percent	Point
Estimate	95% Confidence Interval	
		Transform	[Lower	Upper)	
75	.	LOGLOG	.	.	
50	.	LOGLOG	.	.	
25	.	LOGLOG	.	.	


Mean	Standard Error	
.	.	

Product-Limit Survival Estimates	
reztimeVAIN		Survival	Failure	Survival Standard Error	Number
Failed	Number
Left	
0.000		1.0000	0	0	0	1	
33.367	*	.	.	.	0	0	

	The marked survival times are censored observations.	

Summary Statistics for Time Variable reztimeVAIN	

Quartile Estimates	
Percent	Point
Estimate	95% Confidence Interval	
		Transform	[Lower	Upper)	
75	.	LOGLOG	.	.	
50	.	LOGLOG	.	.	
25	.	LOGLOG	.	.	


Mean	Standard Error	
.	.	

Product-Limit Survival Estimates	
reztimeVAIN		Survival	Failure	Survival Standard Error	Number
Failed	Number
Left	
0.000		1.0000	0	0	0	30	
7.933		0.9667	0.0333	0.0328	1	29	
9.100		0.9333	0.0667	0.0455	2	28	
13.200		0.9000	0.1000	0.0548	3	27	
13.700		0.8667	0.1333	0.0621	4	26	
25.200	*	.	.	.	4	25	
25.867	*	.	.	.	4	24	
26.667		0.8306	0.1694	0.0692	5	23	
26.900	*	.	.	.	5	22	
27.167		0.7928	0.2072	0.0756	6	21	
29.400	*	.	.	.	6	20	
29.600	*	.	.	.	6	19	
29.833	*	.	.	.	6	18	
30.033		0.7488	0.2512	0.0833	7	17	
33.467	*	.	.	.	7	16	
36.933	*	.	.	.	7	15	
36.933	*	.	.	.	7	14	
44.200	*	.	.	.	7	13	
48.133		0.6912	0.3088	0.0947	8	12	
53.767	*	.	.	.	8	11	
67.500	*	.	.	.	8	10	
69.033	*	.	.	.	8	9	
79.900	*	.	.	.	8	8	
80.233	*	.	.	.	8	7	
84.967	*	.	.	.	8	6	
89.100	*	.	.	.	8	5	
95.467	*	.	.	.	8	4	
97.067	*	.	.	.	8	3	
98.000	*	.	.	.	8	2	
110.133	*	.	.	.	8	1	
117.000	*	.	.	.	8	0	

	The marked survival times are censored observations.	

Summary Statistics for Time Variable reztimeVAIN	

Quartile Estimates	
Percent	Point
Estimate	95% Confidence Interval	
		Transform	[Lower	Upper)	
75	.	LOGLOG	.	.	
50	.	LOGLOG	48.133	.	
25	30.033	LOGLOG	13.200	.	


Mean	Standard Error	
40.816	2.665	

	The mean survival time and its standard error were underestimated because the largest observation was censored and the estimation was restricted to the largest event time.	


Summary of the Number of Censored and Uncensored Values	
Stratum	HPV_Typ	Total	Failed	Censored	Percent
Censored	
1	16	28	16	12	42.86	
2	18	2	1	1	50.00	
3	33	2	1	1	50.00	
4	35	1	1	0	0.00	
5	52	1	1	0	0.00	
6	56	2	0	2	100.00	
7	6/11	1	0	1	100.00	
8	nein	30	8	22	73.33	
Total		67	28	39	58.21	

Testing Homogeneity of Survival Curves for reztimeVAIN over Strata	


Rank Statistics	
HPV_Typ	Log-Rank	Wilcoxon	
16	6.0469	344.00	
18	0.4101	38.00	
33	0.0299	-6.00	
35	0.6901	32.00	
52	0.8399	48.00	
56	-1.1499	-56.00	
6/11	-0.5349	-27.00	
nein	-6.3322	-373.00	


Covariance Matrix for the Log-Rank Statistics	
HPV_Typ	16	18	33	35	52	56	6/11	nein	
16	6.37247	-0.20641	-0.34266	-0.11496	-0.06264	-0.40034	-0.18737	-5.05809	
18	-0.20641	0.57596	-0.02038	-0.00559	-0.00278	-0.02593	-0.01137	-0.30349	
33	-0.34266	-0.02038	0.93480	-0.01074	-0.00512	-0.03987	-0.01834	-0.49768	
35	-0.11496	-0.00559	-0.01074	0.30405	-0.00256	-0.01074	-0.00537	-0.15408	
52	-0.06264	-0.00278	-0.00512	-0.00256	0.15706	-0.00512	-0.00256	-0.07626	
56	-0.40034	-0.02593	-0.03987	-0.01074	-0.00512	1.09792	-0.02229	-0.59362	
6/11	-0.18737	-0.01137	-0.01834	-0.00537	-0.00256	-0.02229	0.52330	-0.27601	
nein	-5.05809	-0.30349	-0.49768	-0.15408	-0.07626	-0.59362	-0.27601	6.95924	


Covariance Matrix for the Wilcoxon Statistics	
HPV_Typ	16	18	33	35	52	56	6/11	nein	
16	18076.9	-551.2	-980.3	-396.2	-246.2	-1046.3	-515.2	-14341.7	
18	-551.2	1482.0	-51.9	-19.0	-11.0	-57.9	-28.0	-763.1	
33	-980.3	-51.9	2592.1	-35.9	-19.9	-99.9	-48.9	-1355.1	
35	-396.2	-19.0	-35.9	1034.0	-10.0	-35.9	-18.0	-519.1	
52	-246.2	-11.0	-19.9	-10.0	614.0	-19.9	-10.0	-297.1	
56	-1046.3	-57.9	-99.9	-35.9	-19.9	2780.1	-53.9	-1466.1	
6/11	-515.2	-28.0	-48.9	-18.0	-10.0	-53.9	1394.0	-720.1	
nein	-14341.7	-763.1	-1355.1	-519.1	-297.1	-1466.1	-720.1	19462.2	


Test of Equality over Strata	
Test	Chi-Square	DF	Pr >
Chi-Square	
Log-Rank	14.6529	7	0.0407	
Wilcoxon	15.0734	7	0.0351	
-2Log(LR)*	16.6817	7	0.0196	

	-2Log(LR) test is questionable since some strata have no events.	


Product-Limit Survival Estimates	
reztimeVAIN		Survival	Failure	Survival Standard Error	Number
Failed	Number
Left	
0.000		1.0000	0	0	0	28	
4.067		0.9643	0.0357	0.0351	1	27	
6.067		.	.	.	2	26	
6.067		0.8929	0.1071	0.0585	3	25	
6.200		0.8571	0.1429	0.0661	4	24	
6.500		0.8214	0.1786	0.0724	5	23	
7.767		0.7857	0.2143	0.0775	6	22	
8.400		0.7500	0.2500	0.0818	7	21	
9.967		0.7143	0.2857	0.0854	8	20	
11.233		0.6786	0.3214	0.0883	9	19	
12.533		0.6429	0.3571	0.0906	10	18	
12.900		0.6071	0.3929	0.0923	11	17	
15.900		0.5714	0.4286	0.0935	12	16	
15.967		0.5357	0.4643	0.0942	13	15	
22.667		0.5000	0.5000	0.0945	14	14	
25.200	*	.	.	.	14	13	
28.100	*	.	.	.	14	12	
28.767	*	.	.	.	14	11	
31.200		0.4545	0.5455	0.0962	15	10	
31.867		0.4091	0.5909	0.0967	16	9	
42.800	*	.	.	.	16	8	
50.967	*	.	.	.	16	7	
54.933	*	.	.	.	16	6	
61.600	*	.	.	.	16	5	
64.267	*	.	.	.	16	4	
66.000	*	.	.	.	16	3	
87.967	*	.	.	.	16	2	
121.367	*	.	.	.	16	1	
125.300	*	.	.	.	16	0	

	The marked survival times are censored observations.	

Summary Statistics for Time Variable reztimeVAIN	

Quartile Estimates	
Percent	Point
Estimate	95% Confidence Interval	
		Transform	[Lower	Upper)	
75	.	LOGLOG	31.867	.	
50	26.933	LOGLOG	11.233	.	
25	9.183	LOGLOG	6.067	15.900	


Mean	Standard Error	
21.126	2.194	

	The mean survival time and its standard error were underestimated because the largest observation was censored and the estimation was restricted to the largest event time.	

Product-Limit Survival Estimates	
reztimeVAIN		Survival	Failure	Survival Standard Error	Number
Failed	Number
Left	
0.000		1.0000	0	0	0	1	
33.367	*	.	.	.	0	0	

	The marked survival times are censored observations.	

Summary Statistics for Time Variable reztimeVAIN	

Quartile Estimates	
Percent	Point
Estimate	95% Confidence Interval	
		Transform	[Lower	Upper)	
75	.	LOGLOG	.	.	
50	.	LOGLOG	.	.	
25	.	LOGLOG	.	.	


Mean	Standard Error	
.	.	

Product-Limit Survival Estimates	
reztimeVAIN		Survival	Failure	Survival Standard Error	Number
Failed	Number
Left	
0.000		1.0000	0	0	0	30	
7.933		0.9667	0.0333	0.0328	1	29	
9.100		0.9333	0.0667	0.0455	2	28	
13.200		0.9000	0.1000	0.0548	3	27	
13.700		0.8667	0.1333	0.0621	4	26	
25.200	*	.	.	.	4	25	
25.867	*	.	.	.	4	24	
26.667		0.8306	0.1694	0.0692	5	23	
26.900	*	.	.	.	5	22	
27.167		0.7928	0.2072	0.0756	6	21	
29.400	*	.	.	.	6	20	
29.600	*	.	.	.	6	19	
29.833	*	.	.	.	6	18	
30.033		0.7488	0.2512	0.0833	7	17	
33.467	*	.	.	.	7	16	
36.933	*	.	.	.	7	15	
36.933	*	.	.	.	7	14	
44.200	*	.	.	.	7	13	
48.133		0.6912	0.3088	0.0947	8	12	
53.767	*	.	.	.	8	11	
67.500	*	.	.	.	8	10	
69.033	*	.	.	.	8	9	
79.900	*	.	.	.	8	8	
80.233	*	.	.	.	8	7	
84.967	*	.	.	.	8	6	
89.100	*	.	.	.	8	5	
95.467	*	.	.	.	8	4	
97.067	*	.	.	.	8	3	
98.000	*	.	.	.	8	2	
110.133	*	.	.	.	8	1	
117.000	*	.	.	.	8	0	

	The marked survival times are censored observations.	

Summary Statistics for Time Variable reztimeVAIN	

Quartile Estimates	
Percent	Point
Estimate	95% Confidence Interval	
		Transform	[Lower	Upper)	
75	.	LOGLOG	.	.	
50	.	LOGLOG	48.133	.	
25	30.033	LOGLOG	13.200	.	


Mean	Standard Error	
40.816	2.665	

	The mean survival time and its standard error were underestimated because the largest observation was censored and the estimation was restricted to the largest event time.	

Product-Limit Survival Estimates	
reztimeVAIN		Survival	Failure	Survival Standard Error	Number
Failed	Number
Left	
0.000		1.0000	0	0	0	8	
3.267		0.8750	0.1250	0.1169	1	7	
8.467		0.7500	0.2500	0.1531	2	6	
14.233		0.6250	0.3750	0.1712	3	5	
25.267		0.5000	0.5000	0.1768	4	4	
68.600	*	.	.	.	4	3	
69.767	*	.	.	.	4	2	
78.233	*	.	.	.	4	1	
79.367	*	.	.	.	4	0	

	The marked survival times are censored observations.	

Summary Statistics for Time Variable reztimeVAIN	

Quartile Estimates	
Percent	Point
Estimate	95% Confidence Interval	
		Transform	[Lower	Upper)	
75	.	LOGLOG	14.233	.	
50	.	LOGLOG	3.267	.	
25	11.350	LOGLOG	3.267	.	


Mean	Standard Error	
19.038	3.469	

	The mean survival time and its standard error were underestimated because the largest observation was censored and the estimation was restricted to the largest event time.	


Summary of the Number of Censored and Uncensored Values	
Stratum	HPV_Typ2	Total	Failed	Censored	Percent
Censored	
1	16	28	16	12	42.86	
2	6/11	1	0	1	100.00	
3	nein	30	8	22	73.33	
4	sonstige	8	4	4	50.00	
Total		67	28	39	58.21	

Testing Homogeneity of Survival Curves for reztimeVAIN over Strata	


Rank Statistics	
HPV_Typ2	Log-Rank	Wilcoxon	
16	6.0469	344.00	
6/11	-0.5349	-27.00	
nein	-6.3322	-373.00	
sonstige	0.8202	56.00	


Covariance Matrix for the Log-Rank Statistics	
HPV_Typ2	16	6/11	nein	sonstige	
16	6.37247	-0.18737	-5.05809	-1.12701	
6/11	-0.18737	0.52330	-0.27601	-0.05992	
nein	-5.05809	-0.27601	6.95924	-1.62514	
sonstige	-1.12701	-0.05992	-1.62514	2.81207	


Covariance Matrix for the Wilcoxon Statistics	
HPV_Typ2	16	6/11	nein	sonstige	
16	18076.9	-515.2	-14341.7	-3220.1	
6/11	-515.2	1394.0	-720.1	-158.8	
nein	-14341.7	-720.1	19462.2	-4400.4	
sonstige	-3220.1	-158.8	-4400.4	7779.3	


Test of Equality over Strata	
Test	Chi-Square	DF	Pr >
Chi-Square	
Log-Rank	7.2680	3	0.0638	
Wilcoxon	8.5806	3	0.0354	
-2Log(LR)*	8.4659	3	0.0373	

	-2Log(LR) test is questionable since some strata have no events.	


Product-Limit Survival Estimates	
reztimeVAIN		Survival	Failure	Survival Standard Error	Number
Failed	Number
Left	
0.000		1.0000	0	0	0	12	
6.500		0.9167	0.0833	0.0798	1	11	
9.967		0.8333	0.1667	0.1076	2	10	
15.900		0.7500	0.2500	0.1250	3	9	
25.267		0.6667	0.3333	0.1361	4	8	
27.167		0.5833	0.4167	0.1423	5	7	
29.833	*	.	.	.	5	6	
31.200		0.4861	0.5139	0.1481	6	5	
33.367	*	.	.	.	6	4	
54.933	*	.	.	.	6	3	
79.900	*	.	.	.	6	2	
87.967	*	.	.	.	6	1	
97.067	*	.	.	.	6	0	

	The marked survival times are censored observations.	

Summary Statistics for Time Variable reztimeVAIN	

Quartile Estimates	
Percent	Point
Estimate	95% Confidence Interval	
		Transform	[Lower	Upper)	
75	.	LOGLOG	31.200	.	
50	31.200	LOGLOG	9.967	.	
25	20.583	LOGLOG	6.500	31.200	


Mean	Standard Error	
25.267	2.775	

	The mean survival time and its standard error were underestimated because the largest observation was censored and the estimation was restricted to the largest event time.	

Product-Limit Survival Estimates	
reztimeVAIN		Survival	Failure	Survival Standard Error	Number
Failed	Number
Left	
0.000		1.0000	0	0	0	52	
3.267		0.9808	0.0192	0.0190	1	51	
6.067		.	.	.	2	50	
6.067		0.9423	0.0577	0.0323	3	49	
6.200		0.9231	0.0769	0.0370	4	48	
7.767		0.9038	0.0962	0.0409	5	47	
7.933		0.8846	0.1154	0.0443	6	46	
8.400		0.8654	0.1346	0.0473	7	45	
8.467		0.8462	0.1538	0.0500	8	44	
9.100		0.8269	0.1731	0.0525	9	43	
11.233		0.8077	0.1923	0.0547	10	42	
12.533		0.7885	0.2115	0.0566	11	41	
12.900		0.7692	0.2308	0.0584	12	40	
13.200		0.7500	0.2500	0.0600	13	39	
13.700		0.7308	0.2692	0.0615	14	38	
14.233		0.7115	0.2885	0.0628	15	37	
15.967		0.6923	0.3077	0.0640	16	36	
22.667		0.6731	0.3269	0.0651	17	35	
25.200	*	.	.	.	17	34	
25.200	*	.	.	.	17	33	
25.867	*	.	.	.	17	32	
26.667		0.6520	0.3480	0.0663	18	31	
26.900	*	.	.	.	18	30	
28.100	*	.	.	.	18	29	
28.767	*	.	.	.	18	28	
29.400	*	.	.	.	18	27	
29.600	*	.	.	.	18	26	
30.033		0.6270	0.3730	0.0684	19	25	
31.867		0.6019	0.3981	0.0701	20	24	
33.467	*	.	.	.	20	23	
36.933	*	.	.	.	20	22	
36.933	*	.	.	.	20	21	
42.800	*	.	.	.	20	20	
44.200	*	.	.	.	20	19	
48.133		0.5702	0.4298	0.0732	21	18	
50.967	*	.	.	.	21	17	
53.767	*	.	.	.	21	16	
61.600	*	.	.	.	21	15	
64.267	*	.	.	.	21	14	
66.000	*	.	.	.	21	13	
67.500	*	.	.	.	21	12	
69.033	*	.	.	.	21	11	
69.767	*	.	.	.	21	10	
78.233	*	.	.	.	21	9	
79.367	*	.	.	.	21	8	
80.233	*	.	.	.	21	7	
84.967	*	.	.	.	21	6	
89.100	*	.	.	.	21	5	
95.467	*	.	.	.	21	4	
98.000	*	.	.	.	21	3	
110.133	*	.	.	.	21	2	
117.000	*	.	.	.	21	1	
121.367	*	.	.	.	21	0	

	The marked survival times are censored observations.	

Summary Statistics for Time Variable reztimeVAIN	

Quartile Estimates	
Percent	Point
Estimate	95% Confidence Interval	
		Transform	[Lower	Upper)	
75	.	LOGLOG	.	.	
50	.	LOGLOG	30.033	.	
25	13.450	LOGLOG	8.400	30.033	


Mean	Standard Error	
34.540	2.513	

	The mean survival time and its standard error were underestimated because the largest observation was censored and the estimation was restricted to the largest event time.	

Product-Limit Survival Estimates	
reztimeVAIN		Survival	Failure	Survival Standard Error	Number
Failed	Number
Left	
0.000		1.0000	0	0	0	2	
4.067		0.5000	0.5000	0.3536	1	1	
68.600	*	.	.	.	1	0	

	The marked survival times are censored observations.	

Summary Statistics for Time Variable reztimeVAIN	

Quartile Estimates	
Percent	Point
Estimate	95% Confidence Interval	
		Transform	[Lower	Upper)	
75	.	LOGLOG	4.067	.	
50	.	LOGLOG	4.067	.	
25	4.067	LOGLOG	4.067	.	


Mean	Standard Error	
4.067	.	

	The mean survival time and its standard error were underestimated because the largest observation was censored and the estimation was restricted to the largest event time.	


Summary of the Number of Censored and Uncensored Values	
Stratum	Immunsupp	Total	Failed	Censored	Percent
Censored	
1	ja	12	6	6	50.00	
2	nein	52	21	31	59.62	
3	unbekannt	2	1	1	50.00	
Total		66	28	38	57.58	

Testing Homogeneity of Survival Curves for reztimeVAIN over Strata	


Rank Statistics	
Immunsupp	Log-Rank	Wilcoxon	
ja	0.6508	6.000	
nein	-1.0323	-41.000	
unbekannt	0.3815	35.000	


Covariance Matrix for the Log-Rank Statistics	
Immunsupp	ja	nein	unbekannt	
ja	4.31928	-4.20219	-0.11709	
nein	-4.20219	4.68835	-0.48616	
unbekannt	-0.11709	-0.48616	0.60325	


Covariance Matrix for the Wilcoxon Statistics	
Immunsupp	ja	nein	unbekannt	
ja	11660.2	-11364.6	-295.6	
nein	-11364.6	12584.0	-1219.4	
unbekannt	-295.6	-1219.4	1515.0	


Test of Equality over Strata	
Test	Chi-Square	DF	Pr >
Chi-Square	
Log-Rank	0.3635	2	0.8338	
Wilcoxon	0.8228	2	0.6627	
-2Log(LR)	0.3119	2	0.8556	


Product-Limit Survival Estimates	
reztimeVAIN		Survival	Failure	Survival Standard Error	Number
Failed	Number
Left	
0.000		1.0000	0	0	0	16	
4.067		0.9375	0.0625	0.0605	1	15	
6.067		0.8750	0.1250	0.0827	2	14	
6.500		0.8125	0.1875	0.0976	3	13	
12.900		0.7500	0.2500	0.1083	4	12	
15.900		0.6875	0.3125	0.1159	5	11	
15.967		0.6250	0.3750	0.1210	6	10	
25.200	*	.	.	.	6	9	
25.867	*	.	.	.	6	8	
29.833	*	.	.	.	6	7	
30.033		0.5357	0.4643	0.1326	7	6	
31.200		0.4464	0.5536	0.1373	8	5	
36.933	*	.	.	.	8	4	
50.967	*	.	.	.	8	3	
67.500	*	.	.	.	8	2	
79.900	*	.	.	.	8	1	
97.067	*	.	.	.	8	0	

	The marked survival times are censored observations.	

Summary Statistics for Time Variable reztimeVAIN	

Quartile Estimates	
Percent	Point
Estimate	95% Confidence Interval	
		Transform	[Lower	Upper)	
75	.	LOGLOG	31.200	.	
50	31.200	LOGLOG	12.900	.	
25	14.400	LOGLOG	4.067	31.200	


Mean	Standard Error	
23.233	2.808	

	The mean survival time and its standard error were underestimated because the largest observation was censored and the estimation was restricted to the largest event time.	

Product-Limit Survival Estimates	
reztimeVAIN		Survival	Failure	Survival Standard Error	Number
Failed	Number
Left	
0.000		1.0000	0	0	0	50	
3.267		0.9800	0.0200	0.0198	1	49	
6.067		0.9600	0.0400	0.0277	2	48	
6.200		0.9400	0.0600	0.0336	3	47	
7.767		0.9200	0.0800	0.0384	4	46	
7.933		0.9000	0.1000	0.0424	5	45	
8.400		0.8800	0.1200	0.0460	6	44	
8.467		0.8600	0.1400	0.0491	7	43	
9.100		0.8400	0.1600	0.0518	8	42	
9.967		0.8200	0.1800	0.0543	9	41	
11.233		0.8000	0.2000	0.0566	10	40	
12.533		0.7800	0.2200	0.0586	11	39	
13.200		0.7600	0.2400	0.0604	12	38	
13.700		0.7400	0.2600	0.0620	13	37	
14.233		0.7200	0.2800	0.0635	14	36	
22.667		0.7000	0.3000	0.0648	15	35	
25.200	*	.	.	.	15	34	
25.267		0.6794	0.3206	0.0661	16	33	
26.667		0.6588	0.3412	0.0672	17	32	
26.900	*	.	.	.	17	31	
27.167		0.6376	0.3624	0.0683	18	30	
28.100	*	.	.	.	18	29	
28.767	*	.	.	.	18	28	
29.400	*	.	.	.	18	27	
29.600	*	.	.	.	18	26	
31.867		0.6130	0.3870	0.0700	19	25	
33.367	*	.	.	.	19	24	
33.467	*	.	.	.	19	23	
36.933	*	.	.	.	19	22	
42.800	*	.	.	.	19	21	
44.200	*	.	.	.	19	20	
48.133		0.5824	0.4176	0.0729	20	19	
53.767	*	.	.	.	20	18	
54.933	*	.	.	.	20	17	
61.600	*	.	.	.	20	16	
64.267	*	.	.	.	20	15	
66.000	*	.	.	.	20	14	
68.600	*	.	.	.	20	13	
69.033	*	.	.	.	20	12	
69.767	*	.	.	.	20	11	
78.233	*	.	.	.	20	10	
79.367	*	.	.	.	20	9	
80.233	*	.	.	.	20	8	
84.967	*	.	.	.	20	7	
87.967	*	.	.	.	20	6	
89.100	*	.	.	.	20	5	
95.467	*	.	.	.	20	4	
98.000	*	.	.	.	20	3	
110.133	*	.	.	.	20	2	
117.000	*	.	.	.	20	1	
121.367	*	.	.	.	20	0	

	The marked survival times are censored observations.	

Summary Statistics for Time Variable reztimeVAIN	

Quartile Estimates	
Percent	Point
Estimate	95% Confidence Interval	
		Transform	[Lower	Upper)	
75	.	LOGLOG	.	.	
50	.	LOGLOG	27.167	.	
25	13.700	LOGLOG	8.467	31.867	


Mean	Standard Error	
35.031	2.521	

	The mean survival time and its standard error were underestimated because the largest observation was censored and the estimation was restricted to the largest event time.	


Summary of the Number of Censored and Uncensored Values	
Stratum	Nikton	Total	Failed	Censored	Percent
Censored	
1	ja	16	8	8	50.00	
2	nein	50	20	30	60.00	
Total		66	28	38	57.58	

Testing Homogeneity of Survival Curves for reztimeVAIN over Strata	


Rank Statistics	
Nikton	Log-Rank	Wilcoxon	
ja	1.7349	79.000	
nein	-1.7349	-79.000	


Covariance Matrix for the Log-Rank Statistics	
Nikton	ja	nein	
ja	4.84306	-4.84306	
nein	-4.84306	4.84306	


Covariance Matrix for the Wilcoxon Statistics	
Nikton	ja	nein	
ja	13313.7	-13313.7	
nein	-13313.7	13313.7	


Test of Equality over Strata	
Test	Chi-Square	DF	Pr >
Chi-Square	
Log-Rank	0.6215	1	0.4305	
Wilcoxon	0.4688	1	0.4936	
-2Log(LR)	1.3527	1	0.2448	


Product-Limit Survival Estimates	
reztimeVAIN		Survival	Failure	Survival Standard Error	Number
Failed	Number
Left	
0.000		1.0000	0	0	0	67	
3.267		0.9851	0.0149	0.0148	1	66	
4.067		0.9701	0.0299	0.0208	2	65	
6.067		.	.	.	3	64	
6.067		0.9403	0.0597	0.0289	4	63	
6.200		0.9254	0.0746	0.0321	5	62	
6.500		0.9104	0.0896	0.0349	6	61	
7.767		0.8955	0.1045	0.0374	7	60	
7.933		0.8806	0.1194	0.0396	8	59	
8.400		0.8657	0.1343	0.0417	9	58	
8.467		0.8507	0.1493	0.0435	10	57	
9.100		0.8358	0.1642	0.0453	11	56	
9.967		0.8209	0.1791	0.0468	12	55	
11.233		0.8060	0.1940	0.0483	13	54	
12.533		0.7910	0.2090	0.0497	14	53	
12.900		0.7761	0.2239	0.0509	15	52	
13.200		0.7612	0.2388	0.0521	16	51	
13.700		0.7463	0.2537	0.0532	17	50	
14.233		0.7313	0.2687	0.0542	18	49	
15.900		0.7164	0.2836	0.0551	19	48	
15.967		0.7015	0.2985	0.0559	20	47	
22.667		0.6866	0.3134	0.0567	21	46	
25.200	*	.	.	.	21	45	
25.200	*	.	.	.	21	44	
25.267		0.6710	0.3290	0.0575	22	43	
25.867	*	.	.	.	22	42	
26.667		0.6550	0.3450	0.0583	23	41	
26.900	*	.	.	.	23	40	
27.167		0.6386	0.3614	0.0591	24	39	
28.100	*	.	.	.	24	38	
28.767	*	.	.	.	24	37	
29.400	*	.	.	.	24	36	
29.600	*	.	.	.	24	35	
29.833	*	.	.	.	24	34	
30.033		0.6198	0.3802	0.0603	25	33	
31.200		0.6010	0.3990	0.0613	26	32	
31.867		0.5823	0.4177	0.0622	27	31	
33.367	*	.	.	.	27	30	
33.467	*	.	.	.	27	29	
36.933	*	.	.	.	27	28	
36.933	*	.	.	.	27	27	
42.800	*	.	.	.	27	26	
44.200	*	.	.	.	27	25	
48.133		0.5590	0.4410	0.0639	28	24	
50.967	*	.	.	.	28	23	
53.767	*	.	.	.	28	22	
54.933	*	.	.	.	28	21	
61.600	*	.	.	.	28	20	
64.267	*	.	.	.	28	19	
66.000	*	.	.	.	28	18	
67.500	*	.	.	.	28	17	
68.600	*	.	.	.	28	16	
69.033	*	.	.	.	28	15	
69.767	*	.	.	.	28	14	
78.233	*	.	.	.	28	13	
79.367	*	.	.	.	28	12	
79.900	*	.	.	.	28	11	
80.233	*	.	.	.	28	10	
84.967	*	.	.	.	28	9	
87.967	*	.	.	.	28	8	
89.100	*	.	.	.	28	7	
95.467	*	.	.	.	28	6	
97.067	*	.	.	.	28	5	
98.000	*	.	.	.	28	4	
110.133	*	.	.	.	28	3	
117.000	*	.	.	.	28	2	
121.367	*	.	.	.	28	1	
125.300	*	.	.	.	28	0	

	The marked survival times are censored observations.	

Summary Statistics for Time Variable reztimeVAIN	

Quartile Estimates	
Percent	Point
Estimate	95% Confidence Interval	
		Transform	[Lower	Upper)	
75	.	LOGLOG	.	.	
50	.	LOGLOG	30.033	.	
25	13.700	LOGLOG	8.467	27.167	


Mean	Standard Error	
34.266	2.182	

	The mean survival time and its standard error were underestimated because the largest observation was censored and the estimation was restricted to the largest event time.	


Summary of the Number of Censored and Uncensored Values	
Stratum	ZnCervixCA	Total	Failed	Censored	Percent
Censored	
1	nein	67	28	39	58.21	


Product-Limit Survival Estimates	
reztimeVAIN		Survival	Failure	Survival Standard Error	Number
Failed	Number
Left	
0.000		1.0000	0	0	0	16	
4.067		0.9375	0.0625	0.0605	1	15	
6.500		0.8750	0.1250	0.0827	2	14	
7.767		0.8125	0.1875	0.0976	3	13	
14.233		0.7500	0.2500	0.1083	4	12	
15.900		0.6875	0.3125	0.1159	5	11	
15.967		0.6250	0.3750	0.1210	6	10	
25.200	*	.	.	.	6	9	
30.033		0.5556	0.4444	0.1259	7	8	
31.200		0.4861	0.5139	0.1279	8	7	
31.867		0.4167	0.5833	0.1271	9	6	
33.367	*	.	.	.	9	5	
42.800	*	.	.	.	9	4	
48.133		0.3125	0.6875	0.1312	10	3	
50.967	*	.	.	.	10	2	
61.600	*	.	.	.	10	1	
67.500	*	.	.	.	10	0	

	The marked survival times are censored observations.	

Summary Statistics for Time Variable reztimeVAIN	

Quartile Estimates	
Percent	Point
Estimate	95% Confidence Interval	
		Transform	[Lower	Upper)	
75	.	LOGLOG	31.200	.	
50	31.200	LOGLOG	14.233	.	
25	15.067	LOGLOG	4.067	31.200	


Mean	Standard Error	
30.548	4.482	

	The mean survival time and its standard error were underestimated because the largest observation was censored and the estimation was restricted to the largest event time.	

Product-Limit Survival Estimates	
reztimeVAIN		Survival	Failure	Survival Standard Error	Number
Failed	Number
Left	
0.000		1.0000	0	0	0	51	
3.267		0.9804	0.0196	0.0194	1	50	
6.067		.	.	.	2	49	
6.067		0.9412	0.0588	0.0329	3	48	
6.200		0.9216	0.0784	0.0376	4	47	
7.933		0.9020	0.0980	0.0416	5	46	
8.400		0.8824	0.1176	0.0451	6	45	
8.467		0.8627	0.1373	0.0482	7	44	
9.100		0.8431	0.1569	0.0509	8	43	
9.967		0.8235	0.1765	0.0534	9	42	
11.233		0.8039	0.1961	0.0556	10	41	
12.533		0.7843	0.2157	0.0576	11	40	
12.900		0.7647	0.2353	0.0594	12	39	
13.200		0.7451	0.2549	0.0610	13	38	
13.700		0.7255	0.2745	0.0625	14	37	
22.667		0.7059	0.2941	0.0638	15	36	
25.200	*	.	.	.	15	35	
25.267		0.6857	0.3143	0.0651	16	34	
25.867	*	.	.	.	16	33	
26.667		0.6649	0.3351	0.0664	17	32	
26.900	*	.	.	.	17	31	
27.167		0.6435	0.3565	0.0676	18	30	
28.100	*	.	.	.	18	29	
28.767	*	.	.	.	18	28	
29.400	*	.	.	.	18	27	
29.600	*	.	.	.	18	26	
29.833	*	.	.	.	18	25	
33.467	*	.	.	.	18	24	
36.933	*	.	.	.	18	23	
36.933	*	.	.	.	18	22	
44.200	*	.	.	.	18	21	
53.767	*	.	.	.	18	20	
54.933	*	.	.	.	18	19	
64.267	*	.	.	.	18	18	
66.000	*	.	.	.	18	17	
68.600	*	.	.	.	18	16	
69.033	*	.	.	.	18	15	
69.767	*	.	.	.	18	14	
78.233	*	.	.	.	18	13	
79.367	*	.	.	.	18	12	
79.900	*	.	.	.	18	11	
80.233	*	.	.	.	18	10	
84.967	*	.	.	.	18	9	
87.967	*	.	.	.	18	8	
89.100	*	.	.	.	18	7	
95.467	*	.	.	.	18	6	
97.067	*	.	.	.	18	5	
98.000	*	.	.	.	18	4	
110.133	*	.	.	.	18	3	
117.000	*	.	.	.	18	2	
121.367	*	.	.	.	18	1	
125.300	*	.	.	.	18	0	

	The marked survival times are censored observations.	

Summary Statistics for Time Variable reztimeVAIN	

Quartile Estimates	
Percent	Point
Estimate	95% Confidence Interval	
		Transform	[Lower	Upper)	
75	.	LOGLOG	.	.	
50	.	LOGLOG	27.167	.	
25	13.200	LOGLOG	8.467	.	


Mean	Standard Error	
22.102	1.169	

	The mean survival time and its standard error were underestimated because the largest observation was censored and the estimation was restricted to the largest event time.	


Summary of the Number of Censored and Uncensored Values	
Stratum	Condylome	Total	Failed	Censored	Percent
Censored	
1	ja	16	10	6	37.50	
2	nein	51	18	33	64.71	
Total		67	28	39	58.21	

Testing Homogeneity of Survival Curves for reztimeVAIN over Strata	


Rank Statistics	
Condylome	Log-Rank	Wilcoxon	
ja	3.5225	123.00	
nein	-3.5225	-123.00	


Covariance Matrix for the Log-Rank Statistics	
Condylome	ja	nein	
ja	4.96261	-4.96261	
nein	-4.96261	4.96261	


Covariance Matrix for the Wilcoxon Statistics	
Condylome	ja	nein	
ja	13977.6	-13977.6	
nein	-13977.6	13977.6	


Test of Equality over Strata	
Test	Chi-Square	DF	Pr >
Chi-Square	
Log-Rank	2.5003	1	0.1138	
Wilcoxon	1.0824	1	0.2982	
-2Log(LR)	5.7291	1	0.0167	


Product-Limit Survival Estimates	
reztimeVAIN		Survival	Failure	Survival Standard Error	Number
Failed	Number
Left	
0.000		1.0000	0	0	0	24	
4.067		0.9583	0.0417	0.0408	1	23	
8.467		0.9167	0.0833	0.0564	2	22	
11.233		0.8750	0.1250	0.0675	3	21	
12.900		0.8333	0.1667	0.0761	4	20	
13.700		0.7917	0.2083	0.0829	5	19	
15.900		0.7500	0.2500	0.0884	6	18	
15.967		0.7083	0.2917	0.0928	7	17	
22.667		0.6667	0.3333	0.0962	8	16	
25.200	*	.	.	.	8	15	
25.867	*	.	.	.	8	14	
28.100	*	.	.	.	8	13	
28.767	*	.	.	.	8	12	
29.600	*	.	.	.	8	11	
29.833	*	.	.	.	8	10	
36.933	*	.	.	.	8	9	
50.967	*	.	.	.	8	8	
54.933	*	.	.	.	8	7	
61.600	*	.	.	.	8	6	
79.900	*	.	.	.	8	5	
87.967	*	.	.	.	8	4	
89.100	*	.	.	.	8	3	
97.067	*	.	.	.	8	2	
98.000	*	.	.	.	8	1	
110.133	*	.	.	.	8	0	

	The marked survival times are censored observations.	

Summary Statistics for Time Variable reztimeVAIN	

Quartile Estimates	
Percent	Point
Estimate	95% Confidence Interval	
		Transform	[Lower	Upper)	
75	.	LOGLOG	.	.	
50	.	LOGLOG	15.967	.	
25	15.933	LOGLOG	4.067	.	


Mean	Standard Error	
19.482	1.180	

	The mean survival time and its standard error were underestimated because the largest observation was censored and the estimation was restricted to the largest event time.	

Product-Limit Survival Estimates	
reztimeVAIN		Survival	Failure	Survival Standard Error	Number
Failed	Number
Left	
0.000		1.0000	0	0	0	43	
3.267		0.9767	0.0233	0.0230	1	42	
6.067		.	.	.	2	41	
6.067		0.9302	0.0698	0.0388	3	40	
6.200		0.9070	0.0930	0.0443	4	39	
6.500		0.8837	0.1163	0.0489	5	38	
7.767		0.8605	0.1395	0.0528	6	37	
7.933		0.8372	0.1628	0.0563	7	36	
8.400		0.8140	0.1860	0.0593	8	35	
9.100		0.7907	0.2093	0.0620	9	34	
9.967		0.7674	0.2326	0.0644	10	33	
12.533		0.7442	0.2558	0.0665	11	32	
13.200		0.7209	0.2791	0.0684	12	31	
14.233		0.6977	0.3023	0.0700	13	30	
25.200	*	.	.	.	13	29	
25.267		0.6736	0.3264	0.0716	14	28	
26.667		0.6496	0.3504	0.0730	15	27	
26.900	*	.	.	.	15	26	
27.167		0.6246	0.3754	0.0743	16	25	
29.400	*	.	.	.	16	24	
30.033		0.5986	0.4014	0.0757	17	23	
31.200		0.5725	0.4275	0.0767	18	22	
31.867		0.5465	0.4535	0.0775	19	21	
33.367	*	.	.	.	19	20	
33.467	*	.	.	.	19	19	
36.933	*	.	.	.	19	18	
42.800	*	.	.	.	19	17	
44.200	*	.	.	.	19	16	
48.133		0.5123	0.4877	0.0798	20	15	
53.767	*	.	.	.	20	14	
64.267	*	.	.	.	20	13	
66.000	*	.	.	.	20	12	
67.500	*	.	.	.	20	11	
68.600	*	.	.	.	20	10	
69.033	*	.	.	.	20	9	
69.767	*	.	.	.	20	8	
78.233	*	.	.	.	20	7	
79.367	*	.	.	.	20	6	
80.233	*	.	.	.	20	5	
84.967	*	.	.	.	20	4	
95.467	*	.	.	.	20	3	
117.000	*	.	.	.	20	2	
121.367	*	.	.	.	20	1	
125.300	*	.	.	.	20	0	

	The marked survival times are censored observations.	

Summary Statistics for Time Variable reztimeVAIN	

Quartile Estimates	
Percent	Point
Estimate	95% Confidence Interval	
		Transform	[Lower	Upper)	
75	.	LOGLOG	.	.	
50	.	LOGLOG	26.667	.	
25	12.533	LOGLOG	6.500	30.033	


Mean	Standard Error	
33.243	2.780	

	The mean survival time and its standard error were underestimated because the largest observation was censored and the estimation was restricted to the largest event time.	


Summary of the Number of Censored and Uncensored Values	
Stratum	altersgruppe	Total	Failed	Censored	Percent
Censored	
1	< 50	24	8	16	66.67	
2	>= 50	43	20	23	53.49	
Total		67	28	39	58.21	

Testing Homogeneity of Survival Curves for reztimeVAIN over Strata	


Rank Statistics	
altersgruppe	Log-Rank	Wilcoxon	
< 50	-2.2514	-108.00	
>= 50	2.2514	108.00	


Covariance Matrix for the Log-Rank Statistics	
altersgruppe	< 50	>= 50	
< 50	6.46919	-6.46919	
>= 50	-6.46919	6.46919	


Covariance Matrix for the Wilcoxon Statistics	
altersgruppe	< 50	>= 50	
< 50	18219.8	-18219.8	
>= 50	-18219.8	18219.8	


Test of Equality over Strata	
Test	Chi-Square	DF	Pr >
Chi-Square	
Log-Rank	0.7835	1	0.3761	
Wilcoxon	0.6402	1	0.4236	
-2Log(LR)	0.6997	1	0.4029	
