# Supplementary material for: Detection of Human Papillomavirus Infection in Patients with Vaginal Intraepithelial Neoplasia
Source: PLoS One. 2016 Dec 1;11(12):e0167386. doi: 10.1371/journal.pone.0167386 (PMC5132291; doi:10.1371/journal.pone.0167386)
Supplement: S3 Table — (RTF) [file pone.0167386.s003.rtf]

Table of vain by HPV_Assoziation	
vain	HPV_Assoziation(HPV Assoziation)	
Frequency
Percent
Row Pct
Col Pct	ja	nein	Total	
1	6
8.96
66.67
16.22	3
4.48
33.33
10.00	9
13.43

	
2	4
5.97
40.00
10.81	6
8.96
60.00
20.00	10
14.93

	
3	27
40.30
56.25
72.97	21
31.34
43.75
70.00	48
71.64

	
Total	37
55.22	30
44.78	67
100.00	


Statistics for Table of vain by HPV_Assoziation	


Statistic	DF	Value	Prob	
Chi-Square	2	1.4343	0.4881	
Likelihood Ratio Chi-Square	2	1.4414	0.4864	
Mantel-Haenszel Chi-Square	1	0.0335	0.8547	
Phi Coefficient		0.1463		
Contingency Coefficient		0.1448		
Cramer's V		0.1463		
WARNING: 50% of the cells have expected counts less
than 5. Chi-Square may not be a valid test.	

Sample Size = 67	


Table of vain by HPV_Typ	
vain	HPV_Typ(HPV Typ)	
Frequency
Percent
Row Pct
Col Pct	nein	6/11	16	18	33	35	52	56	Total	
1	3
4.48
33.33
10.00	1
1.49
11.11
100.00	4
5.97
44.44
14.29	0
0.00
0.00
0.00	0
0.00
0.00
0.00	0
0.00
0.00
0.00	1
1.49
11.11
100.00	0
0.00
0.00
0.00	9
13.43

	
2	6
8.96
60.00
20.00	0
0.00
0.00
0.00	3
4.48
30.00
10.71	0
0.00
0.00
0.00	0
0.00
0.00
0.00	0
0.00
0.00
0.00	0
0.00
0.00
0.00	1
1.49
10.00
50.00	10
14.93

	
3	21
31.34
43.75
70.00	0
0.00
0.00
0.00	21
31.34
43.75
75.00	2
2.99
4.17
100.00	2
2.99
4.17
100.00	1
1.49
2.08
100.00	0
0.00
0.00
0.00	1
1.49
2.08
50.00	48
71.64

	
Total	30
44.78	1
1.49	28
41.79	2
2.99	2
2.99	1
1.49	1
1.49	2
2.99	67
100.00	


Statistics for Table of vain by HPV_Typ	


Statistic	DF	Value	Prob	
Chi-Square	14	18.1000	0.2023	
Likelihood Ratio Chi-Square	14	14.2586	0.4306	
Mantel-Haenszel Chi-Square	1	0.0553	0.8141	
Phi Coefficient		0.5198		
Contingency Coefficient		0.4612		
Cramer's V		0.3675		
WARNING: 92% of the cells have expected counts less
than 5. Chi-Square may not be a valid test.	

Sample Size = 67	


Table of vain by HPV_Typ1	
vain	HPV_Typ1(HPV Typ1)	
Frequency
Percent
Row Pct
Col Pct	nein	68	Total	
1	8
11.94
88.89
12.12	1
1.49
11.11
100.00	9
13.43

	
2	10
14.93
100.00
15.15	0
0.00
0.00
0.00	10
14.93

	
3	48
71.64
100.00
72.73	0
0.00
0.00
0.00	48
71.64

	
Total	66
98.51	1
1.49	67
100.00	


Statistics for Table of vain by HPV_Typ1	


Statistic	DF	Value	Prob	
Chi-Square	2	6.5421	0.0380	
Likelihood Ratio Chi-Square	2	4.1154	0.1277	
Mantel-Haenszel Chi-Square	1	4.8895	0.0270	
Phi Coefficient		0.3125		
Contingency Coefficient		0.2983		
Cramer's V		0.3125		
WARNING: 50% of the cells have expected counts less
than 5. Chi-Square may not be a valid test.	

Sample Size = 67	


Table of vain by HPV_Typ2	
vain	HPV_Typ2(HPV Typ2)	
Frequency
Percent
Row Pct
Col Pct	nein	6/11	16	sonstige	Total	
1	3
4.48
33.33
10.00	1
1.49
11.11
100.00	4
5.97
44.44
14.29	1
1.49
11.11
12.50	9
13.43

	
2	6
8.96
60.00
20.00	0
0.00
0.00
0.00	3
4.48
30.00
10.71	1
1.49
10.00
12.50	10
14.93

	
3	21
31.34
43.75
70.00	0
0.00
0.00
0.00	21
31.34
43.75
75.00	6
8.96
12.50
75.00	48
71.64

	
Total	30
44.78	1
1.49	28
41.79	8
11.94	67
100.00	


Statistics for Table of vain by HPV_Typ2	


Statistic	DF	Value	Prob	
Chi-Square	6	7.6777	0.2627	
Likelihood Ratio Chi-Square	6	5.2612	0.5108	
Mantel-Haenszel Chi-Square	1	0.0241	0.8765	
Phi Coefficient		0.3385		
Contingency Coefficient		0.3206		
Cramer's V		0.2394		
WARNING: 75% of the cells have expected counts less
than 5. Chi-Square may not be a valid test.	

Sample Size = 67	


Table of vain by Condylome	
vain	Condylome(Condylome)	
Frequency
Percent
Row Pct
Col Pct	ja	nein	Total	
1	3
4.48
33.33
18.75	6
8.96
66.67
11.76	9
13.43

	
2	3
4.48
30.00
18.75	7
10.45
70.00
13.73	10
14.93

	
3	10
14.93
20.83
62.50	38
56.72
79.17
74.51	48
71.64

	
Total	16
23.88	51
76.12	67
100.00	


Statistics for Table of vain by Condylome	


Statistic	DF	Value	Prob	
Chi-Square	2	0.8936	0.6397	
Likelihood Ratio Chi-Square	2	0.8582	0.6511	
Mantel-Haenszel Chi-Square	1	0.8456	0.3578	
Phi Coefficient		0.1155		
Contingency Coefficient		0.1147		
Cramer's V		0.1155		
WARNING: 33% of the cells have expected counts less
than 5. Chi-Square may not be a valid test.	

Sample Size = 67	


Table of vain by rezidivvain	
vain	rezidivvain	
Frequency
Percent
Row Pct
Col Pct	nein	ja	Total	
1	6
8.96
66.67
15.38	3
4.48
33.33
10.71	9
13.43

	
2	8
11.94
80.00
20.51	2
2.99
20.00
7.14	10
14.93

	
3	25
37.31
52.08
64.10	23
34.33
47.92
82.14	48
71.64

	
Total	39
58.21	28
41.79	67
100.00	


Statistics for Table of vain by rezidivvain	


Statistic	DF	Value	Prob	
Chi-Square	2	2.9571	0.2280	
Likelihood Ratio Chi-Square	2	3.1435	0.2077	
Mantel-Haenszel Chi-Square	1	1.6176	0.2034	
Phi Coefficient		0.2101		
Contingency Coefficient		0.2056		
Cramer's V		0.2101		
WARNING: 33% of the cells have expected counts less
than 5. Chi-Square may not be a valid test.	

Sample Size = 67	


Table of vain by altersgruppe	
vain	altersgruppe	
Frequency
Percent
Row Pct
Col Pct	< 50	>= 50	Total	
1	4
5.97
44.44
16.67	5
7.46
55.56
11.63	9
13.43

	
2	4
5.97
40.00
16.67	6
8.96
60.00
13.95	10
14.93

	
3	16
23.88
33.33
66.67	32
47.76
66.67
74.42	48
71.64

	
Total	24
35.82	43
64.18	67
100.00	


Statistics for Table of vain by altersgruppe	


Statistic	DF	Value	Prob	
Chi-Square	2	0.4963	0.7802	
Likelihood Ratio Chi-Square	2	0.4881	0.7834	
Mantel-Haenszel Chi-Square	1	0.4849	0.4862	
Phi Coefficient		0.0861		
Contingency Coefficient		0.0857		
Cramer's V		0.0861		
WARNING: 33% of the cells have expected counts less
than 5. Chi-Square may not be a valid test.	

Sample Size = 67	

Table of vain by Nikton	
vain	Nikton(Nikotin)	
Frequency
Percent
Row Pct
Col Pct	ja	nein	Total	
1	2
3.03
22.22
12.50	7
10.61
77.78
14.00	9
13.64

	
2	1
1.52
10.00
6.25	9
13.64
90.00
18.00	10
15.15

	
3	13
19.70
27.66
81.25	34
51.52
72.34
68.00	47
71.21

	
Total	16
24.24	50
75.76	66
100.00	


Statistics for Table of vain by Nikton	


Statistic	DF	Value	Prob	
Chi-Square	2	1.4233	0.4908	
Likelihood Ratio Chi-Square	2	1.6402	0.4404	
Mantel-Haenszel Chi-Square	1	0.5024	0.4785	
Phi Coefficient		0.1469		
Contingency Coefficient		0.1453		
Cramer's V		0.1469		
WARNING: 33% of the cells have expected counts less
than 5. Chi-Square may not be a valid test.	

Sample Size = 66	

Product-Limit Survival Estimates	
reztimeVAIN		Survival	Failure	Survival Standard Error	Number
Failed	Number
Left	
0.000		1.0000	0	0	0	9	
8.467		0.8889	0.1111	0.1048	1	8	
9.967		0.7778	0.2222	0.1386	2	7	
28.767	*	.	.	.	2	6	
31.200		0.6481	0.3519	0.1653	3	5	
33.367	*	.	.	.	3	4	
61.600	*	.	.	.	3	3	
69.033	*	.	.	.	3	2	
79.900	*	.	.	.	3	1	
80.233	*	.	.	.	3	0	

	The marked survival times are censored observations.	

Summary Statistics for Time Variable reztimeVAIN	

Quartile Estimates	
Percent	Point
Estimate	95% Confidence Interval	
		Transform	[Lower	Upper)	
75	.	LOGLOG	.	.	
50	.	LOGLOG	8.467	.	
25	31.200	LOGLOG	8.467	.	


Mean	Standard Error	
26.315	3.734	

	The mean survival time and its standard error were underestimated because the largest observation was censored and the estimation was restricted to the largest event time.	

Product-Limit Survival Estimates	
reztimeVAIN		Survival	Failure	Survival Standard Error	Number
Failed	Number
Left	
0.000		1.0000	0	0	0	10	
7.767		0.9000	0.1000	0.0949	1	9	
29.600	*	.	.	.	1	8	
48.133		0.7875	0.2125	0.1340	2	7	
66.000	*	.	.	.	2	6	
67.500	*	.	.	.	2	5	
79.367	*	.	.	.	2	4	
87.967	*	.	.	.	2	3	
89.100	*	.	.	.	2	2	
98.000	*	.	.	.	2	1	
117.000	*	.	.	.	2	0	

	The marked survival times are censored observations.	

Summary Statistics for Time Variable reztimeVAIN	

Quartile Estimates	
Percent	Point
Estimate	95% Confidence Interval	
		Transform	[Lower	Upper)	
75	.	LOGLOG	.	.	
50	.	LOGLOG	7.767	.	
25	.	LOGLOG	7.767	.	


Mean	Standard Error	
44.097	5.416	

	The mean survival time and its standard error were underestimated because the largest observation was censored and the estimation was restricted to the largest event time.	

Product-Limit Survival Estimates	
reztimeVAIN		Survival	Failure	Survival Standard Error	Number
Failed	Number
Left	
0.000		1.0000	0	0	0	48	
3.267		0.9792	0.0208	0.0206	1	47	
4.067		0.9583	0.0417	0.0288	2	46	
6.067		.	.	.	3	45	
6.067		0.9167	0.0833	0.0399	4	44	
6.200		0.8958	0.1042	0.0441	5	43	
6.500		0.8750	0.1250	0.0477	6	42	
7.933		0.8542	0.1458	0.0509	7	41	
8.400		0.8333	0.1667	0.0538	8	40	
9.100		0.8125	0.1875	0.0563	9	39	
11.233		0.7917	0.2083	0.0586	10	38	
12.533		0.7708	0.2292	0.0607	11	37	
12.900		0.7500	0.2500	0.0625	12	36	
13.200		0.7292	0.2708	0.0641	13	35	
13.700		0.7083	0.2917	0.0656	14	34	
14.233		0.6875	0.3125	0.0669	15	33	
15.900		0.6667	0.3333	0.0680	16	32	
15.967		0.6458	0.3542	0.0690	17	31	
22.667		0.6250	0.3750	0.0699	18	30	
25.200	*	.	.	.	18	29	
25.200	*	.	.	.	18	28	
25.267		0.6027	0.3973	0.0709	19	27	
25.867	*	.	.	.	19	26	
26.667		0.5795	0.4205	0.0718	20	25	
26.900	*	.	.	.	20	24	
27.167		0.5554	0.4446	0.0728	21	23	
28.100	*	.	.	.	21	22	
29.400	*	.	.	.	21	21	
29.833	*	.	.	.	21	20	
30.033		0.5276	0.4724	0.0742	22	19	
31.867		0.4998	0.5002	0.0754	23	18	
33.467	*	.	.	.	23	17	
36.933	*	.	.	.	23	16	
36.933	*	.	.	.	23	15	
42.800	*	.	.	.	23	14	
44.200	*	.	.	.	23	13	
50.967	*	.	.	.	23	12	
53.767	*	.	.	.	23	11	
54.933	*	.	.	.	23	10	
64.267	*	.	.	.	23	9	
68.600	*	.	.	.	23	8	
69.767	*	.	.	.	23	7	
78.233	*	.	.	.	23	6	
84.967	*	.	.	.	23	5	
95.467	*	.	.	.	23	4	
97.067	*	.	.	.	23	3	
110.133	*	.	.	.	23	2	
121.367	*	.	.	.	23	1	
125.300	*	.	.	.	23	0	

	The marked survival times are censored observations.	

Summary Statistics for Time Variable reztimeVAIN	

Quartile Estimates	
Percent	Point
Estimate	95% Confidence Interval	
		Transform	[Lower	Upper)	
75	.	LOGLOG	.	.	
50	31.867	LOGLOG	15.967	.	
25	13.050	LOGLOG	6.500	22.667	


Mean	Standard Error	
23.441	1.553	

	The mean survival time and its standard error were underestimated because the largest observation was censored and the estimation was restricted to the largest event time.	


Summary of the Number of Censored and Uncensored Values	
Stratum	vain	Total	Failed	Censored	Percent
Censored	
1	1	9	3	6	66.67	
2	2	10	2	8	80.00	
3	3	48	23	25	52.08	
Total		67	28	39	58.21	

Testing Homogeneity of Survival Curves for reztimeVAIN over Strata	


Rank Statistics	
vain	Log-Rank	Wilcoxon	
1	-1.1379	-64.00	
2	-3.1526	-169.00	
3	4.2905	233.00	


Covariance Matrix for the Log-Rank Statistics	
vain	1	2	3	
1	3.51717	-0.77107	-2.74610	
2	-0.77107	4.15742	-3.38635	
3	-2.74610	-3.38635	6.13245	


Covariance Matrix for the Wilcoxon Statistics	
vain	1	2	3	
1	9640.3	-1938.2	-7702.1	
2	-1938.2	11006.8	-9068.6	
3	-7702.1	-9068.6	16770.7	


Test of Equality over Strata	
Test	Chi-Square	DF	Pr >
Chi-Square	
Log-Rank	3.2701	2	0.1949	
Wilcoxon	3.5402	2	0.1703	
-2Log(LR)	6.4434	2	0.0399	


Product-Limit Survival Estimates	
reztimeVAIN		Survival	Failure	Survival Standard Error	Number
Failed	Number
Left	
0.000		1.0000	0	0	0	19	
7.767		0.9474	0.0526	0.0512	1	18	
8.467		0.8947	0.1053	0.0704	2	17	
9.967		0.8421	0.1579	0.0837	3	16	
28.767	*	.	.	.	3	15	
29.600	*	.	.	.	3	14	
31.200		0.7820	0.2180	0.0969	4	13	
33.367	*	.	.	.	4	12	
48.133		0.7168	0.2832	0.1086	5	11	
61.600	*	.	.	.	5	10	
66.000	*	.	.	.	5	9	
67.500	*	.	.	.	5	8	
69.033	*	.	.	.	5	7	
79.367	*	.	.	.	5	6	
79.900	*	.	.	.	5	5	
80.233	*	.	.	.	5	4	
87.967	*	.	.	.	5	3	
89.100	*	.	.	.	5	2	
98.000	*	.	.	.	5	1	
117.000	*	.	.	.	5	0	

	The marked survival times are censored observations.	

Summary Statistics for Time Variable reztimeVAIN	

Quartile Estimates	
Percent	Point
Estimate	95% Confidence Interval	
		Transform	[Lower	Upper)	
75	.	LOGLOG	.	.	
50	.	LOGLOG	48.133	.	
25	48.133	LOGLOG	7.767	.	


Mean	Standard Error	
40.894	3.738	

	The mean survival time and its standard error were underestimated because the largest observation was censored and the estimation was restricted to the largest event time.	

Product-Limit Survival Estimates	
reztimeVAIN		Survival	Failure	Survival Standard Error	Number
Failed	Number
Left	
0.000		1.0000	0	0	0	48	
3.267		0.9792	0.0208	0.0206	1	47	
4.067		0.9583	0.0417	0.0288	2	46	
6.067		.	.	.	3	45	
6.067		0.9167	0.0833	0.0399	4	44	
6.200		0.8958	0.1042	0.0441	5	43	
6.500		0.8750	0.1250	0.0477	6	42	
7.933		0.8542	0.1458	0.0509	7	41	
8.400		0.8333	0.1667	0.0538	8	40	
9.100		0.8125	0.1875	0.0563	9	39	
11.233		0.7917	0.2083	0.0586	10	38	
12.533		0.7708	0.2292	0.0607	11	37	
12.900		0.7500	0.2500	0.0625	12	36	
13.200		0.7292	0.2708	0.0641	13	35	
13.700		0.7083	0.2917	0.0656	14	34	
14.233		0.6875	0.3125	0.0669	15	33	
15.900		0.6667	0.3333	0.0680	16	32	
15.967		0.6458	0.3542	0.0690	17	31	
22.667		0.6250	0.3750	0.0699	18	30	
25.200	*	.	.	.	18	29	
25.200	*	.	.	.	18	28	
25.267		0.6027	0.3973	0.0709	19	27	
25.867	*	.	.	.	19	26	
26.667		0.5795	0.4205	0.0718	20	25	
26.900	*	.	.	.	20	24	
27.167		0.5554	0.4446	0.0728	21	23	
28.100	*	.	.	.	21	22	
29.400	*	.	.	.	21	21	
29.833	*	.	.	.	21	20	
30.033		0.5276	0.4724	0.0742	22	19	
31.867		0.4998	0.5002	0.0754	23	18	
33.467	*	.	.	.	23	17	
36.933	*	.	.	.	23	16	
36.933	*	.	.	.	23	15	
42.800	*	.	.	.	23	14	
44.200	*	.	.	.	23	13	
50.967	*	.	.	.	23	12	
53.767	*	.	.	.	23	11	
54.933	*	.	.	.	23	10	
64.267	*	.	.	.	23	9	
68.600	*	.	.	.	23	8	
69.767	*	.	.	.	23	7	
78.233	*	.	.	.	23	6	
84.967	*	.	.	.	23	5	
95.467	*	.	.	.	23	4	
97.067	*	.	.	.	23	3	
110.133	*	.	.	.	23	2	
121.367	*	.	.	.	23	1	
125.300	*	.	.	.	23	0	

	The marked survival times are censored observations.	

Summary Statistics for Time Variable reztimeVAIN	

Quartile Estimates	
Percent	Point
Estimate	95% Confidence Interval	
		Transform	[Lower	Upper)	
75	.	LOGLOG	.	.	
50	31.867	LOGLOG	15.967	.	
25	13.050	LOGLOG	6.500	22.667	


Mean	Standard Error	
23.441	1.553	

	The mean survival time and its standard error were underestimated because the largest observation was censored and the estimation was restricted to the largest event time.	


Summary of the Number of Censored and Uncensored Values	
Stratum	vain2	Total	Failed	Censored	Percent
Censored	
1	1,2	19	5	14	73.68	
2	3	48	23	25	52.08	
Total		67	28	39	58.21	

Testing Homogeneity of Survival Curves for reztimeVAIN over Strata	


Rank Statistics	
vain2	Log-Rank	Wilcoxon	
1,2	-4.2905	-233.00	
3	4.2905	233.00	


Covariance Matrix for the Log-Rank Statistics	
vain2	1,2	3	
1,2	6.13245	-6.13245	
3	-6.13245	6.13245	


Covariance Matrix for the Wilcoxon Statistics	
vain2	1,2	3	
1,2	16770.7	-16770.7	
3	-16770.7	16770.7	


Test of Equality over Strata	
Test	Chi-Square	DF	Pr >
Chi-Square	
Log-Rank	3.0018	1	0.0832	
Wilcoxon	3.2371	1	0.0720	
-2Log(LR)	5.3429	1	0.0208	
