# Supplementary material for: Detection of Human Papillomavirus Infection in Patients with Vaginal Intraepithelial Neoplasia
Source: PLoS One. 2016 Dec 1;11(12):e0167386. doi: 10.1371/journal.pone.0167386 (PMC5132291; doi:10.1371/journal.pone.0167386)
Supplement: S4 Table — (RTF) [file pone.0167386.s004.rtf]

Freq Table Z_n__HE * Z_n__CIN Cross-Tabular Freq Table Table of Z_n__HE by Z_n__CIN	
Z_n__HE(Z#n# HE)	Z_n__CIN(Z#n# CIN)	Total	
Frequency
Percent
Row Pct
Col Pct	0	1		
0	15
22.39
35.71
46.88	27
40.30
64.29
77.14	42
62.69

	
1	17
25.37
68.00
53.13	8
11.94
32.00
22.86	25
37.31

	
Total	32
47.76	35
52.24	67
100.00	


Statistics for Table of Z_n__HE by Z_n__CIN	


Chi-Square Tests Statistic	DF	Value	Prob	
Chi-Square	1	6.5474	0.0105	
Likelihood Ratio Chi-Square	1	6.6563	0.0099	
Continuity Adj. Chi-Square	1	5.3173	0.0211	
Mantel-Haenszel Chi-Square	1	6.4496	0.0111	
Phi Coefficient		-0.3126		
Contingency Coefficient		0.2984		
Cramer's V		-0.3126		


Fisher's Exact Test Fisher's Exact Test	
Cell (1,1) Frequency (F)	15	
Left-sided Pr <= F	0.0102	
Right-sided Pr >= F	0.9977	
		
Table Probability (P)	0.0080	
Two-sided Pr <= P	0.0129	

Sample Size = 67	

Freq Table rezidivvain * Z_n__HE Cross-Tabular Freq Table Table of rezidivvain by Z_n__HE	
rezidivvain	Z_n__HE(Z#n# HE)	Total	
Frequency
Percent
Row Pct
Col Pct	0	1		
nein	22
32.84
56.41
52.38	17
25.37
43.59
68.00	39
58.21

	
ja	20
29.85
71.43
47.62	8
11.94
28.57
32.00	28
41.79

	
Total	42
62.69	25
37.31	67
100.00	


Statistics for Table of rezidivvain by Z_n__HE	


Chi-Square Tests Statistic	DF	Value	Prob	
Chi-Square	1	1.5716	0.2100	
Likelihood Ratio Chi-Square	1	1.5950	0.2066	
Continuity Adj. Chi-Square	1	0.9951	0.3185	
Mantel-Haenszel Chi-Square	1	1.5482	0.2134	
Phi Coefficient		-0.1532		
Contingency Coefficient		0.1514		
Cramer's V		-0.1532		


Fisher's Exact Test Fisher's Exact Test	
Cell (1,1) Frequency (F)	22	
Left-sided Pr <= F	0.1593	
Right-sided Pr >= F	0.9354	
		
Table Probability (P)	0.0948	
Two-sided Pr <= P	0.3060	

Sample Size = 67	


Table rezidivvain * Z_n__CIN Cross-Tabular Freq Table Table of rezidivvain by Z_n__CIN	
rezidivvain	Z_n__CIN(Z#n# CIN)	Total	
Frequency
Percent
Row Pct
Col Pct	0	1		
nein	22
32.84
56.41
68.75	17
25.37
43.59
48.57	39
58.21

	
ja	10
14.93
35.71
31.25	18
26.87
64.29
51.43	28
41.79

	
Total	32
47.76	35
52.24	67
100.00	


Statistics for Table of rezidivvain by Z_n__CIN	


Chi-Square Tests Statistic	DF	Value	Prob	
Chi-Square	1	2.7980	0.0944	
Likelihood Ratio Chi-Square	1	2.8263	0.0927	
Continuity Adj. Chi-Square	1	2.0300	0.1542	
Mantel-Haenszel Chi-Square	1	2.7563	0.0969	
Phi Coefficient		0.2044		
Contingency Coefficient		0.2002		
Cramer's V		0.2044		


Fisher's Exact Test Fisher's Exact Test	
Cell (1,1) Frequency (F)	22	
Left-sided Pr <= F	0.9732	
Right-sided Pr >= F	0.0768	
		
Table Probability (P)	0.0499	
Two-sided Pr <= P	0.1370	

Sample Size = 67	


Table rezidivvain * Multifokal Cross-Tabular Freq Table Table of rezidivvain by Multifokal	
rezidivvain	Multifokal	Total	
Frequency
Percent
Row Pct
Col Pct	nein		
nein	39
58.21
100.00
58.21	39
58.21

	
ja	28
41.79
100.00
41.79	28
41.79

	
Total	67
100.00	67
100.00	

Freq Table rezidivvain * Z_n__HE Cross-Tabular Freq Table Table of rezidivvain by Z_n__HE	
rezidivvain	Z_n__HE(Z#n# HE)	Total	
Frequency
Percent
Row Pct
Col Pct	0	1		
nein	22
32.84
56.41
52.38	17
25.37
43.59
68.00	39
58.21

	
ja	20
29.85
71.43
47.62	8
11.94
28.57
32.00	28
41.79

	
Total	42
62.69	25
37.31	67
100.00	


Statistics for Table of rezidivvain by Z_n__HE	


Chi-Square Tests Statistic	DF	Value	Prob	
Chi-Square	1	1.5716	0.2100	
Likelihood Ratio Chi-Square	1	1.5950	0.2066	
Continuity Adj. Chi-Square	1	0.9951	0.3185	
Mantel-Haenszel Chi-Square	1	1.5482	0.2134	
Phi Coefficient		-0.1532		
Contingency Coefficient		0.1514		
Cramer's V		-0.1532		


Fisher's Exact Test Fisher's Exact Test	
Cell (1,1) Frequency (F)	22	
Left-sided Pr <= F	0.1593	
Right-sided Pr >= F	0.9354	
		
Table Probability (P)	0.0948	
Two-sided Pr <= P	0.3060	

Sample Size = 67	


Table rezidivvain * Z_n__CIN Cross-Tabular Freq Table Table of rezidivvain by Z_n__CIN	
rezidivvain	Z_n__CIN(Z#n# CIN)	Total	
Frequency
Percent
Row Pct
Col Pct	0	1		
nein	22
32.84
56.41
68.75	17
25.37
43.59
48.57	39
58.21

	
ja	10
14.93
35.71
31.25	18
26.87
64.29
51.43	28
41.79

	
Total	32
47.76	35
52.24	67
100.00	


Statistics for Table of rezidivvain by Z_n__CIN	


Chi-Square Tests Statistic	DF	Value	Prob	
Chi-Square	1	2.7980	0.0944	
Likelihood Ratio Chi-Square	1	2.8263	0.0927	
Continuity Adj. Chi-Square	1	2.0300	0.1542	
Mantel-Haenszel Chi-Square	1	2.7563	0.0969	
Phi Coefficient		0.2044		
Contingency Coefficient		0.2002		
Cramer's V		0.2044		


Fisher's Exact Test Fisher's Exact Test	
Cell (1,1) Frequency (F)	22	
Left-sided Pr <= F	0.9732	
Right-sided Pr >= F	0.0768	
		
Table Probability (P)	0.0499	
Two-sided Pr <= P	0.1370	

Sample Size = 67	


Table rezidivvain * Multifokal Cross-Tabular Freq Table Table of rezidivvain by Multifokal	
rezidivvain	Multifokal	Total	
Frequency
Percent
Row Pct
Col Pct	nein		
nein	39
58.21
100.00
58.21	39
58.21

	
ja	28
41.79
100.00
41.79	28
41.79

	
Total	67
100.00	67
100.00	


Table rezidivvain * Condylome Cross-Tabular Freq Table Table of rezidivvain by Condylome	
rezidivvain	Condylome(Condylome)	Total	
Frequency
Percent
Row Pct
Col Pct	ja	nein		
nein	6
8.96
15.38
37.50	33
49.25
84.62
64.71	39
58.21

	
ja	10
14.93
35.71
62.50	18
26.87
64.29
35.29	28
41.79

	
Total	16
23.88	51
76.12	67
100.00	


Statistics for Table of rezidivvain by Condylome	


Chi-Square Tests Statistic	DF	Value	Prob	
Chi-Square	1	3.7057	0.0542	
Likelihood Ratio Chi-Square	1	3.6742	0.0553	
Continuity Adj. Chi-Square	1	2.6717	0.1021	
Mantel-Haenszel Chi-Square	1	3.6504	0.0561	
Phi Coefficient		-0.2352		
Contingency Coefficient		0.2289		
Cramer's V		-0.2352		


Fisher's Exact Test Fisher's Exact Test	
Cell (1,1) Frequency (F)	6	
Left-sided Pr <= F	0.0517	
Right-sided Pr >= F	0.9864	
		
Table Probability (P)	0.0381	
Two-sided Pr <= P	0.0811	

Sample Size = 67	


Table rezidivvain * HPV_Assoziation Cross-Tabular Freq Table Table of rezidivvain by HPV_Assoziation	
rezidivvain	HPV_Assoziation(HPV Assoziation)	Total	
Frequency
Percent
Row Pct
Col Pct	ja	nein		
nein	17
25.37
43.59
45.95	22
32.84
56.41
73.33	39
58.21

	
ja	20
29.85
71.43
54.05	8
11.94
28.57
26.67	28
41.79

	
Total	37
55.22	30
44.78	67
100.00	


Statistics for Table of rezidivvain by HPV_Assoziation	


Chi-Square Tests Statistic	DF	Value	Prob	
Chi-Square	1	5.1083	0.0238	
Likelihood Ratio Chi-Square	1	5.2233	0.0223	
Continuity Adj. Chi-Square	1	4.0445	0.0443	
Mantel-Haenszel Chi-Square	1	5.0321	0.0249	
Phi Coefficient		-0.2761		
Contingency Coefficient		0.2662		
Cramer's V		-0.2761		


Fisher's Exact Test Fisher's Exact Test	
Cell (1,1) Frequency (F)	17	
Left-sided Pr <= F	0.0215	
Right-sided Pr >= F	0.9944	
		
Table Probability (P)	0.0159	
Two-sided Pr <= P	0.0280	

Sample Size = 67	

Means Summary statistics Variable	Label	N	Mean	Std Dev	Minimum	Median	Maximum	
Alter_bei_Erk
ZeitDiagnoseCIN_VAIN
zeithysterP2
zeithysterP3
zeithysterP4
zeitfollowup
ZeitRezidiv_VaIN	Alter bei Erk


	67
32
41
12
29
50
28	53.4
1174.9
2875.4
5554.3
1766.9
389.2
467.6	14.1
1975.5
2783.7
3108.1
1710.7
304.8
324.4	26.0
-359.0
-31.0
612.0
-31.0
-8.0
98.0	53.0
413.0
1778.0
5428.0
1426.0
345.5
381.5	84.0
9087.0
11017.0
11017.0
8067.0
1378.0
1444.0	

Print Data Set WORK.A Obs	nr	ZeitDiagnoseCIN_VAIN	ED_VaIN	D_CIN	
1	1	.	27JUL2005	.	
2	2	369	04MAY2003	30APR2002	
3	3	.	15AUG2003	.	
4	4	1	31JUL2004	30JUL2004	
5	5	.	10JUN2003	.	
6	6	457	31MAY2003	28FEB2002	
7	7	.	13JAN2000	.	
8	8	.	02AUG2005	.	
9	9	.	25JUN2005	.	
10	10	20	30MAR2005	10MAR2005	
11	11	1307	30JUL2003	31DEC1999	
12	12	1426	26NOV2002	31DEC1998	
13	13	.	24AUG2004	.	
14	14	.	18DEC2003	.	
15	15	87	26NOV2005	31AUG2005	
16	16	.	11JAN2002	.	
17	17	.	26MAY2005	.	
18	18	.	23MAY2000	.	
19	19	.	28FEB2002	.	
20	20	561	14JUL2000	31DEC1998	
21	21	.	10MAY2003	.	
22	22	.	10AUG2004	.	
23	23	.	01MAY2004	.	
24	24	1230	14MAY2004	31DEC2000	
25	25	.	07SEP2002	.	
26	26	.	08JUN2004	.	
27	27	.	21SEP2004	.	
28	28	-28	15DEC2000	12JAN2001	
29	29	2511	15NOV2001	31DEC1994	
30	30	.	01FEB2003	.	
31	31	1640	28JUN2005	31DEC2000	
32	32	832	12APR2003	31DEC2000	
33	33	1	26JUN2003	25JUN2003	
34	34	546	14DEC2001	16JUN2000	
35	35	69	25OCT2005	17AUG2005	
36	36	.	09JAN1999	.	
37	37	289	09APR2004	25JUN2003	
38	38	6214	05JAN1999	31DEC1981	
39	39	.	17SEP1999	.	
40	40	234	11OCT2002	19FEB2002	
41	41	.	05MAR2004	.	
42	42	.	16JUN2004	.	
43	43	1778	13NOV2002	31DEC1997	
44	44	-21	10DEC2004	31DEC2004	
45	45	.	12FEB1999	.	
46	46	.	06APR2005	.	
47	47	.	06FEB2001	.	
48	48	1470	09JAN2003	31DEC1998	
49	49	.	16MAY2006	.	
50	50	.	27JUN2006	.	
51	51	.	03DEC2002	.	
52	52	.	20DEC2006	.	
53	53	0	20DEC2006	20DEC2006	
54	54	.	03APR2007	.	
55	55	.	06APR2007	.	
56	56	-359	04OCT2006	28SEP2007	
57	57	.	08MAY2007	.	
58	58	3867	03AUG2007	31DEC1996	
59	59	-19	21JUL2007	09AUG2007	
60	60	234	22AUG2007	31DEC2006	
61	61	973	11SEP2007	11JAN2005	
62	62	303	28JUL2007	28SEP2006	
63	63	.	17OCT2007	.	
64	64	.	01FEB2007	.	
65	65	9087	17NOV2007	31DEC1982	
66	66	-14	07DEC2007	21DEC2007	
67	67	2532	07DEC2007	31DEC2000	

Print Data Set WORK.A Obs	nr	zeithysterP2	zeithysterP3	zeithysterP4	Zeitpunkt_HE_anderer_Ursache	
1	1	8609	8609	.	31DEC1981	
2	2	369	.	369	.	
3	3	957	.	957	.	
4	4	.	.	.	.	
5	5	526	.	526	.	
6	6	457	.	457	.	
7	7	6587	6587	.	31DEC1981	
8	8	3136	3136	.	31DEC1996	
9	9	7116	7116	.	31DEC1985	
10	10	-1	.	-1	.	
11	11	1307	.	1307	.	
12	12	1426	.	1426	.	
13	13	3159	.	3159	.	
14	14	.	.	.	.	
15	15	.	.	.	.	
16	16	234	.	234	.	
17	17	.	.	.	.	
18	18	2700	.	2700	.	
19	19	11017	11017	.	31DEC1971	
20	20	561	.	561	.	
21	21	4878	4878	.	31DEC1989	
22	22	4606	.	4606	.	
23	23	.	.	.	.	
24	24	1230	.	1230	.	
25	25	3537	.	3537	.	
26	26	4543	4543	.	31DEC1991	
27	27	612	612	.	18JAN2003	
28	28	.	.	.	.	
29	29	2511	.	2511	.	
30	30	8067	.	8067	.	
31	31	1640	.	1640	.	
32	32	832	.	832	.	
33	33	.	.	.	.	
34	34	.	.	.	.	
35	35	.	.	.	.	
36	36	1105	.	1105	.	
37	37	.	.	.	.	
38	38	.	.	.	.	
39	39	1721	.	1721	.	
40	40	.	.	.	.	
41	41	.	.	.	.	
42	42	5281	5281	.	31DEC1989	
43	43	1778	.	1778	.	
44	44	.	.	.	.	
45	45	1869	.	1869	.	
46	46	.	.	.	.	
47	47	768	768	.	31DEC1998	
48	48	1470	.	1470	.	
49	49	.	.	.	.	
50	50	-31	.	-31	.	
51	51	.	.	.	.	
52	52	2241	.	2241	.	
53	53	.	.	.	.	
54	54	.	.	.	.	
55	55	5575	5575	.	31DEC1991	
56	56	.	.	.	.	
57	57	8529	8529	.	31DEC1983	
58	58	3867	.	3867	.	
59	59	.	.	.	.	
60	60	234	.	234	.	
61	61	.	.	.	.	
62	62	.	.	.	.	
63	63	.	.	.	.	
64	64	335	.	335	.	
65	65	.	.	.	.	
66	66	.	.	.	.	
67	67	2532	.	2532	.	

Obs	Zeitpunkt_HE__Ursache_CIN_CA_	ED_VaIN	zeitcervix	
1	.	27JUL2005	.	
2	30APR2002	04MAY2003	.	
3	31DEC2000	15AUG2003	957	
4	.	31JUL2004	.	
5	31DEC2001	10JUN2003	526	
6	28FEB2002	31MAY2003	.	
7	.	13JAN2000	.	
8	.	02AUG2005	.	
9	.	25JUN2005	.	
10	31MAR2005	30MAR2005	.	
11	31DEC1999	30JUL2003	.	
12	31DEC1998	26NOV2002	.	
13	31DEC1995	24AUG2004	3159	
14	.	18DEC2003	.	
15	.	26NOV2005	.	
16	22MAY2001	11JAN2002	234	
17	.	26MAY2005	.	
18	31DEC1992	23MAY2000	2700	
19	.	28FEB2002	.	
20	31DEC1998	14JUL2000	.	
21	.	10MAY2003	.	
22	31DEC1991	10AUG2004	4606	
23	.	01MAY2004	.	
24	31DEC2000	14MAY2004	.	
25	31DEC1992	07SEP2002	3537	
26	.	08JUN2004	.	
27	.	21SEP2004	.	
28	.	15DEC2000	.	
29	31DEC1994	15NOV2001	.	
30	31DEC1980	01FEB2003	8067	
31	31DEC2000	28JUN2005	.	
32	31DEC2000	12APR2003	.	
33	.	26JUN2003	.	
34	.	14DEC2001	.	
35	.	25OCT2005	.	
36	31DEC1995	09JAN1999	1105	
37	.	09APR2004	.	
38	.	05JAN1999	.	
39	31DEC1994	17SEP1999	1721	
40	.	11OCT2002	.	
41	.	05MAR2004	.	
42	.	16JUN2004	.	
43	31DEC1997	13NOV2002	.	
44	.	10DEC2004	.	
45	31DEC1993	12FEB1999	1869	
46	.	06APR2005	.	
47	.	06FEB2001	.	
48	31DEC1998	09JAN2003	.	
49	.	16MAY2006	.	
50	28JUL2006	27JUN2006	0	
51	.	03DEC2002	.	
52	31OCT2000	20DEC2006	2241	
53	.	20DEC2006	.	
54	.	03APR2007	.	
55	.	06APR2007	.	
56	.	04OCT2006	.	
57	.	08MAY2007	.	
58	31DEC1996	03AUG2007	.	
59	.	21JUL2007	.	
60	31DEC2006	22AUG2007	.	
61	.	11SEP2007	.	
62	.	28JUL2007	.	
63	.	17OCT2007	.	
64	03MAR2006	01FEB2007	349	
65	.	17NOV2007	.	
66	.	07DEC2007	.	
67	31DEC2000	07DEC2007	.	

Print Data Set WORK.A Obs	nr	zeitfollowup	ED_VaIN	Follow_Up1	Follow_Up2	Follow_Up3	
1	1	539	27JUL2005	07FEB2006	27OCT2006	17JAN2007	
2	2	.	04MAY2003	.	.	.	
3	3	679	15AUG2003	16SEP2004	11MAR2005	24JUN2005	
4	4	222	31JUL2004	10MAR2005	.	.	
5	5	276	10JUN2003	12SEP2003	05DEC2003	12MAR2004	
6	6	235	31MAY2003	23SEP2003	17DEC2003	21JAN2004	
7	7	.	13JAN2000	.	.	.	
8	8	.	02AUG2005	.	.	.	
9	9	370	25JUN2005	04JAN2006	20APR2006	30JUN2006	
10	10	393	30MAR2005	24AUG2005	02FEB2006	27APR2006	
11	11	483	30JUL2003	11FEB2004	05MAY2004	24NOV2004	
12	12	238	26NOV2002	25FEB2003	22APR2003	22JUL2003	
13	13	368	24AUG2004	26FEB2005	10JUN2005	27AUG2005	
14	14	180	18DEC2003	03APR2004	15JUN2004	.	
15	15	286	26NOV2005	20JAN2006	21APR2006	08SEP2006	
16	16	.	11JAN2002	.	.	.	
17	17	240	26MAY2005	26AUG2005	03NOV2005	21JAN2006	
18	18	556	23MAY2000	13DEC2000	23MAY2001	30NOV2001	
19	19	57	28FEB2002	26APR2002	.	.	
20	20	1338	14JUL2000	29AUG2001	28NOV2003	13MAR2004	
21	21	392	10MAY2003	04OCT2003	10FEB2004	05JUN2004	
22	22	396	10AUG2004	03MAR2005	10SEP2005	.	
23	23	76	01MAY2004	11OCT2003	03MAR2004	16JUL2004	
24	24	.	14MAY2004	.	.	.	
25	25	410	07SEP2002	07JAN2003	16JUL2003	22OCT2003	
26	26	659	08JUN2004	04JUL2005	26OCT2005	29MAR2006	
27	27	.	21SEP2004	.	.	.	
28	28	.	15DEC2000	.	.	.	
29	29	527	15NOV2001	10JAN2002	25JUL2002	26APR2003	
30	30	251	01FEB2003	15APR2003	15JUL2003	10OCT2003	
31	31	367	28JUN2005	31AUG2005	04MAR2006	30JUN2006	
32	32	279	12APR2003	17SEP2003	10DEC2003	16JAN2004	
33	33	.	26JUN2003	.	.	.	
34	34	1378	14DEC2001	08JUL2005	22SEP2005	.	
35	35	.	25OCT2005	.	.	.	
36	36	1355	09JAN1999	11JUL2001	22AUG2001	25SEP2002	
37	37	82	09APR2004	30JUN2004	.	.	
38	38	737	05JAN1999	07MAY1999	29SEP2000	11JAN2001	
39	39	.	17SEP1999	.	.	.	
40	40	-8	11OCT2002	26JUL2001	16FEB2002	03OCT2002	
41	41	204	05MAR2004	16JUL2004	25AUG2004	25SEP2004	
42	42	360	16JUN2004	03NOV2004	11MAR2005	11JUN2005	
43	43	397	13NOV2002	18FEB2003	13MAY2003	15DEC2003	
44	44	175	10DEC2004	10FEB2005	03MAR2005	03JUN2005	
45	45	230	12FEB1999	14MAR1999	30JUL1999	30SEP1999	
46	46	658	06APR2005	15DEC2005	28JUL2006	24JAN2007	
47	47	498	06FEB2001	18JAN2002	08MAR2002	19JUN2002	
48	48	523	09JAN2003	02SEP2003	13MAR2004	15JUN2004	
49	49	617	16MAY2006	20MAR2007	05SEP2007	23JAN2008	
50	50	374	27JUN2006	01DEC2006	30MAR2007	06JUL2007	
51	51	.	03DEC2002	.	.	.	
52	52	273	20DEC2006	01MAR2007	02MAY2007	19SEP2007	
53	53	.	20DEC2006	.	.	.	
54	54	421	03APR2007	20JUL2007	09OCT2007	28MAY2008	
55	55	60	06APR2007	05JUN2007	.	.	
56	56	331	04OCT2006	16MAR2007	12MAY2007	31AUG2007	
57	57	238	08MAY2007	31JUL2007	01JAN2008	.	
58	58	180	03AUG2007	07NOV2007	30JAN2008	.	
59	59	194	21JUL2007	15NOV2007	31JAN2008	.	
60	60	.	22AUG2007	.	.	.	
61	61	.	11SEP2007	.	.	.	
62	62	.	28JUL2007	.	.	.	
63	63	105	17OCT2007	30JAN2008	.	.	
64	64	237	01FEB2007	11APR2007	19JUN2007	26SEP2007	
65	65	.	17NOV2007	.	.	.	
66	66	.	07DEC2007	.	.	.	
67	67	25	07DEC2007	01JAN2008	.	.	

Print Data Set WORK.A Obs	nr	ZeitRezidiv_VaIN	Zeitpunkt_Rezidiv_VaIN	ED_VaIN	
1	1	195	07FEB2006	27JUL2005	
2	2	901	21OCT2005	04MAY2003	
3	3	680	25JUN2005	15AUG2003	
4	4	.	.	31JUL2004	
5	5	.	.	10JUN2003	
6	6	.	.	31MAY2003	
7	7	.	.	13JAN2000	
8	8	.	.	02AUG2005	
9	9	299	20APR2006	25JUN2005	
10	10	337	02MAR2006	30MAR2005	
11	11	.	.	30JUL2003	
12	12	800	03FEB2005	26NOV2002	
13	13	186	26FEB2005	24AUG2004	
14	14	182	17JUN2004	18DEC2003	
15	15	182	27MAY2006	26NOV2005	
16	16	.	.	11JAN2002	
17	17	254	04FEB2006	26MAY2005	
18	18	.	.	23MAY2000	
19	19	.	.	28FEB2002	
20	20	411	29AUG2001	14JUL2000	
21	21	427	10JUL2004	10MAY2003	
22	22	396	10SEP2005	10AUG2004	
23	23	.	.	01MAY2004	
24	24	.	.	14MAY2004	
25	25	.	.	07SEP2002	
26	26	233	27JAN2005	08JUN2004	
27	27	.	.	21SEP2004	
28	28	.	.	15DEC2000	
29	29	252	25JUL2002	15NOV2001	
30	30	758	28FEB2005	01FEB2003	
31	31	.	.	28JUN2005	
32	32	936	03NOV2005	12APR2003	
33	33	.	.	26JUN2003	
34	34	.	.	14DEC2001	
35	35	.	.	25OCT2005	
36	36	956	22AUG2001	09JAN1999	
37	37	.	.	09APR2004	
38	38	122	07MAY1999	05JAN1999	
39	39	.	.	17SEP1999	
40	40	.	.	11OCT2002	
41	41	376	16MAR2005	05MAR2004	
42	42	.	.	16JUN2004	
43	43	387	05DEC2003	13NOV2002	
44	44	.	.	10DEC2004	
45	45	273	12NOV1999	12FEB1999	
46	46	815	30JUN2007	06APR2005	
47	47	479	31MAY2002	06FEB2001	
48	48	.	.	09JAN2003	
49	49	.	.	16MAY2006	
50	50	.	.	27JUN2006	
51	51	1444	16NOV2006	03DEC2002	
52	52	.	.	20DEC2006	
53	53	.	.	20DEC2006	
54	54	.	.	03APR2007	
55	55	.	.	06APR2007	
56	56	477	24JAN2008	04OCT2006	
57	57	98	14AUG2007	08MAY2007	
58	58	.	.	03AUG2007	
59	59	.	.	21JUL2007	
60	60	.	.	22AUG2007	
61	61	.	.	11SEP2007	
62	62	.	.	28JUL2007	
63	63	.	.	17OCT2007	
64	64	238	27SEP2007	01FEB2007	
65	65	.	.	17NOV2007	
66	66	.	.	07DEC2007	
67	67	.	.	07DEC2007	

Print Data Set WORK.A Obs	nr	zeitcervix	D_Cervix_CA	ED_VaIN	
1	1	.	.	27JUL2005	
2	2	.	.	04MAY2003	
3	3	957	31DEC2000	15AUG2003	
4	4	.	.	31JUL2004	
5	5	526	31DEC2001	10JUN2003	
6	6	.	.	31MAY2003	
7	7	.	.	13JAN2000	
8	8	.	.	02AUG2005	
9	9	.	.	25JUN2005	
10	10	.	.	30MAR2005	
11	11	.	.	30JUL2003	
12	12	.	.	26NOV2002	
13	13	3159	31DEC1995	24AUG2004	
14	14	.	.	18DEC2003	
15	15	.	.	26NOV2005	
16	16	234	22MAY2001	11JAN2002	
17	17	.	.	26MAY2005	
18	18	2700	31DEC1992	23MAY2000	
19	19	.	.	28FEB2002	
20	20	.	.	14JUL2000	
21	21	.	.	10MAY2003	
22	22	4606	31DEC1991	10AUG2004	
23	23	.	.	01MAY2004	
24	24	.	.	14MAY2004	
25	25	3537	31DEC1992	07SEP2002	
26	26	.	.	08JUN2004	
27	27	.	.	21SEP2004	
28	28	.	.	15DEC2000	
29	29	.	.	15NOV2001	
30	30	8067	31DEC1980	01FEB2003	
31	31	.	.	28JUN2005	
32	32	.	.	12APR2003	
33	33	.	.	26JUN2003	
34	34	.	.	14DEC2001	
35	35	.	.	25OCT2005	
36	36	1105	31DEC1995	09JAN1999	
37	37	.	.	09APR2004	
38	38	.	.	05JAN1999	
39	39	1721	31DEC1994	17SEP1999	
40	40	.	.	11OCT2002	
41	41	.	.	05MAR2004	
42	42	.	.	16JUN2004	
43	43	.	.	13NOV2002	
44	44	.	.	10DEC2004	
45	45	1869	31DEC1993	12FEB1999	
46	46	.	.	06APR2005	
47	47	.	.	06FEB2001	
48	48	.	.	09JAN2003	
49	49	.	.	16MAY2006	
50	50	0	27JUN2006	27JUN2006	
51	51	.	.	03DEC2002	
52	52	2241	31OCT2000	20DEC2006	
53	53	.	.	20DEC2006	
54	54	.	.	03APR2007	
55	55	.	.	06APR2007	
56	56	.	.	04OCT2006	
57	57	.	.	08MAY2007	
58	58	.	.	03AUG2007	
59	59	.	.	21JUL2007	
60	60	.	.	22AUG2007	
61	61	.	.	11SEP2007	
62	62	.	.	28JUL2007	
63	63	.	.	17OCT2007	
64	64	349	17FEB2006	01FEB2007	
65	65	.	.	17NOV2007	
66	66	.	.	07DEC2007	
67	67	.	.	07DEC2007	
