# Supplementary material for: Detection of Human Papillomavirus Infection in Patients with Vaginal Intraepithelial Neoplasia
Source: PLoS One. 2016 Dec 1;11(12):e0167386. doi: 10.1371/journal.pone.0167386 (PMC5132291; doi:10.1371/journal.pone.0167386)
Supplement: S5 Table — (RTF) [file pone.0167386.s005.rtf]

Table of HPV_Assoziation by Z_n__CIN	
HPV_Assoziation(HPV Assoziation)	Z_n__CIN(Z#n# CIN)	
Frequency
Percent
Row Pct
Col Pct	0	1	Total	
0	19
28.36
51.35
59.38	18
26.87
48.65
51.43	37
55.22

	
1	13
19.40
43.33
40.63	17
25.37
56.67
48.57	30
44.78

	
Total	32
47.76	35
52.24	67
100.00	
Frequency Missing = 1	


Statistics for Table of HPV_Assoziation by Z_n__CIN	


Statistic	DF	Value	Prob	
Chi-Square	1	0.4269	0.5135	
Likelihood Ratio Chi-Square	1	0.4276	0.5132	
Continuity Adj. Chi-Square	1	0.1660	0.6837	
Mantel-Haenszel Chi-Square	1	0.4205	0.5167	
Phi Coefficient		0.0798		
Contingency Coefficient		0.0796		
Cramer's V		0.0798		

Effective Sample Size = 67
Frequency Missing = 1	

Table of HPV_Assoziation by Z__n__Cervix_CA	
HPV_Assoziation(HPV Assoziation)	Z__n__Cervix_CA(Z# n# Cervix CA)	
Frequency
Percent
Row Pct
Col Pct	0	1	Total	
0	6
8.96
16.22
42.86	31
46.27
83.78
58.49	37
55.22

	
1	8
11.94
26.67
57.14	22
32.84
73.33
41.51	30
44.78

	
Total	14
20.90	53
79.10	67
100.00	
Frequency Missing = 1	


Statistics for Table of HPV_Assoziation by Z__n__Cervix_CA	


Statistic	DF	Value	Prob	
Chi-Square	1	1.0946	0.2954	
Likelihood Ratio Chi-Square	1	1.0897	0.2965	
Continuity Adj. Chi-Square	1	0.5537	0.4568	
Mantel-Haenszel Chi-Square	1	1.0783	0.2991	
Phi Coefficient		-0.1278		
Contingency Coefficient		0.1268		
Cramer's V		-0.1278		

Effective Sample Size = 67
Frequency Missing = 1	

Table of HPV_Assoziation by Ursache_HE_andere_als_CIN	
HPV_Assoziation(HPV Assoziation)	Ursache_HE_andere_als_CIN(Ursache HE andere als CIN)	
Frequency
Percent
Row Pct
Col Pct	0	1	Total	
0	9
13.43
24.32
75.00	28
41.79
75.68
50.91	37
55.22

	
1	3
4.48
10.00
25.00	27
40.30
90.00
49.09	30
44.78

	
Total	12
17.91	55
82.09	67
100.00	
Frequency Missing = 1	


Statistics for Table of HPV_Assoziation by Ursache_HE_andere_als_CIN	


Statistic	DF	Value	Prob	
Chi-Square	1	2.3121	0.1284	
Likelihood Ratio Chi-Square	1	2.4250	0.1194	
Continuity Adj. Chi-Square	1	1.4404	0.2301	
Mantel-Haenszel Chi-Square	1	2.2776	0.1313	
Phi Coefficient		0.1858		
Contingency Coefficient		0.1826		
Cramer's V		0.1858		

Effective Sample Size = 67
Frequency Missing = 1	

Table of HPV_Assoziation by Ursache_HE_CIN_CA	
HPV_Assoziation(HPV Assoziation)	Ursache_HE_CIN_CA(Ursache HE CIN/CA)	
Frequency
Percent
Row Pct
Col Pct	0	1	Total	
0	14
20.90
37.84
48.28	23
34.33
62.16
60.53	37
55.22

	
1	15
22.39
50.00
51.72	15
22.39
50.00
39.47	30
44.78

	
Total	29
43.28	38
56.72	67
100.00	
Frequency Missing = 1	


Statistics for Table of HPV_Assoziation by Ursache_HE_CIN_CA	


Statistic	DF	Value	Prob	
Chi-Square	1	0.9982	0.3177	
Likelihood Ratio Chi-Square	1	0.9987	0.3176	
Continuity Adj. Chi-Square	1	0.5643	0.4525	
Mantel-Haenszel Chi-Square	1	0.9833	0.3214	
Phi Coefficient		-0.1221		
Contingency Coefficient		0.1212		
Cramer's V		-0.1221		

Effective Sample Size = 67
Frequency Missing = 1	

Table of Rezidiv_VaIN by Ursache_HE_andere_als_CIN	
Rezidiv_VaIN(Rezidiv VaIN)	Ursache_HE_andere_als_CIN(Ursache HE andere als CIN)	
Frequency
Percent
Row Pct
Col Pct	0	1	Total	
0	6
8.96
21.43
50.00	22
32.84
78.57
40.00	28
41.79

	
1	6
8.96
15.38
50.00	33
49.25
84.62
60.00	39
58.21

	
Total	12
17.91	55
82.09	67
100.00	
Frequency Missing = 1	


Statistics for Table of Rezidiv_VaIN by Ursache_HE_andere_als_CIN	


Statistic	DF	Value	Prob	
Chi-Square	1	0.4049	0.5245	
Likelihood Ratio Chi-Square	1	0.4007	0.5267	
Continuity Adj. Chi-Square	1	0.0982	0.7540	
Mantel-Haenszel Chi-Square	1	0.3989	0.5277	
Phi Coefficient		0.0777		
Contingency Coefficient		0.0775		
Cramer's V		0.0777		

Effective Sample Size = 67
Frequency Missing = 1	

Table of Rezidiv_VaIN by Ursache_HE_CIN_CA	
Rezidiv_VaIN(Rezidiv VaIN)	Ursache_HE_CIN_CA(Ursache HE CIN/CA)	
Frequency
Percent
Row Pct
Col Pct	0	1	Total	
0	14
20.90
50.00
48.28	14
20.90
50.00
36.84	28
41.79

	
1	15
22.39
38.46
51.72	24
35.82
61.54
63.16	39
58.21

	
Total	29
43.28	38
56.72	67
100.00	
Frequency Missing = 1	


Statistics for Table of Rezidiv_VaIN by Ursache_HE_CIN_CA	


Statistic	DF	Value	Prob	
Chi-Square	1	0.8839	0.3471	
Likelihood Ratio Chi-Square	1	0.8831	0.3473	
Continuity Adj. Chi-Square	1	0.4764	0.4901	
Mantel-Haenszel Chi-Square	1	0.8707	0.3508	
Phi Coefficient		0.1149		
Contingency Coefficient		0.1141		
Cramer's V		0.1149		

Effective Sample Size = 67
Frequency Missing = 1	

Table of Rezidiv_VaIN by Z__n__Cervix_CA	
Rezidiv_VaIN(Rezidiv VaIN)	Z__n__Cervix_CA(Z# n# Cervix CA)	
Frequency
Percent
Row Pct
Col Pct	0	1	Total	
0	7
10.45
25.00
50.00	21
31.34
75.00
39.62	28
41.79

	
1	7
10.45
17.95
50.00	32
47.76
82.05
60.38	39
58.21

	
Total	14
20.90	53
79.10	67
100.00	
Frequency Missing = 1	


Statistics for Table of Rezidiv_VaIN by Z__n__Cervix_CA	


Statistic	DF	Value	Prob	
Chi-Square	1	0.4903	0.4838	
Likelihood Ratio Chi-Square	1	0.4855	0.4859	
Continuity Adj. Chi-Square	1	0.1565	0.6924	
Mantel-Haenszel Chi-Square	1	0.4829	0.4871	
Phi Coefficient		0.0855		
Contingency Coefficient		0.0852		
Cramer's V		0.0855		

Effective Sample Size = 67
Frequency Missing = 1	

Table of Rezidiv_VaIN by Multifokalit_t	
Rezidiv_VaIN(Rezidiv VaIN)	Multifokalit_t(Multifokalität)	
Frequency
Percent
Row Pct
Col Pct	0	1	Total	
0	8
11.94
28.57
53.33	20
29.85
71.43
38.46	28
41.79

	
1	7
10.45
17.95
46.67	32
47.76
82.05
61.54	39
58.21

	
Total	15
22.39	52
77.61	67
100.00	
Frequency Missing = 1	


Statistics for Table of Rezidiv_VaIN by Multifokalit_t	


Statistic	DF	Value	Prob	
Chi-Square	1	1.0585	0.3036	
Likelihood Ratio Chi-Square	1	1.0469	0.3062	
Continuity Adj. Chi-Square	1	0.5354	0.4644	
Mantel-Haenszel Chi-Square	1	1.0427	0.3072	
Phi Coefficient		0.1257		
Contingency Coefficient		0.1247		
Cramer's V		0.1257		

Effective Sample Size = 67
Frequency Missing = 1	

Table of Multifokalit_t by Z_n__CIN	
Multifokalit_t(Multifokalität)	Z_n__CIN(Z#n# CIN)	
Frequency
Percent
Row Pct
Col Pct	0	1	Total	
0	6
8.96
40.00
18.75	9
13.43
60.00
25.71	15
22.39

	
1	26
38.81
50.00
81.25	26
38.81
50.00
74.29	52
77.61

	
Total	32
47.76	35
52.24	67
100.00	
Frequency Missing = 1	


Statistics for Table of Multifokalit_t by Z_n__CIN	


Statistic	DF	Value	Prob	
Chi-Square	1	0.4666	0.4946	
Likelihood Ratio Chi-Square	1	0.4697	0.4931	
Continuity Adj. Chi-Square	1	0.1519	0.6968	
Mantel-Haenszel Chi-Square	1	0.4596	0.4978	
Phi Coefficient		-0.0835		
Contingency Coefficient		0.0832		
Cramer's V		-0.0835		

Effective Sample Size = 67
Frequency Missing = 1	
