# Supplementary material for: Detection of Human Papillomavirus Infection in Patients with Vaginal Intraepithelial Neoplasia
Source: PLoS One. 2016 Dec 1;11(12):e0167386. doi: 10.1371/journal.pone.0167386 (PMC5132291; doi:10.1371/journal.pone.0167386)
Supplement: S6 Table — (DOCX) [file pone.0167386.s006.docx]

***Table 1: Sequence of GP-E6-3F consensus forwards Primer***

*^a^single letter code: W, A/T; K, G/T; R, A/G; Y, C/T; N, A/C/G/T; X, unknown nucleotide – to equal with consens sequence*

*^b^NP: not published*

| HPV  Genotype | 5’End-position | **GP-E6-3F** | | | | | | | 3’End-  position |
| --- | --- | --- | --- | --- | --- | --- | --- | --- | --- |
| Consensus |  | GGG | WGK | KAC | TGA | AAT | CGG | T |  |
| 6 | 27 | A - - | - | - | - | - | - | - | 607 |
| 11 | 27 | A - - | - | - | - | - | - | - | 607 |
| 16 | 27 | - | C - - | A - - | - | - - T | - | - | 636 |
| 31 | 31 | - | - | - | - | - - G | T - - | - | 634 |
| 33 | 32 | - | - | A - - | - | - - G | - | - | 647 |
| 35 | 32 | C - - | - C - | - | - | - | - | - | 636 |
| 43 | NP^b^ | - | - | - | C - - | - - A | - | - | NP |
| 52 | 31 | - | - | A - - | - | - | - | - | 627 |
| 58 | 33 | - | - | A - - | - | - | - | - | 648 |
| 61 | 24 | - - T | - | C - - | - | - | - | - | 592 |
| 64 | NP | - | X - - | - | - | - - T | - | - | NP |
| 67 | 24 | A - - | - | A - - | - | - | - | - | 637 |
| 72 | 31 | A - A | CAA | - | - | - | - | - | 598 |
| 13 | 29 | A - - | - | - | - | - | - | - | 607 |
| 18 | 35 | - | - | A - - | - | - | - | - | 674 |
| 30 | 29 | - | - | - | - | - - T | A - - | - | 644 |
| 32 | 25 | - | - | A - - | - | T - T | - | - | 632 |
| 34 | 25 | - | - | A - - | - | - | - | - | 629 |
| 39 | 36 | - | - | A - - | - | - | - | - | 679 |
| 40 | 42 | - | - | A - - | - | - | - | - | 602 |
| 42 | 37 | - | - | A - - | - | - TT | - | - | 626 |
| 44 | 30 | A - - | - | A - - | - | - TT | - | - | 611 |
| 45 | 32 | - | - | A - - | - | - | - | - | 674 |
| 51 | 39 | - - T | - A - | - | - | - | - | - | 638 |
| 53 | 29 | - | - | A - - | - | - - T | A - - | - | 647 |
| 54 | 29 | - | - | - | - | - | - | - | 611 |
| 55 | 43 | A - - | - | - | - | - TT | - | - | 608 |
| 56 | 29 | - | - | - | - | - | G - - | - | 650 |
| 57 | 35 | - | C - - | A - - | - | - | - | - | 608 |
| 59 | 33 | X - T | - AA | - | - | - | - | - | 659 |
| 62 | NP | - | G - - | - | - | - - T | - | - | NP |
| 66 | 29 | - | - | A - - | - | - - T | G - - | - | 650 |
| 68 | 3876 | - | - | - | - | - | - | - | 4516 |
| 69 | NP | - | X - X | X - - | - | - - T | - | - | NP |
| 70 | 34 | - | - | C - - | - | - | - | - | 679 |
| 71 | NP | - | X - X | X - - | - | - - T | - | G | NP |
| 73 | 41 | - | - | A - - | - | - | - | - | 628 |
| 74 | 2787 | - | X - X | X - - | - | - - T | - | - | 3451 |

***Table 2: Sequence of GP-E7-5B consensus reverse Primer***

| HPV  Genotype | 5’End-position | **GP-E7-5B** | | | | | | | | 3’End-  position |
| --- | --- | --- | --- | --- | --- | --- | --- | --- | --- | --- |
| Consensus |  | CTG | AGC | TGT | CAR | NTA | ATT | GCT | CA |  |
| 6 | 27 | - | - | - | - T - | C - - | - | - | - | 607 |
| 11 | 27 | - | - | - | - TT | C - - | - | - | - | 607 |
| 16 | 27 | - | - | - | - - T | T - - | - | - | - | 636 |
| 31 | 31 | - | - | - | - G - | G - - | - | - | - | 634 |
| 33 | 32 | - | - | - | - - C | T - - | - | - | - | 647 |
| 35 | 32 | - | - | - | - - C | AC - | - | - | - | 636 |
| 43 | NP | - | - | - | - - X | X - - | - | - | - | NP |
| 52 | 31 | - | - | - | - - C | C - - | - | - | - | 627 |
| 58 | 33 | - | - | - | - - C | A - - | - | - | - | 648 |
| 61 | 24 | - | - | - | - T - | A - - | - C - | - | - | 592 |
| 64 | NP | - | - | - | - | G - - | - | - | - | NP |
| 67 | 24 | - | - | - | - - T | GC- | - | - | - | 637 |
| 72 | 31 | - | - | - | - T - | G - - | - C - | - | - | 598 |

***Table 3: Sequence of GP-E7-6B consensus reverse Primer***

| HPV  Genotype | 5’End-  position | **GP-E7-6B** | | | | | | | | 3’End-  position |
| --- | --- | --- | --- | --- | --- | --- | --- | --- | --- | --- |
| Consensus |  | TCC | TCT | GAG | TYG | YCT | AAT | TGC | TC |  |
| 13 | 29 | - - T | - | - | C - - | - | - | - | - | 607 |
| 18 | 35 | - | - | - | - | - T - | - | - | - | 674 |
| 30 | 29 | - | - | - | C - - | - TC | - | - | - | 644 |
| 32 | 25 | - - A | - | - | G - - | - - A | - | - - T | - | 632 |
| 34 | 25 | - | - | - | - | - | - | GA - | - | 629 |
| 39 | 36 | - | - | - - C | - - T | - | - | - | - | 679 |
| 40 | 42 | GAG | - | - | C - - | - | - | - | - | 602 |
| 42 | 37 | - - A | - | - | C - - | - - C | - | - - T | - | 626 |
| 44 | 30 | - - T | - | - | C - - | - | - | - | - | 611 |
| 45 | 32 | - | - | - - C | - | - T - | - | - | - | 674 |
| 51 | 39 | - | - | - | C - - | - - A | - | - | - | 638 |
| 53 | 29 | - | - | - | C - - | - TC | - | - | - | 647 |
| 54 | 29 | G- A | - - A | - | - - T | - | - | - | - | 611 |
| 55 | 43 | - | - | - | C - - | - | - | - | - | 608 |
| 56 | 29 | - | - | - | C - - | - - C | - | - | - | 650 |
| 57 | 35 | - - T | - | - | - | - - A | - | - | - | 608 |
| 59 | 33 | GAG | - - G | - | - - A | GG- | - | - | - | 659 |
| 62 | NP | - | - | - | - | - | - | - | - | NP |
| 66 | 29 | - | - | - | C - - | - - C | - | - | - | 650 |
| 68 | 3876 | - - G | - | - - A | - - T | - | - | - | - | 4516 |
| 69 | NP | - | - | - | - X - | X - - | - | - | - | NP |
| 70 | 34 | - TG | - | - - A | - - T | - | - | - | - | 679 |
| 71 | NP | - - X | TXX | XA - | - | - | - | - | - | NP |
| 73 | 41 | - | - | - | - | - - C | - | GA - | - | 628 |
| 74 | 2787 | - | - | - | - | - | - | - | - | 3451 |

***Table 4: Sequence of type specific primer (Cocktail I)***

| Primer Cocktail | HPV  Type | Amplicon  (bp) | Sequence (5’-3’) | Position |
| --- | --- | --- | --- | --- |
| I | 16 | 457 | CAC AGT TAT GCA CAG AGC TGC | 141-161 |
|  |  |  | CAT ATA TTC ATG CAA TGT AGG TGT A | 597-573 |
|  | 18 | 322 | CAC TTC ACT GCA AGA CAT AGA | 170-190 |
|  |  |  | GTT GTG AAA TCG TCG TTT TTC A | 491-470 |
|  | 31 | 263 | GAA ATT GCA TGA ACT AAG CTC G | 137-158 |
|  |  |  | CAC ATA TAC CTT TGT TTG TCA A | 399-378 |
|  | 59 | 215 | CAA AGG GGA ACT GCA AGA AAG | 159-179 |
|  |  |  | TAT AAC AGC GTA TCA GCA GC | 373-354 |
|  | 45 | 151 | GTG GAA AAG TGC ATT ACA GG | 82-101 |
|  |  |  | ACC TCT GTG CGT TCC AAT GT | 232-213 |

***Table 5: Sequence of type specific primer (Cocktail II)***

| Primer Cocktail | HPV  Type | Amplicon  (bp) | Sequence (5’-3’) | Position |
| --- | --- | --- | --- | --- |
| II | 33 | 398 | ACT ATA CAC AAC ATT GAA CTA | 172-192 |
|  |  |  | GTT TTT ACA CGT CAC AGT GCA | 569-549 |
|  | 6/11 | 334 | TGC AAG AAT GCA CTG ACC AC | 201-220 |
|  |  |  | TGC ATG TTG TCC AGC AGT GT | 534-515 |
|  | 58 | 274 | GTA AAG TGT GCT TAC GAT TGC | 297-317 |
|  |  |  | GTT GTT ACA GGT TAC ACT TGT | 570-550 |
|  | 52 | 229 | TAA GGC TGC AGT GTG TGC AG | 178-197 |
|  |  |  | CTA ATA GTT ATT TCA CTT AAT GGT | 406-383 |
|  | 56 | 181 | GTG TGC AGA GTA TGT TTA TTG | 294-314 |
|  |  |  | TTT CTG TCA CAA TGC AAT TGC | 475-455 |

***Table 6: Sequence of type specific primer (Cocktail III)***

| Primer Cocktail | HPV  Type | Amplicon  (bp) | Sequence (5’-3’) | Position |
| --- | --- | --- | --- | --- |
| III | 35 | 358 | CAA CGA GGT AGA AGA AAG CAT C | 157-178 |
|  |  |  | CCG ACC TGT CCA CCG TCC ACC G | 514-493 |
|  | 42 | 277 | CCC AAA GTA GTG GTC CCA GTT A | 85-106 |
|  |  |  | GAT CTT TCG TAG TGT CGC AGT G | 361-340 |
|  | 43 | 219 | GCA TAA TGT CTG CAC GTA GCT G | 102-123 |
|  |  |  | CAT GAA ACT GTA GAC AGG CCA AG | 320-298 |
|  | 44 | 163 | TAA ACA GTT ATA TGT AGT GTA CCG | 248-271 |
|  |  |  | TAT CAG CAC GTC CAG AAT TGA C | 410-389 |

***Table 7: Sequence of type specific primer (Cocktail IV)***

| Primer Cocktail | HPV  Type | Amplicon  (bp) | Sequence (5’-3’) | Position |
| --- | --- | --- | --- | --- |
| IV | 68 | 333 | GCA GAA GGC AAC TAC AAC GG | 4049-4068 |
|  |  |  | GTT TAC TGG TCC AGC AGT GG | 4381-4362 |
|  | 39 | 280 | GAC GAC CAC TAC AGC AAA CC | 213-232 |
|  |  |  | TTA TGA AAT CTT CGT TTG CT | 492-473 |
|  | 51 | 223 | GAG TAT AGA CGT TAT AGC AGG | 319-339 |
|  |  |  | TTT CGT TAC GTT GTC GTG TAC G | 541-520 |
|  | 66 | 172 | TTC AGT GTA TGG GGC AAC AT | 353-372 |
|  |  |  | AAA CAT GAC CCG GTC CAT GC | 520-501 |

VaIN-Cases and HPV type (data pseudonymised):

1 G-5XX3/09 HPV 16 25 G-2XX7/08 HPV 56

2 G-6XX7/09 26 G-3XX3/06

3 G-5XX9/08 HPV 16 27 G-5X1/09 HPV 16

4 G-3XX4/08 HPV 16 28 G-4XX2/08 HPV 16

5 G-2XX1/07 29 G-4XX3/04

6 G-2XX7/04 30 G-4XX7/05 HPV 16

7 G-1X7/04 HPV 16 31 G-4X2/07 HPV 33

8 G-8X9/05 HPV 16 32 G-3XX8/09 HPV 16

9 G-4XX0/09 33 G-1XX5/07 HPV 16,6/11 und 68

10 G-3X1/09 HPV 16 34 G-2XX7/07 HPV 56

11 G-1XX6/09 HPV 16 35 G-4XX2/05

12 G-3XX0/07 HPV 18 36 G-5XX4/09 HPV 16

13 G-5XX1/06 37 G-8X/03 HPV 16

14 G-3XX1/06 HPV 16 38 G-1XX9/08 HPV 33

15 G-2XX5/08 HPV 16 39 G-4XX1/02 HPV 16

16 G-6XX9/09 HPV 16 40 G-3XX4/03 HPV 16

17 G-7X/06 41 G-4XX6/06 HPV 16

18 G-2XX3/09 HPV 52 42 G-1XX3/09 HPV 16

19 G-1XX8/04 43 G-2XX0/08

20 G-4X8/07 44 G-5XX2/06 HPV 16

21 G-3XX3/05 45 G-5XX9/08 HPV 16

22 G-2XX5/07 HPV 35 46 G-5X5/03

23 G-4XX2/08 47 G-1XX4/09

24 G-1XX1/08 48 G-4X8/07


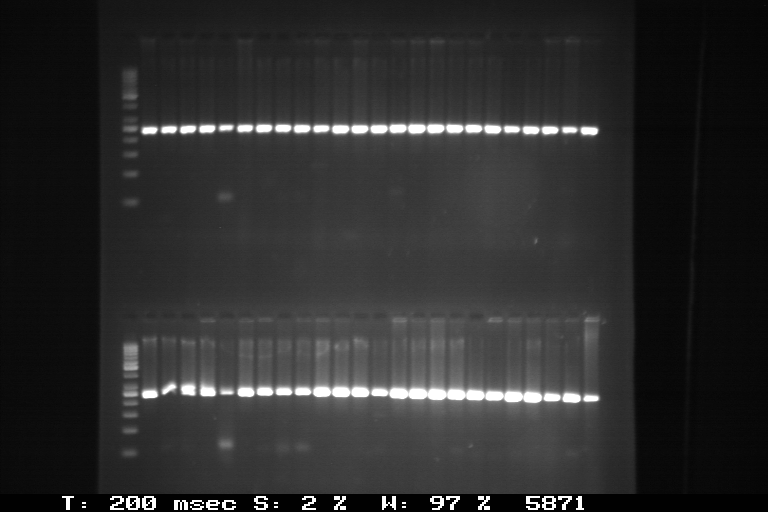


Figure 1: Extraction control

Not all Figures shown.


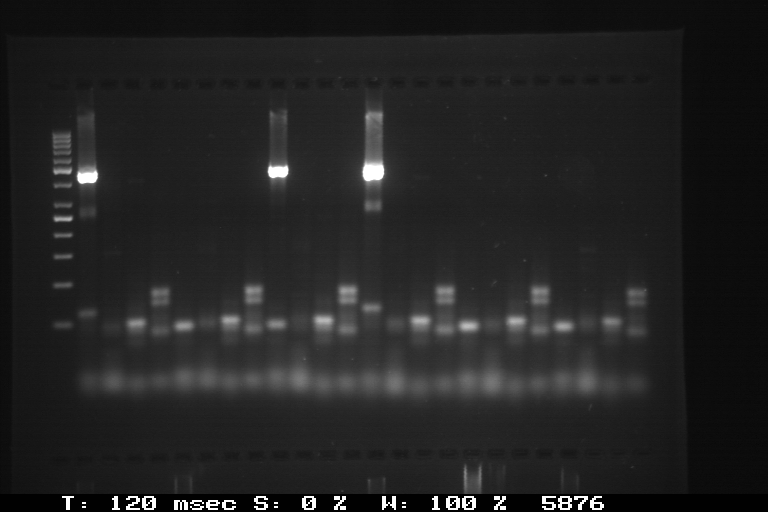


Figure 2: Case 1-6


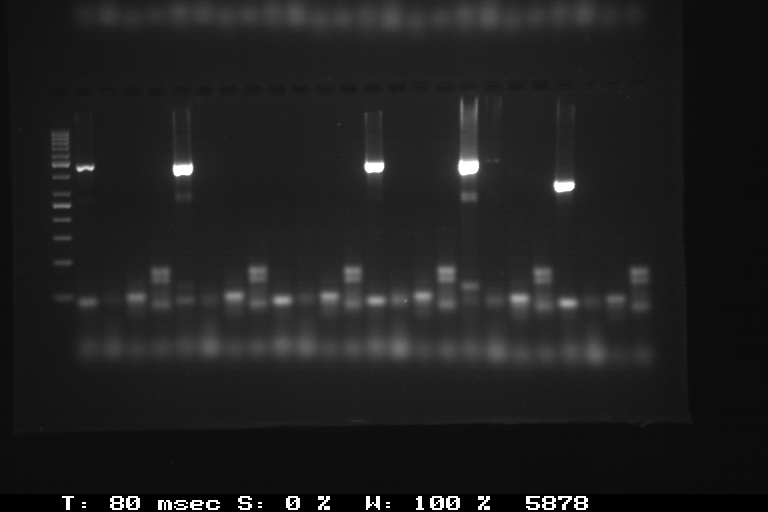


Figure 3: Case 7-12


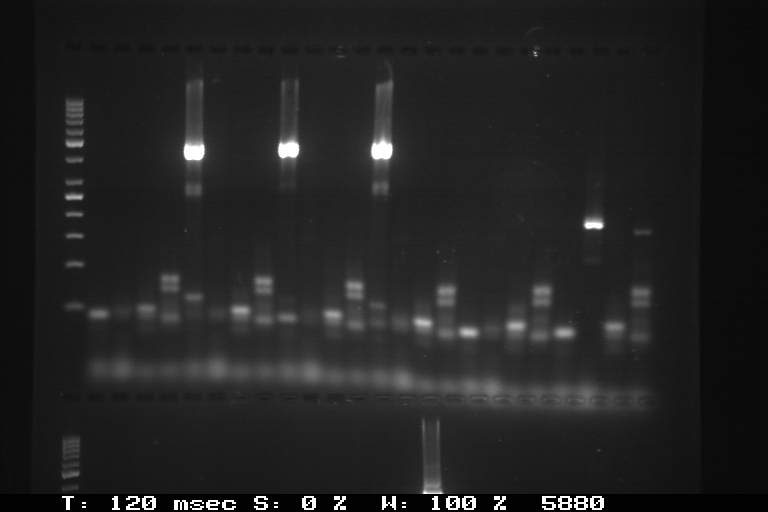


Figure 4: Case 13-18


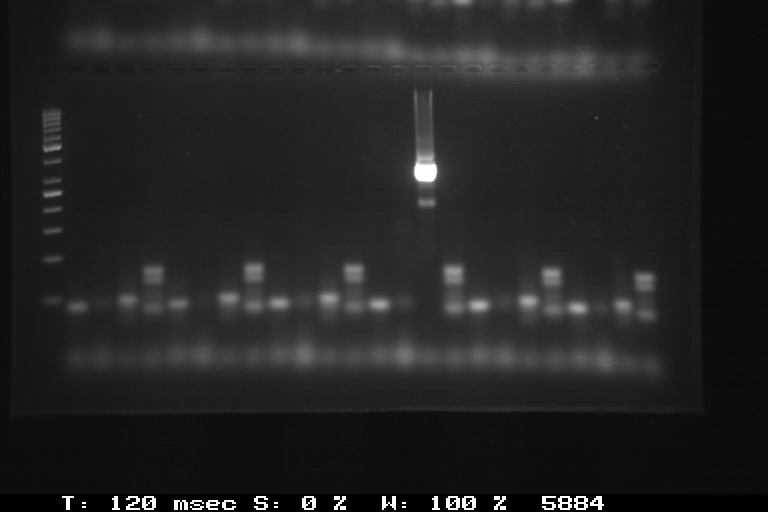


Figure 5: Case 19-24


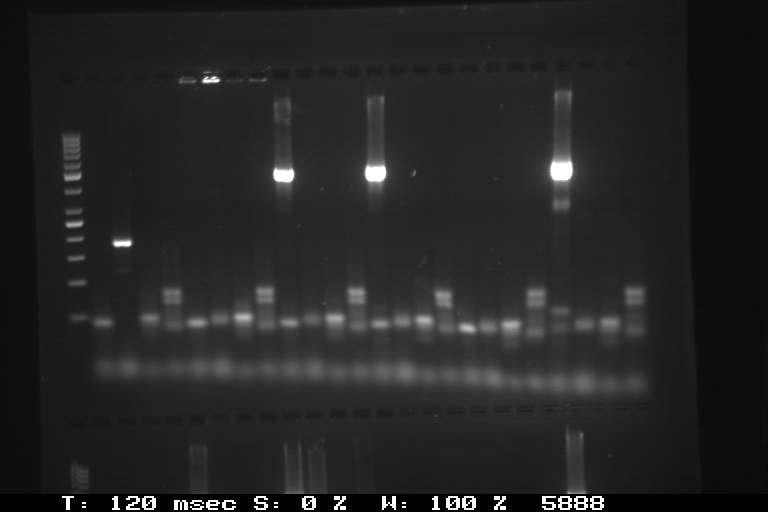


Figure 6: Case 25-30


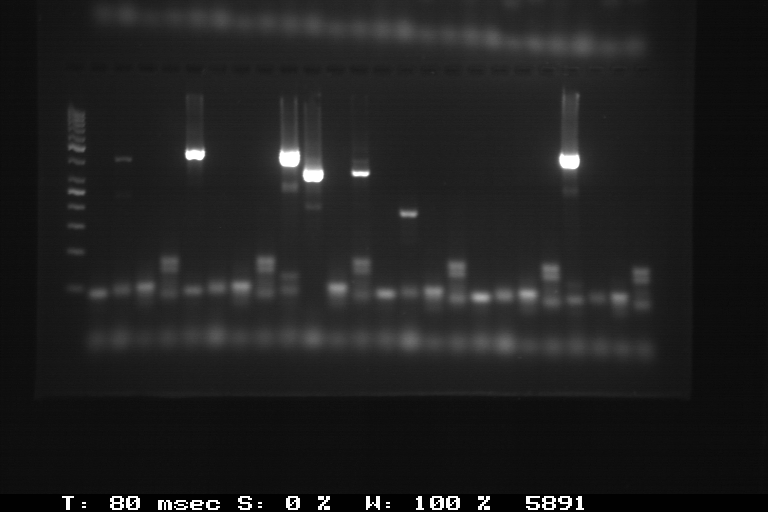


Figure 7: Case 31-36


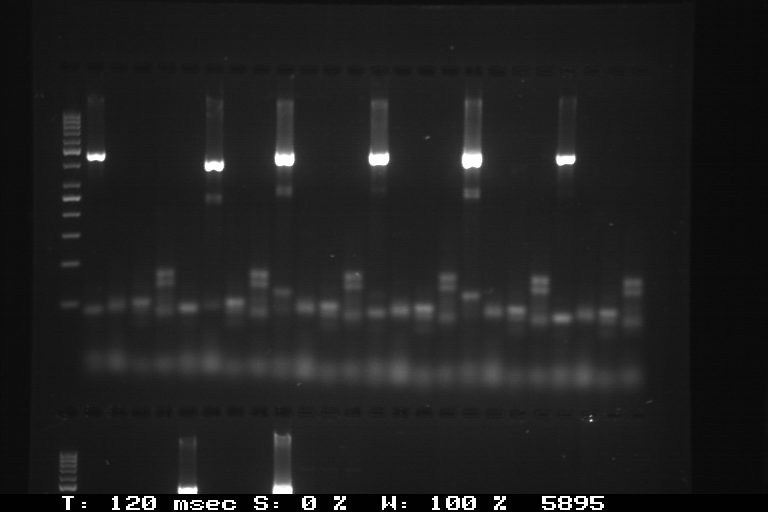


Figure 8: Case 37-42


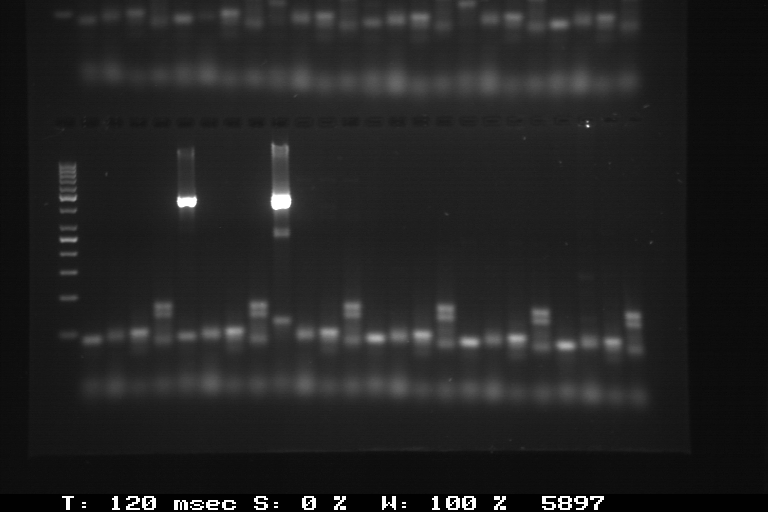


Figure 9: Case 43-48
